# New Ceramides and Cerebrosides from the Deep-Sea Far Eastern Starfish *Ceramaster patagonicus*

**DOI:** 10.3390/md20100641

**Published:** 2022-10-14

**Authors:** Timofey V. Malyarenko, Viktor M. Zakharenko, Alla A. Kicha, Alexandra S. Kuzmich, Olesya S. Malyarenko, Anatoly I. Kalinovsky, Roman S. Popov, Vasily I. Svetashev, Natalia V. Ivanchina

**Affiliations:** 1 G.B. Elyakov Pacific Institute of Bioorganic Chemistry, Far Eastern Branch, Russian Academy of Sciences, Pr. 100-let Vladivostoku 159, 690022 Vladivostok, Russia; 2 Department of Bioorganic Chemistry and Biotechnology, School of Natural Sciences, Far Eastern Federal University, Russky Island, Ajax Bay 10, 690922 Vladivostok, Russia; 3 A.V. Zhirmunsky National Scientific Center of Marine Biology, Far Eastern Branch, Russian Academy of Sciences, ul. Palchevskogo 17, 690041 Vladivostok, Russia

**Keywords:** ceramides, cerebrosides, NMR spectra, fatty acids, long-chain bases, starfish, *Ceramaster patagonicus*, cytotoxic activity, inhibition of colony formation

## Abstract

Three new ceramides (**1**–**3**) and three new cerebrosides (**4**, **8**, and **9**), along with three previously known cerebrosides (ophidiocerebrosides C (**5**), D (**6**), and CE-3-2 (**7**)), were isolated from a deep-sea starfish species, the orange cookie starfish *Ceramaster patagonicus*. The structures of **1**−**4,** **8**, and **9** were determined by the NMR and ESIMS techniques and also through chemical transformations. Ceramides **1**–**3** contain *iso-*C_21_ or C_23_ Δ^9^-phytosphingosine as a long-chain base and have C_16_ or C_17_ (2*R*)-2-hydroxy-fatty acids of the normal type. Cerebroside **4** contains C_22_ Δ^9^-sphingosine *anteiso-*type as a long-chain base and (2*R*)-2-hydroxyheptadecanoic acid of the normal type, while compounds **8** and **9** contain saturated C-17 phytosphingosine *anteiso-*type as a long-chain base and differ from each other in the length of the polymethylene chain of (2*R*)-2-hydroxy-fatty acids of the normal type: C_23_ in **8** and C_24_ in **9**. All the new cerebrosides (**4**, **8**, and **9**) have β-D-glucopyranose as a monosaccharide residue. The composition of neutral sphingolipids from *C. patagonicus* was described for the first time. The investigated compounds **1**–**3**, **5**–**7**, and **9** exhibit slight to moderate cytotoxic activity against human cancer cells (HT-29, SK-MEL-28, and MDA-MB-231) and normal embryonic kidney cells HEK293. Compounds **2**, **5**, and **6** at a concentration of 20 µM inhibit colony formation of MDA-MB-231 cells by 68%, 54%, and 68%, respectively. The colony-inhibiting activity of compounds **2**, **5**, and **6** is comparable to the effect of doxorubicin, which reduces the number of colonies by 70% at the same concentration.

## 1. Introduction

Starfish (also called sea stars) are found throughout the world’s oceans at a wide range of depths: from intertidal to deep-sea habitats. Their ecological characteristics and life-history features make them a rich source of various low-molecular-weight compounds. The best-studied starfish-derived substances are polar steroidal compounds that were found in almost all of the species analyzed [1,2,3,4,5,6,7,8,9]. In addition, anthraquinoid pigments, triterpene glycosides, carotenoids, and sphingolipids were found in starfish [5,9,10,11,12,13,14,15].

Sphingolipids are a group of heterogeneous lipids including those present in the plasma membranes that, along with phospholipids and sterols, play a fundamental role in important phenomena such as cell–cell recognition and antigenic specificity [16,17]. Sphingolipids can be divided into several structural groups: ceramides, cerebrosides, gangliosides, and sphingophospholipids.

Ceramides are hydrophobic molecules consisting of a long-chain base (LCB) and an amide-linked fatty acid (FA) residue. Ceramides are biosynthesized during the reaction of S-acyl-coenzyme A (usually C_16_-CoA) with serine, which is catalyzed by serine palmitoyl transcriptase or related enzymes, followed by the reduction of the carbonyl group by ketosphinganine reductase and the N-acylation by ceramide synthase. Surprisingly, the LCB hydroxylation, which leads to the production of so-called phytosphinganine derivatives, also occurs in plants and many echinoderms. When hydroxylases act on FA in these invertebrates, an additional hydroxyl group is introduced also into the α–position of FA [17]. Additionally, both bases and FA moieties in this type of natural product may contain normal chains, as well as those with *iso-* and/or *anteiso-*branching. Unfortunately, the biological activity of starfish-derived ceramides has not been sufficiently studied. It was previously reported that asteriaceramide A from *Asterias amurensis* showed it could actively stimulate the root growth of *Brassica campestris* [18].

Cerebrosides are glycosylceramides containing, as a rule, glucose and galactose or other rare monosaccharide residues in their carbohydrate moieties. These compounds are synthesized by special enzymes, glycosyl-transferases, that attach monosaccharide residues to C-1 of ceramide [17]. On the basis of the chemical structure of cerebrosides, these can be divided into three groups: monoglycosides, biglycosides (mainly lactosides), and oligoglycosides. In addition to glucose and galactose residues, this class of glycosylated lipids can contain an aminosugar residue (globosides) in their carbohydrate moieties or be sulfated. The interest in sphingolipids and their derivatives is mainly associated with their wide range of biological activities. Some studies have shown that sphingolipids can inhibit the growth of microalgae, fungi, and bacteria [19]. It was previously reported that starfish cerebrosides showed neuritogenic activity against the rat pheochromocytoma PC12 cells in the presence of nerve growth factor (NGF), an anti-inflammatory effect, in vitro cytotoxic activity against Caco-2 colon cancer cells, improvement of the barrier function of the skin, and other properties [14].

Thus, the study of starfish sphingolipids is an interesting and relevant scientific issue. It is also worth noting that previously, sphingolipids were studied from starfish that live at shallow depths. To date, there have been no reports on sphingolipids derived from starfish dwelling at depths greater than 150 m.

Recently, we found that conjugates of polyhydroxysteroids with long-chain FAs from the same starfish species exhibited potent anticancer activity in vitro [20]. In the present report, continuing the search for anticancer compounds from marine organisms, we provide the results of our studies on the structures of ceramides and cerebrosides derived from the starfish *Ceramaster patagonicus*, and also their effects on the viability of human normal and cancer cells and the colony formation of cancer cells.

## 2. Results and Discussion

### 2.1. The Isolation and Determination of the Structures of Compounds ***1**–**9*** from C. patagonicus

**Figure 1:**
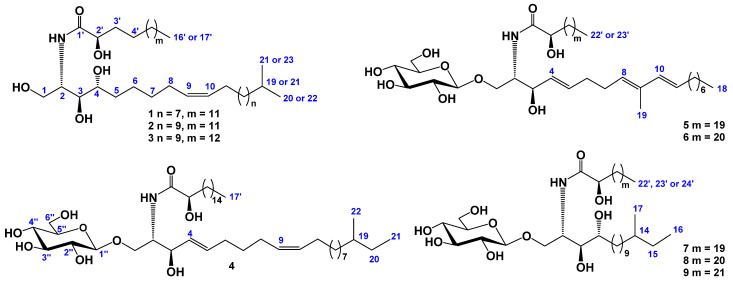
The structures of compounds **1**–**9** isolated from *C. patagonicus*.

The concentrated methanol–chloroform–ethanolic extract of *C. patagonicus* was separated between H_2_O and AcOEt/BuOH, with the organic layer dried and washed with cold acetone. The acetone-soluble fraction was separated by chromatography on a silica gel column, followed by HPLC on semi-preparative Diasfer-110-C18, Discovery C18, and Discovery HS C18-10 columns. As a result, we obtained three new ceramides (**1**–**3**) and three new cerebrosides (**4**, **8**, and **9**) along with three known cerebrosides: ophidiacerebrosides C (**5**) and D (**6**) that had been previously isolated from the purple starfish *Ophidiaster ophidianus* [21] and CE-3-2 (**7**) from the sea cucumber *Cucumaria echinata* [22] (Figure 1).

**Figure 2:**
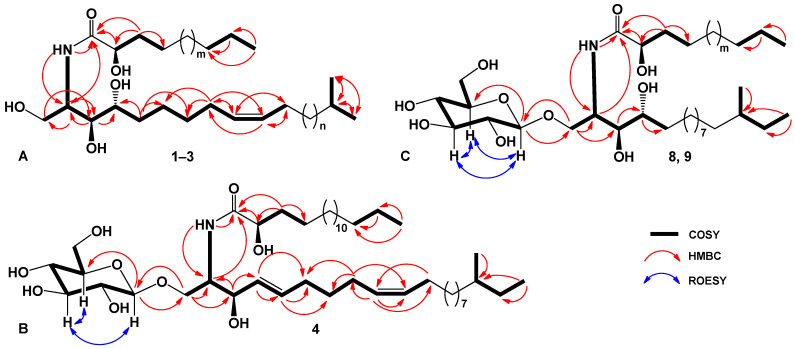
^1^H-^1^H COSY and key HMBC correlations for compounds **1**–**3** (**A**) and ^1^H-^1^H COSY, key HMBC, and ROESY correlations for compounds **4** (**B**), **8**, and **9** (**C**).

The IR spectrum of compound **1** showed the presence of hydroxyl (3402 cm^−1^) and amide (1656, 1523 cm^−1^) groups. The molecular formula of compound **1** was determined as C_37_H_73_NO_5_ from the [M + Na]^+^ sodium adduct ion peak at *m*/*z* 634.5376 in the (+)HRESIMS and the [M – H]^−^ deprotonated molecular ion peak at *m*/*z* 610.5412 in the (–)HRESIMS (Figures S1 and S2). The ^1^H- and ^13^C-NMR spectroscopic data of **1** showed the resonances of protons and carbons of three terminal methyls CH_3_-20, CH_3_-21, and CH_3_-16′ [*δ*_H_ 2 × 0.88 d (6.5), 0.89 t (7.2); *δ*_C_ 2 × 22.5, 14.0], four oxygenated groups CH_2_-1 [*δ*_Ha_ 4.51 dt (10.9, 4.2), *δ*_Hb_ 4.43 m; *δ*_C_ 61.8], CH-3 (*δ*_H_ 4.34 m; *δ*_C_ 76.7), CH-4 (*δ*_H_ 4.28 m; *δ*_C_ 72.8), and CH-2′ (*δ*_H_ 4.62 m; *δ*_C_ 72.3), one amide group NH-CO [*δ*_H_ 8.55 d (9.0); *δ*_C_ 175.0], the 9(10)-double bond (*δ*_H_ 5.50 m, 5.48 m; *δ*_C_ 130.1, 129.9), and one characteristic methine CH-2 [*δ*_H_ 5.10 sext (4.5); *δ*_C_ 52.8] attached at the nitrogen atom (Table 1, Figures S3 and S4). Thus, the ^1^H- and ^13^C-NMR spectra of **1** exhibited the characteristic signals of an unsaturated phytosphingosine-type ceramide with a 2-hydroxy fatty acid (Figure 1). Moreover, ceramide **1** has normal and *iso-*types of side chains; in the terminal methyl groups, the carbon atom signals were observed at *δ*_C_ 14.0 (normal form) and 2 × 22.5 (*iso-*form) in the ^13^C-NMR spectrum (Table 1, Figure S4). The ^1^H-^1^H COSY and HSQC correlations of **1** revealed the corresponding sequences of protons at C-1 to C-11; C-20 to C-21 through C-19; C-2 to NH; C-2′ to C-4′, and C-16′ to C-14′ (Table 1, Figure 2A, Figures S5 and S6). The key HMBC cross-peaks, such as Hb-1/C-3; H-2/C-1, C-3, C-1′; H-3/C-2, C-4; Hb-5/C-4, C-6, C-7; H-8/C-6, C-7, C-9, C-10; H-9 and H-10/C-8, C-11; H-11/C-9, C-10; H-19/C-20, C-21; H_3_-20/C-19, C-21; H_3_-21/C-19, C-20; NH/C-2, C-1′; H-2′/C-1′; Ha-3′/C-1′, C-4′; Hb-3′/C-2′; H_2_-15′/C-14′; and H_3_-16′/C-14′, C-15′ confirmed the overall structure of ceramide **1** (Figure 2A and Figure S7).

The polymethylene chain length of LCB and FA and the absolute configuration of the ceramide **1** were determined as follows. When **1** was methanolyzed with methanolic hydrochloric acid, fatty acid methyl ester (FAME) was obtained together with LCB. A gas chromatography–mass spectrometry (GC–MS) analysis of FAME showed the existence of one component that was characterized as saturated methyl 2-hydroxyhexadecanoate of normal type (FAME-1). The normal type of FAME-1 was also confirmed by ^1^H-NMR spectra, which consisted only of one triplet terminal methyl group at *δ*_H_ 0.89. The optical rotation of FAME-1 ([α]_D_^25^ –3.5˚ (*c* = 1.0, CHCl_3_)) is consistent with the data [α]_D_^25^ –3.21˚ reported in the literature [23]; therefore, the absolute stereochemistry at C-2′ is suggested to be *R*. Based on this suggestion, as well as on NMR and mass spectrometric data, we assumed LCB of ceramide **1** to have 21 carbon atoms and *iso-*type of unsaturated polymethylene chain. The geometry of the double bond in LCB can be determined on the basis of the ^13^C-NMR chemical shift of the methylene carbon adjacent to the olefinic carbon (*δ*_C_ ≈ 27 for (*Z*) isomers and *δ*_C_ ≈ 32 for (*E*) isomers [24]). The ^13^C-NMR spectrum of compound **1** indicated the presence of two characteristic allyl carbons, C-8 (*δ*_C_ 27.5) and C-11 (*δ*_C_ 27.3). Thus, the olefinic group in **1** was determined to have a *cis* (*Z*) geometry. The location of the double bond in the LCB moiety at position C-9 was determined through ^1^H-^1^H COSY, HMBC, and 2D TOCSY NMR experiments (Figure 2A, Figure S5, S7, and S8). The absolute configuration of LCB of ceramide **1** was assumed to be *D-ribo-*(2*S*,3*S*,4*R*) on the basis of similarities in optical rotation ([α]_D_^25^ +28.0˚ (*c* = 1.0, CHCl_3_)) with synthetic *D-ribo-*(2*S*,3*S*,4*R*)-phytosphingosine ([α]_D_^25^ +26.8˚ (*c* = 1.1, CHCl_3_)) [25].

Based on all above-mentioned data, we determined the structure of **1** to be (2*S*,3*S*,4*R*,9*Z*)-2-[(2*R*)-2-hydroxyhexadecanoylamino]-19-methyl-9-icosen-1,3,4-triol. As far as we know, a ceramide with such a chemical structure was isolated for the first time.

The IR spectrum of compound **2** showed the presence of hydroxyl (3404 cm^−1^) and amide (1657, 1522 cm^−1^) groups. The molecular formula of compound **2** was determined as C_39_H_77_NO_5_ from the [M + Na]^+^ sodium adduct ion peak at *m*/*z* 662.5324 in the (+)HRESIMS and the [M – H]^−^ deprotonated molecular ion peak at *m*/*z* 638.5364 in the (–)HRESIMS (Figures S9 and S10). A comparison of the ^1^H-, ^13^C-NMR spectra and an extensive 2D NMR analysis of compounds **1**, **2**, and **3** revealed that the unsaturated phytosphingosine-type ceramide with a 2-hydroxy fatty acid of **2** and **3** is identical to that of compound **1**, while the polymethylene chain lengths of LCB and/or FA of **1**–**3** differ from each other (Figure 1, Figures S11–S15, Table 1). A comparison of the molecular weights (MWs) of **1** and **2** showed that they differed by 28 amu.

The FA unit in **2** was identified by GC analysis and the mass spectra of the FAME-2 derivative were measured by GC–MS similarly to compound **1**. The GC–MS analysis showed that FAME-2 was identical to FAME-1. Moreover, the normal type of FAME-2 was also confirmed by the ^1^H-NMR spectrum, which consisted of only one triplet terminal methyl group at *δ*_H_ 0.89. Thus, the FA of ceramide **2** was determined to be (2*R*)-2-hydroxyhexadecanoic acid. Based on this finding, as well as on the NMR and mass spectrometry data, we suggested that LCB of ceramide **2** has 23 carbon atoms and an *iso-*type of unsaturated polymethylene chain. Accordingly, the structure of **2** was determined to be (2*S*,3*S*,4*R*,9*Z*)-2-[(2*R*)-2-hydroxyhexadecanoylamino]-21-methyl-9-docosen-1,3,4-triol.

Compound **3** was characterized from a mixture with compound **2** at a ratio of 2:1 on the basis of the evaluation of the ion peak intensities in ESI mass-spectra. The IR spectrum of compound **3** showed the presence of hydroxyl (3407 cm^−1^) and amide (1655, 1522 cm^−1^) groups. The positive HRESI mass spectrum of this mixture showed two [M + Na]^+^ ion peaks at *m*/*z* 662.5324 corresponding to compound **2** and at *m*/*z* 676.5463 corresponding to compound **3**. Therefore, the molecular formula of compound **3** was determined as C_40_H_79_NO_5_ from the [M + Na]^+^ sodium adduct ion peak at *m*/*z* 676.5463 in the (+)HRESIMS and the [M – H]^−^ deprotonated molecular ion peak at *m*/*z* 652.5520 in the (–)HRESIMS (Figures S16 and S17). The NMR spectra of compounds **3** and **2** were almost identical (Figures S18–S22), but the MWs of **3** and **2** differed by 14 amu. A GC–MS analysis and mass spectra of fatty acid methyl esters obtained from the mixture of **3** and **2** showed the presence of FAME-2 containing saturated methyl 2-hydroxyhexadecanoate of the normal type and FAME-3 containing methyl 2-hydroxyheptadecanoate of the normal type. Thus, compounds **2** and **3** differed from each other by FA residues, C_16_ in **2** and C_17_ in **3,** and had an identical C_23_ unsaturated phytosphingosine-type LCB. Thus, the structure of **3** was determined to be (2*S*,3*S*,4*R*,9*Z*)-2-[(2*R*)-2-hydroxyheptadecanoylamino]-21-methyl-9-docosen-1,3,4-triol.

The IR spectrum of compound **4** showed the presence of hydroxyl (3383 cm^−1^) and amide (1649, 1538 cm^−1^) groups. The molecular formula of compound **4** was determined as C_45_H_85_NO_9_ from the [M + Na]^+^ sodium adduct ion peak at *m*/*z* 806.6110 in the (+)HRESIMS and the [M – H]^−^ deprotonated molecular ion peak at *m*/*z* 782.6154 in the (–)HRESIMS (Figures S23 and S24). The ^1^H- and ^13^C-NMR spectroscopic data of the ceramide moiety of **4** showed the resonances of protons and carbons of three terminal methyls CH_3_-21, CH_3_-22, and CH_3_-17′ [*δ*_H_ 0.87 t (7.3), 0.87 d (6.2), 0.89 t (7.0); *δ*_C_ 11.3, 19.1, 14.0], three oxygenated groups CH_2_-1 [*δ*_Ha_ 4.70 dd (10.6, 5.6), *δ*_Hb_ 4.26 dd (10.6, 4.1); *δ*_C_ 69.9], CH-3 (*δ*_H_ 4.77 m; *δ*_C_ 72.1), and CH-2′ (*δ*_H_ 4.58 m; *δ*_C_ 72.3), one amide group NH-CO [*δ*_H_ 8.32 d (9.0); *δ*_C_ 175.4], the 4(5)-double bond [*δ*_H_ 5.99 dd (15.8, 6.2), 5.93 dt (15.8, 6.2); *δ*_C_ 131.7, 132.2], the 9(10)-double bond (*δ*_H_ 5.50 m, 5.49 m; *δ*_C_ 130.3, 129.6), and one characteristic methine CH-2 attached to nitrogen atom [*δ*_H_ 4.81 m; *δ*_C_ 54.4] (Table 2, Figures S25 and S26). Thus, the presence of characteristic signals of an unsaturated sphingosine-type ceramide including the 2-hydroxy FA residue in the ^1^H- and ^13^C-NMR spectra of the ceramide part of **4** was shown (Figure 1). Moreover, the ceramide moiety of **4** had normal and *anteiso-*types of side chains because the carbon atom signals of the terminal methyl groups were observed at *δ*_C_ 14.0 (normal form) and 11.3 and 19.1 (*anteiso-*form) in the ^13^C-NMR spectrum (Table 2). The ^1^H-^1^H COSY and HSQC correlations in the NMR spectra of **4** indicated the corresponding sequences of protons at C-1 to C-11; C-21 to C-22 through C-19 and C-20; C-2 to NH; C-2′ to C-4′, and C-16′ to C-14′ (Table 2, Figure 2B, Figures S27 and S28). The key HMBC cross-peaks such as Hb-1/C-2, C-3; H-2/C-1′; H-3/C-2, C-4; H-4/C-5, C-6; H-5/C-6, C-7; H-8/C-6, C-7, C-10; H-9/C-8, C-10, C-11; H-11/C-9; H_3_-21/C-19, C-20; H_3_-22/C-19; NH/C-2, C-1′; H-2′/C-1′; Ha-3′/C-1′, C-2′, C-4′; H_2_-16′/C-15′; and H_3_-17′/C-15′, C-16′ confirmed the overall structure of the ceramide part of **4** (Figure 2B and Figure S29).

A GC–MS analysis of FAME-4 showed the existence of one component belonging to saturated methyl 2-hydroxyheptadecanoate of the normal type. Based on this finding, as well as on the NMR and mass spectrometry data, we assumed that the LCB of the ceramide part of **4** has 22 carbon atoms and an *anteiso-*type of unsaturated polymethylene chain. The *E-*configuration of the 4(5)-double bond in LCB was determined on the basis of the coupling constant between H-4 and H-5 (15.8 Hz) in the ^1^H-NMR spectrum of **4** (Table 2). The geometry of the 9(10)-double bond in LCB was characterized as *Z* on the basis of the ^13^C-NMR chemical shifts of methylene carbons at *δ*_C_ 27.2 (C-8) and *δ*_C_ 27.0 (C-11) [19]. The location of the double bond in the LCB moiety was determined through ^1^H-^1^H COSY and HMBC NMR experiments (Table 2, Figure 2B, Figures S27 and S29).

The absolute configuration of C-2 and C-3 in LCB of the ceramide part of **4** is suggested to be (2*S*,3*R*) according to the similarities of the ^1^H-NMR data with the previously known asteriacerebroside G with a (2*S*,3*R*)-configuration of asymmetric centers [18].

In addition to the above-mentioned signals, the ^1^H-NMR spectrum of **4** exhibited one resonance in the de-shielded region due to the anomeric proton of the monosaccharide unit at *δ*_H_ 4.92 that correlated in the HSQC experiment with a carbon signal at *δ*_C_ 105.4 (Table 2). The ^1^H-^1^H COSY, HSQC, HMBC, and ROESY experiments led to the assignment of all the proton and carbon signals to the carbohydrate residue of **4** (Table 2, Figure 2B, Figures S25–S29). The coupling constant (7.8 Hz) of the anomeric proton was indicative of a β-configuration of the glycosidic bond. The NMR spectroscopic data of the monosaccharide moiety strictly coincided with those of a β-glucopyranosyl residue of the known asteriacerebroside G from *A. amurensis* [18]. The attachment of the monosaccharide to the ceramide part of **4** was deduced from the long-range correlations in the HMBC spectrum. There were cross-peaks between H-1″ of Glc*p* and C-1 of aglycon, as well as between H-1 of the ceramide part and C-1″ of Glc*p* (Figure 2B). Acid hydrolysis of cerebroside **4** with 2M TFA was carried out to confirm the identification of its monosaccharide unit as glucose. An alcoholysis of sugar by ©-(−)-2-octanol followed by acetylation, a GC analysis, and a comparison with the corresponding derivatives of standard monosaccharides allowed us to identify the D-configuration for the β-glucopyranosyl residue of **4**.

**Figure 3:**
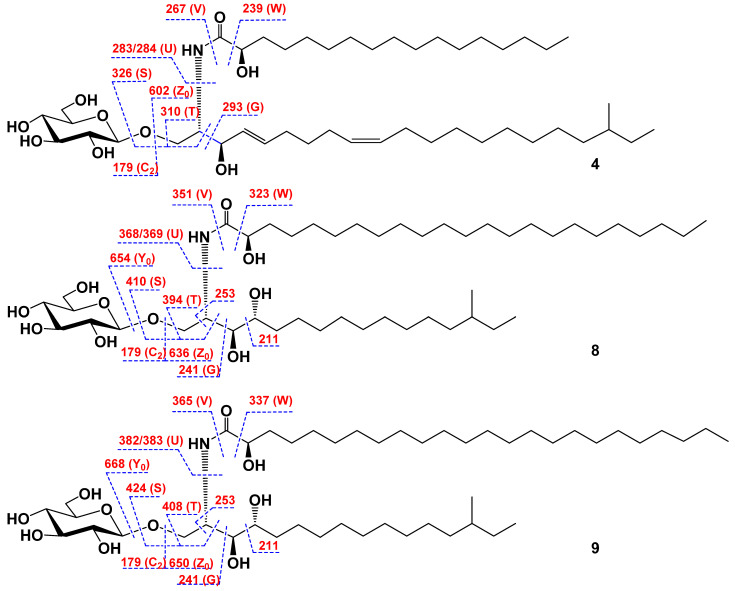
The key fragmentary peaks in (–)ESIMS/MS spectra of compounds **4**, **8**, and **9**.

The presence of a monosaccharide unit and the structure of the ceramide part of cerebroside **4** were confirmed by ESIMS/MS data. In fact, the (−)ESIMS/MS spectrum of the molecular anion peak –M − H]^−^ at *m*/*z* 782 showed fragmentary peaks obtained through the loss of a sugar unit at *m*/*z* 602 [–M − H)−180]^−^ (Z_0_-ion) and 179 [hexo–e − H]^−^ (C_2_-ion), and also a few characteristic fragmentary peaks due to the cleavage of the ceramide part of **4**: *m*/*z* 326 [–M − H)−456]^−^, the loss of a monosaccharide residue, and the cleavage of the bond between C-2 and C-3 (S-ion); *m*/*z* 310 [–M − H)−472]^−^, the loss of monosaccharide residue, and the cleavage of the bond between C-2 and C-3 (T-ion); *m*/*z* 293 [–M − H)−489]^−^, the cleavage of the bond between C-2 and C-3 (G-ion); *m*/*z* 283/284 [–M − H)−499/500]^−^, the cleavage of the bond between C-2 and NH (U-ion); *m*/*z* 267 [–M − H)−515]^−^, the cleavage of the amide bond (V-ion), and *m*/*z* 239 [–M − H)−543]^−^, and the cleavage of the bond between C-1′ and C-2′ (W-ion) (Figure 3).

On the basis of all the above-mentioned data, we determined the structure of cerebroside **4** to be (2*S*,3*R*,4*E*,9*Z*)-1-*O*-(β-D-glucopyranosyl)-2-[(2*R*)-2-hydroxyheptadecanoylamino]-19-methyl-4,9-henicosadien-3-ol.

After carrying out an extensive 2D NMR and MS analysis of cerebrosides **4**, **8**, and **9**, we suggested that the monosaccharide moiety of **4** is identical to those of glycosides **8** and **9**.

The IR spectrum of compound **8** showed the presence of hydroxyl (3401 cm^−1^) and amide (1634, 1541 cm^−1^) groups. The molecular formula of compound **8** was determined as C_46_H_91_NO_10_ from the [M + Na]^+^ sodium adduct ion peak at *m*/*z* 840.6336 in the (+)HRESIMS and the [M – H]^−^ deprotonated molecular ion peak at *m*/*z* 816.6574 in the (–)HRESIMS (Figures S30 and S31). The ^1^H- and ^13^C-NMR spectroscopic data of the ceramide part of **8** showed the resonances of protons and carbons of three terminal methyls CH_3_-16, CH_3_-17, and CH_3_-22′ [*δ*_H_ 0.86 t (7.5), 0.86 d (6.4), 0.88 t (7.0); *δ*_C_ 11.3, 19.2, 14.0], four oxygenated groups CH_2_-1 [*δ*_Ha_ 4.71 dd (10.6, 6.5), *δ*_Hb_ 4.53 dd (10.6, 4.4); *δ*_C_ 70.3], CH-3 [*δ*_H_ 4.32 dd (12.3, 5.5); *δ*_C_ 75.7], CH-4 (*δ*_H_ 4.21 m; *δ*_C_ 72.4), and CH-2′ (*δ*_H_ 4.58 m; *δ*_C_ 72.3), one amide group NH-CO [*δ*_H_ 8.54 d (9.2); *δ*_C_ 175.4], and one characteristic methine CH-2 [*δ*_H_ 5.26 m; *δ*_C_ 51.5] attached at the nitrogen atom (Table 2, Figures S32 and S33). Thus, the ^1^H- and ^13^C-NMR spectra of **8** exhibited the characteristic signals of a saturated phytosphingosine-type ceramide residue containing a 2-hydroxy FA (Figure 1). Moreover, the ceramide part of **8** had the normal and *anteiso-*types of side chains because the carbon atom signals of the terminal methyl groups were observed at *δ*_C_ 14.0 (normal form) and 11.3 and 19.2 (*anteiso-*form) in the ^13^C-NMR spectrum (Table 2). The ^1^H-^1^H COSY and HSQC correlations in the NMR spectra of **8** indicated the corresponding sequences of protons at C-1 to C-5; C-16 to C-17 through C-15 and C-14; C-2 to NH; C-2′ to C-4′, and C-22′ to C-20′ (Table 2, Figure 2C, Figures S34 and S35). The key HMBC cross-peaks such as Hb-1/C-2, C-3; H-2/C-1′; H-3/C-4; H-4/C-5; H_3_-16/C-14, C-15; H_3_-17/C-14; NH/C-2, C-1′; H-2′/C-1′; Ha-3′/C-1′, C-2′, C-4′; H_2_-21′/C-20′; H_3_-22′/C-21′ confirmed the common structure of the ceramide part of **8** (Figure 2C and Figure S36).

A GC–MS analysis of FAME-5 showed the existence of one component that belonged to a saturated methyl 2-hydroxytricosanoate of the normal type. Based on this finding, as well as on the NMR and mass spectrometric data, we assumed that the LCB of the ceramide part of **8** has 17 carbon atoms and the *anteiso-*type of saturated polymethylene chain. The absolute configuration of LCB of the ceramide moiety of **8** is suggested to be *D-ribo-*(2*S*,3*S*,4*R*) on the basis of the similarity of its ^1^H-NMR spectroscopic data with those of the LCB of ceramide **1**. The attachment of the monosaccharide to the ceramide part of **8** was deduced from long-range correlations in the HMBC spectrum. There were cross-peaks between H-1″ of Glc*p* and C-1 of aglycon, as well as between H-1 of the ceramide part and C-1″ of Glc*p* (Figure 2C and Figure S36).

The presence of a monosaccharide unit and the structure of the ceramide part of cerebroside **8** were confirmed by ESIMS/MS data. In fact, the (−)ESIMS/MS spectrum of the molecular anion peak –M − H]^−^ at *m*/*z* 816 showed fragmentary peaks obtained through the loss of a sugar unit at *m*/*z* 654 [–M − H)−162]^−^ (Y_0_-ion), *m*/*z* 636 [–M − H)−180]^−^ (Z_0_-ion) and 179 [hexo–e − H]^−^ (C_2_-ion), and also few characteristic fragmentary peaks due to the cleavage of the ceramide part of **8**: *m*/*z* 410 [–M − H)−406]^−^, the loss of a monosaccharide residue and the cleavage of the bond between C-2 and C-3 (S-ion); *m*/*z* 394 [–M − H)−422]^−^, the loss of a monosaccharide residue and the cleavage of the bond between C-2 and C-3 (T-ion); *m*/*z* 368/369 [–M – H)−448/449]^−^, the cleavage of the bond between C-2 and NH (U-ion); *m*/*z* 351 [–M – H)−465]^−^, the cleavage of the amide bond (V-ion); *m*/*z* 323 [–M – H)−493]^−^, the cleavage of the bond between C-1′ and C-2′ (W-ion); *m*/*z* 253 [–M – H)−563]^−^, the cleavage of the bonds between C-1 and C-2 and simultaneously between C-2 and NH; *m*/*z* 241 [–M – H)−575]^−^, the cleavage of the bond between C-2 and C-3 (G-ion); and 211 [–M – H)−605]^−^, the cleavage of the bond between C-3 and C-4 (Figure 3).

Hence, we determined the structure of cerebroside **8** to be (2*S*,3*S*,4*R*)-1-*O*-(β-D-glucopyranosyl)-2-[(2*R*)-2-hydroxytricosanoylamino]-14-methylhexadecan-3,4-diol.

The IR spectrum of compound **9** showed the presence of hydroxyl (3401 cm^−1^) and amide (1626, 1540 cm^−1^) groups. The molecular formula of compound **9** was determined as C_47_H_93_NO_10_ from the [M + Na]^+^ sodium adduct ion peak at *m*/*z* 854.6687 in the (+)HRESIMS and the [M – H]^−^ deprotonated molecular ion peak at *m*/*z* 830.6730 in the (–)HRESIMS (Figures S37 and S38). Based on a thorough 2D NMR analysis of cerebrosides **9** and **8**, we suggested that the ceramide part of **9** is almost identical to those of compound **8** (Figure S39–S43). However, a comparison of the molecular weights of **8** and **9** showed that they differ in MW by 14 amu. A GC–MS analysis of FAME-6 showed the presence of one component that was characterized as saturated methyl 2-hydroxytetracosanoate normal type.

The presence of a monosaccharide unit and the structure of the ceramide part of cerebroside **9** were confirmed by ESIMS/MS data. In fact, the (−)ESIMS/MS spectrum of the molecular anion peak –M – H]^−^ at *m*/*z* 830 showed fragmentary peaks obtained through the loss of a sugar unit at *m*/*z* 668 [–M – H)−162]^−^ (Y_0_-ion), *m*/*z* 650 [–M – H)−180]^−^ (Z_0_-ion), and 179 [hexo–e – H]^−^ (C_2_-ion) and also few characteristic fragmentary peaks due to the cleavage of the ceramide part of **9**: *m*/*z* 424 [–M – H)−406]^−^, the loss of a monosaccharide residue and the cleavage of the bond between C-2 and C-3 (S-ion); *m*/*z* 408 [–M – H)−422]^−^, the loss of a monosaccharide residue and the cleavage of the bond between C-2 and C-3 (T-ion); *m*/*z* 382/383 [–M – H)−448/449]^−^, the cleavage of the bond between C-2 and NH (U-ion); *m*/*z* 365 [–M – H)−465]^−^, the cleavage of the amide bond (V-ion); *m*/*z* 337 [–M – H)−493]^−^, the cleavage of the bond between C-1′ and C-2′ (W-ion); *m*/*z* 253 [–M – H)−577]^−^, the cleavage of the bonds between C-1 and C-2 and simultaneously between C-2 and NH; *m*/*z* 241 [–M – H)−589]^−^, the cleavage of the bond between C-2 and C-3 (G-ion); and 211 [–M – H)−619]^−^, and the cleavage of the bond between C-3 and C-4 (Figure 3).

Thus, we determined the structure of cerebroside **9** to be (2*S*,3*S*,4*R*)-1-*O*-(β-D-glucopyranosyl)-2-[(2*R*)-2-hydroxytetracosanoylamino]-14-methyl-hexadecan-3,4-diol.

### 2.2. The Cytotoxic Activity of Compounds ***1**–**3***, ***5**–**7***, and ***9*** against Normal and Cancer Cells and their Effect on Colony Formation and Growth of Human Cancer Cells

In the present study, the cytotoxic activity of compounds **1**–**3**, **5**–**7**, and **9** against human normal embryonic kidney cells HEK293 and a panel of human cancer cells HT-29, SK-MEL-28, and MDA-MB-231 was measured by the MTS assay after 24 h of exposure. Different concentrations of doxorubicin (10, 50, and 100 µM), used as a positive control, and compounds **1**–**3**, **5**–**7**, and **9** (1, 10, and 50 µM) were studied. As a result, it was found that compounds **1**–**3**, **5**–**7**, and **9** had moderate cytotoxic activity against HEK293, HT-29, and SK-MEL-28 (Table 3). These compounds slightly inhibited the viability of HEK293, HT-29, and SK-MEL-28 cells at concentrations of up to 50 µM (with a percentage of inhibition lower than 15%). Compounds **1**, **7**, and **9** also possessed slight cytotoxic activity against MDA-MB-231 cells at concentrations of up to 50 µM. The half maximal inhibitory concentration (IC_50_) of compounds **2**, **3**, **5**, and **6** that caused inhibition of 50% cell viability was recorded only for breast carcinoma cells MDA-MB-231 and was comparable among the compounds under study (Table 3). The IC_50_ of doxorubicin (Doxo) was 35.7 µM, 21.8 µM, 40.0 µM, and 22.3 µM for HEK293, HT-29, SK-MEL-28, and MDA-MB-231 cell lines, respectively (Table 3).

Since compounds **1**–**3**, **5**–**7**, and **9** inhibited the viability of breast cancer cells MDA-MB-231, we then tested their ability to inhibit colony formation of MDA-MB-231 cells using the soft agar assay. The colony formation assay, also referred to as soft agar assay, allows for screening the therapeutic efficacy of compounds for anchorage-independent cell growth, which is one of the hallmark characteristics of cellular transformation and uncontrolled growth of cancer cells [26].

**Figure 4:**
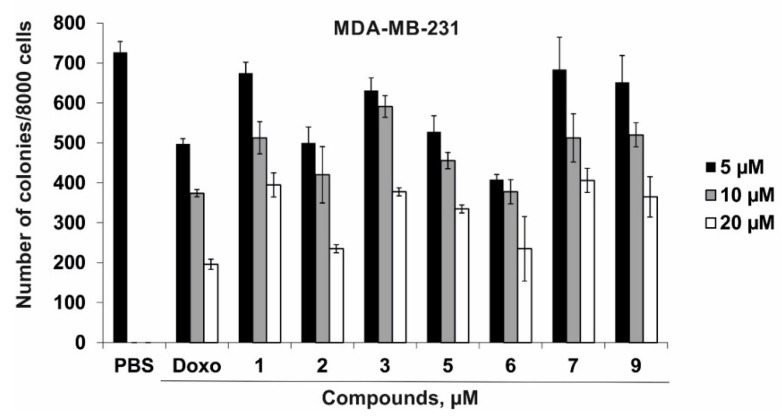
The effect of compounds **1**–**3**, **5**–**7**, and **9** on colony formation of human breast cancer cells MDA-MB-231 (2.4 × 10^4^) that were exposed to PBS (control), Doxo (5, 10, and 20 µM), or the compounds under study (5, 10, and 20 µM) and placed on dishes with 0.3% Basal Medium Eagle (BME) agar containing 10% fetal bovine serum FBS, 2 mM L-glutamine, and 25 µg/mL gentamicin. After 14 days of incubation, the number of colonies was counted under a microscope using the ImageJ software program.

As a result, we found that compounds **1**, **3**, **7,** and **9** at a concentration of 20 µM had a comparable effect on MDA-MB-231 colony formation and decreased the number of colonies by 46%, 48%, 44%, and 50%, respectively. Compounds **2**, **5**, and **6** (20 µM) inhibited colony formation of MDA-MB-231 cells by 68%, 54%, and 68%, respectively. The colony-inhibiting activity of compounds **2**, **5**, and **6** (20 µM) was comparable with the effect of doxorubicin that reduced the number of colonies by 70% at a concentration of 20 µM (Figure 4).

## 3. Materials and Methods

### 3.1. General Procedures

Optical rotations were determined on a PerkinElmer 343 polarimeter (Waltham, MA, USA). UV spectra were recorded on a Shimadzu UV-1601 PC spectrophotometer (Shimadzu, Kyoto, Japan). IR spectra were recorded using a Bruker Equinox 55 spectrophotometer in CDCl_3_ (Bruker, Göttingen, Germany). The ^1^H- and ^13^C-NMR spectra were obtained on Bruker Avance III 700 spectrometer (Bruker BioSpin, Bremen, Germany) at 700.13 and 176.04 MHz, respectively; chemical shifts were referenced to the corresponding residual solvent signals (*δ***_H_** 7.21/*δ***_C_** 123.5 for C_5_D_5_N). The HRESIMS spectra were recorded on a Bruker Impact II Q-TOF mass spectrometer (Bruker, Bremen, Germany); the samples were dissolved in MeOH (c 0.001 mg/mL). HPLC separations were carried out on an Agilent 1100 Series chromatograph (Agilent Technologies, Santa Clara, CA, USA) equipped with a differential refractometer and with the following columns used: Diasfer-110-C18 (10 μm, 250 × 15 mm, Biochemmack, Moscow, Russia), Discovery HS C18-10 (10 μm, 250 × 21.2 mm, Supelco, North Harrison, PA, USA), and Discovery C18 (5 μm, 250 × 4 mm, Supelco, North Harrison, PA, USA). GC and GC–MS analyses were performed on a GC 2010 chromatograph equipped with a flame ionization detector and a gas chromatograph–mass spectrometer GCMS-QP5050, both Shimadzu (Kioto, Japan), and with fused silica capillary columns Supelcowax 10 and MDN-5S (both columns 30 m, 0.25 mm ID, 0.25 lm film, Supelco, USA). Low-pressure liquid column chromatography was carried out using Polychrom-1 (powdered Teflon, 0.25–0.50 mm; Biolar, Olaine, Latvia), Si gel KSK (50–160 μm, Sorbpolimer, Krasnodar, Russia), and Florisil (60–100 μm, Sigma Aldrich, St. Louis, MO, USA). Sorbfil Si gel plates (4.5 × 6.0 cm, 5–17 μm, Sorbpolimer, Krasnodar, Russia) were used for thin-layer chromatography.

### 3.2. Animal Material

Specimens of *Ceramaster patagonicus* Sladen, 1889 (order Valvatida, family Goniasteridae) were collected at a depth of 150–300 m in the Sea of Okhotsk, off Iturup Island, during the 42nd research cruise aboard the R/V *Akademik Oparin* in August 2012. The species was identified by B.B. Grebnev (G.B. Elyakov Pacific Institute of Bioorganic Chemistry FEB RAS, Vladivostok, Russia). A voucher specimen [no. 042-67] is deposited at the marine specimen collection of the G.B. Elyakov Pacific Institute of Bioorganic Chemistry FEB RAS, Vladivostok, Russia.

### 3.3. Extraction and Isolation

The fresh *C. patagonicus* specimens (3 kg wet weight) were cut into small pieces and extracted with CHCl_3_:MeOH (2:1) followed by further extraction with CHCl_3_:MeOH (1:1) and EtOH. The combined extracts were concentrated in vacuo to a residue of 159.5 g. This residue was separated between H_2_O (1.5 L) and AcOEt:BuOH (2:1) (4.5 L), and the organic layer was concentrated *in vacuo* to obtain a less polar fraction (51.5 g), which was washed with cold acetone (1 L). The acetone-soluble fraction (28.5 g) was chromatographed on a Si gel column (19 × 4.5 cm) using CHCl_3_, CHCl_3_:MeOH (97:3), and CHCl_3_:MeOH (9:1) to yield four fractions: 1 (932 mg), 2 (486 mg), 3 (735 mg), and 4 (1.04 g). Fractions 1–4 were further chromatographed on a Si gel column (10 × 4 cm) using *n*-hexane:AcOEt:MeOH (stepwise gradient, 6:3:0.1→6:3:0.7, v/v/v) to yield six subfractions: 21 (123 mg), 31 (475 mg), 32 (231 mg), 41 (55 mg), 42 (212 mg), and 43 (570 mg), which were then analyzed by TLC in the eluent system CHCl_3_:MeOH:H_2_O (8:1:0.1, v/v/v). Subfractions 21–43 mainly contained ceramides, cerebrosides, admixtures of pigments, and other concomitant lipids. A HPLC separation of subfraction 31 (475 mg) on a Diasfer-110-C18 column (2.5 mL/min) with MeOH as an eluent yielded pure **2** (12.0 mg, *R*_t_ 68.2 min) and seventeen subfractions 31-3–31-15 and 31-17–31-20. A HPLC separation of subfractions 31-14 and 31-18 on a Discovery C18 column (2.5 mL/min) with MeOH as an eluent yielded pure **1** (2.0 mg, *R*_t_ 15.1 min) and unseparated mixture of **3** and **2** (1.5 mg, *R*_t_ 17.8 min). HPLC separation of subfraction 42 (212 mg) on a Discovery HS C18-10 column (4.0 mL/min) with MeOH as an eluent yielded pure **5** (3.0 mg, *R*_t_ 133.2 min), **6** (4.5 mg, *R*_t_ 153.2 min), and thirteen subfractions 42-4–42-15 and 42-17. A HPLC separation of subfractions 42-12, 42-13, 42-15, and 42-17 on a Discovery C18 column (2.5 mL/min) with MeOH as an eluent yielded pure **4** (1.0 mg, *R*_t_ 15.9 min), **7** (2.5 mg, *R*_t_ 18.9 min), **8** (0.5 mg, *R*_t_ 21.7 min), and **9** (1.5 mg, *R*_t_ 26.9 min).

### 3.4. Compounds Characterization Data

*(2S,3S,4R,9Z)-2-[(2R)-2-hydroxyhexadecanoylamino]-19-methyl-9-icosen-1,3,4-triol* (**1**): Amorphous powder; [α]_D_^25^: +15.4 (*c* 0.1, MeOH); IR (CDCl_3_) *ν*_max_ 3402, 2934, 2920, 2855, 1722, 1656, 1625, 1523, 1493, 1462, 1365, 1274, 1186, 1130, 1080 cm^−1^; (+)HRESIMS *m*/*z* 634.5376 [M + Na]^+^ (calcd for [C_37_H_73_NO_5_Na]^+^, 634.5381); (–)HRESIMS *m*/*z* 610.5412 [M – H]^−^ (calcd for [C_37_H_72_NO_5_]^−^, 610.5416); ^1^H- and ^13^C-NMR data, see Table 1.

*(2S,3S,4R,9Z)-2-[(2R)-2-hydroxyhexadecanoylamino]-21-methyl-9-docosen-1,3,4-triol* (**2**): Amorphous powder; [α]_D_^25^: +10.8 (*c* 0.1, MeOH); IR (CDCl_3_) *ν*_max_ 3404, 2937, 2922, 2853, 1724, 1657, 1625, 1522, 1495, 1460, 1365, 1275, 1187, 1131, 1080 cm^−1^; (+)HRESIMS *m*/*z* 662.5324 [M + Na]^+^ (calcd for [C_39_H_77_NO_5_Na]^+^, 662.5694); (–)HRESIMS *m*/*z* 638.5364 [M – H]^−^ (calcd for [C_39_H_76_NO_5_]^−^, 638.5729); ^1^H- and ^13^C-NMR data, see Table 1.

*(2S,3S,4R,9Z)-2-[(2R)-2-hydroxyheptadecanoylamino]-21-methyl-9-docosen-1,3,4-triol* (**3**) as mixed with **2** (ratio 2:1): Amorphous powder; [α]_D_^25^: +10.0 (*c* 0.1, MeOH); IR (CDCl_3_) *ν*_max_ 3407, 2932, 2927, 2855, 1728, 1655, 1624, 1522, 1495, 1464, 1366, 1278, 1183, 1130, 1081 cm^−1^; (+)HRESIMS *m*/*z* 676.5463 [M + Na]^+^ (calcd for [C_40_H_79_NO_5_Na]^+^, 676.5850); (–)HRESIMS *m*/*z* 652.5520 [M – H]^−^ (calcd for [C_40_H_78_NO_5_]^−^, 652.5885); ^1^H- and ^13^C-NMR data, see Table 1.

*(2S,3R,4E,9Z)-1-O-(β-D-glucopyranosyl)-2-[(2R)-2-hydroxyheptadecanoylamino]-19-methyl-4,9-henicosadien-3-ol* (**4**): Amorphous powder; [α]_D_^25^: –9.7 (*c* 0.05, MeOH); IR (CDCl_3_) *ν*_max_ 3383, 2927, 2854, 1718, 1649, 1602, 1538, 1461, 1366, 1261, 1098, 1078, 1034 cm^−1^; (+)HRESIMS *m*/*z* 806.6110 [M + Na]^+^ (calcd for C_45_H_85_NO_9_Na, 806.6117); (–)HRESIMS *m*/*z* 782.6154 [M – H]^−^ (calcd for C_45_H_84_NO_9_, 782.6152); (–)ESIMS/MS of the ion at *m*/*z* 782: *m*/*z* 602 [–M − H)−180]^−^, 326 [–M − H)−456]^−^, 310 [–M − H)−472]^−^, 293 [–M − H)−489]^−^, 283/284 [–M − H)−499/500]^−^, 267 [–M − H)−515]^−^, 239 [–M − H)−543]^−^, 179 [hexo–e − H]^−^; ^1^H- and ^13^C-NMR data, see Table 2.

*(2S,3S,4R)-1-O-(β-D-glucopyranosyl)-2-[(2R)-2-hydroxytricosanoylamino]-14-methylhexadecan-3,4-diol* (**8**): Amorphous powder; [α]_D_^25^: –14.0 (*c* 0.1, MeOH); IR (CDCl_3_) *ν*_max_ 3401, 2925, 2854, 1732, 1634, 1602, 1541, 1457, 1363, 1261, 1078, 1018 cm^−1^; (+)HRESIMS *m*/*z* 840.6336 [M + Na]^+^ (calcd for C_46_H_91_NO_10_Na, 840.6335); (–)HRESIMS *m*/*z* 816.6574 [M – H]^−^ (calcd for C_46_H_90_NO_10_, 816.6570); (–)ESIMS/MS of the ion at *m*/*z* 816: *m*/*z* 654 [–M − H)−162]^−^, 636 [–M − H)−180]^−^, 410 [–M − H)−406]^−^, 394 [–M − H)−422]^−^, 368/369 [–M − H)−448/449]^−^, 351 [–M − H)−465]^−^, 323 [–M − H)−493]^−^, 253 [–M − H)−563]^−^, 241 [–M − H)−575]^−^, 211 [–M − H)−605]^−^, 179 [hexo–e − H]^−^; ^1^H- and ^13^C-NMR data, see Table 2.

*(2S,3S,4R)-1-O-(β-D-glucopyranosyl)-2-[(2R)-2-hydroxytetracosanoylamino]-14-methylhexadecan-3,4-diol* (**9**): Amorphous powder; [α]_D_^25^: –17.5 (*c* 0.1, MeOH); IR (CDCl_3_) *ν*_max_ 3401, 2925, 2854, 1731, 1626, 1602, 1540, 1455, 1297, 1103, 1078, 1029 cm^−1^; (+)HRESIMS *m*/*z* 854.6687 [M + Na]^+^ (calcd for C_47_H_93_NO_10_Na, 854.6692); (–)HRESIMS *m*/*z* 830.6730 [M – H]^−^ (calcd for C_47_H_92_NO_10_, 830.6727); (–)ESIMS/MS of the ion at *m*/*z* 830: *m*/*z* 668 [–M − H)−162]^−^, 650 [–M − H)−180]^−^, 424 [–M − H)−406]^−^, 408 [–M − H)−422]^−^, 382/383 [–M − H)−448/449]^−^, 365 [–M − H)−465]^−^, 337 [–M − H)−493]^−^, 253 [–M − H)−577]^−^, 241 [–M − H)−589]^−^, 211 [–M − H)−619]^−^, 179 [hexo–e − H]^−^; ^1^H- and ^13^C-NMR data, see Table 2.

### 3.5. Methanolysis of Compounds ***1**–**9*** and Analysis of FAMEs

Compounds **1**–**9** (1 mg) were heated with 1 N HCl in 80% aqus. MeOH (1.0 mL) at 80°C for 4 h. The reaction mixtures were then extracted with *n*-hexane and the extracts were concentrated in vacuo to yield FAME-1–FAME-9. The FAMEs were analyzed on Supelcowax 10 columns at 200 °C. Helium was used as the carrier gas at a linear velocity of 30 cm/s. Mass spectra were recorded at 70 eV. The obtained mass spectra were compared with the NIST library and a FA mass spectra archive accessible online.

### 3.6. Acid Hydrolysis and Determination of Absolute Configurations of Monosaccharides

The acid hydrolysis of **4** (0.5 mg) was carried out in a solution of 2 M trifluoroacetic acid (TFA) (1 mL) in a sealed vial on an H_2_O bath at 100 ˚C for 2 h. The H_2_O layer was washed with CHCl_3_ (3 × 1.0 mL) and concentrated in vacuo. One drop of concentrated TFA and 0.5 mL of *R*-(–)-2-octanol (Sigma Aldrich) were added to the sugar fraction, and the sealed vial was heated in a glycerol bath at 130 ˚C for 6 h. The solution was evaporated in vacuo and exposed to a mixture of pyridine/acetic anhydride (1:1, 0.5 mL) for 24 h at room temperature. The acetylated 2-octylglycosides were analyzed by GC using the corresponding authentic samples prepared by the same procedure. The following peaks were detected in the hydrolysate of **4**: D-glucose (*t*_R_ 24.24, 24.84, 25.08, and 25.38 min). The retention times of the authentic samples were as follows: D-glucose (*t*_R_ 24.23, 24.83, 25.06, and 25.37 min), L-glucose (*t*_R_ 24.39, 24.63, 24.83, and 25.06 min).

### 3.7. Bioactivity Assay

#### 3.7.1. Reagents

The McCoy’s 5A Modified Medium (McCoy’s 5A), the Dulbecco’s Modified Eagle’s Medium (DMEM), Basal Medium Eagle (BME), phosphate-buffered saline (PBS), L-glutamine, penicillin–streptomycin solution (10 000 U/mL, 10 µg/mL) and trypsin were all purchased from Sigma-Aldrich (St. Louis, MO, USA). The MTS reagent was purchased from Promega (Madison, WI, USA). Fetal bovine serum (FBS), agar and gentamicin were purchased from ThermoFisher Scientific (Waltham, Massachusetts, USA).

#### 3.7.2. Cell Lines

The human embryonic kidney cells HEK293 (ATCC^®^ CRL-1573™), the colorectal adenocarcinoma cell line HT-29 (ATCC^®^ HTB-38), the melanoma SK-MEL-28 (ATCC^®^ HTB-72™), and breast adenocarcinoma MDA-MB-231 (ATCC^®^ HTB-26™) cell lines were obtained from the American Type Culture Collection (Manassas, VA, USA).

#### 3.7.3. Cells Culture Conditions

HT 29 cells were cultured in McCoy’s 5A medium; HEK293, SK-MEL-28, and MDA-MB-231 cell lines were maintained in the DMEM medium. The culture media were supplemented with 10% FBS and a 1% penicillin/streptomycin solution. The cell cultures were maintained at 37 ˚C in a humidified atmosphere containing 5% CO_2_.

#### 3.7.4. Preparation of Compounds

Compounds **1**–**3**, **5**–**7**, and **9** were dissolved in a sterile dimethyl sulfoxide solution (DMSO) to prepare stock concentrations of 20 mM. Cells were exposed to serially diluted **1**–**3**, **5**–**7**, and **9** (1–100 µM) (with the culture medium used as a diluent) (the final concentration of DMSO was less than 0.5%).

Doxorubicin (Doxo) (Teva Pharmaceutical Industries, Ltd. (Israel)) was dissolved in sterile PBS to prepare stock concentrations of 10 mM. Cells were exposed to serially diluted Doxo (10–100 µM) (with the culture medium used as a diluent).

The vehicle control is the cells exposed to the equivalent volume of DMSO (the final concentration was less than 0.5%) for all of the experiments conducted.

#### 3.7.5. Cell Viability Assay

The effect of compounds **1**–**3**, **5**–**7**, and **9** on the viability of tested cell lines was evaluated by the MTS assay. HEK293 (1.0 × 10^4^/200 µL), HT-29 (1.0 × 10^4^/200 µL), SK-MEL-28 (0.8 × 10^4^/200 µL), and MDA-MB-231 (1.0 × 10^4^/200 µL) cells were seeded on a 96-well plate and incubated for 24 h at 37°C in a CO_2_ incubator. The cells were exposed to either DMSO (vehicle control) or Doxo at concentrations of 1, 10, 50 µM (positive control) or **1**–**3**, **5**–**7**, and **9** at concentrations of 1, 10, and 50 µM for 24 h. The cells were subsequently incubated with 15 µL MTS reagent for 3 h, and the absorbance of each well was measured at 490/630 nm on a Power Wave XS microplate reader (BioTek, Winooski, VT, USA). The concentration at which a compound exerted half of its maximal inhibitory effect on cell viability (IC_50_) was calculated using the AAT-Bioquest^®^ online calculator [27].

#### 3.7.6. Colony Formation Assay

MDA-MB-231 cells (2.4 × 10^4^/mL) were exposed to either DMSO (vehicle control) or Doxo (positive control) at concentrations of 5, 10, and 20 µM, and to **1**–**3**, **5**–**7**, and **9** at concentrations of 5, 10, 20 µM. Then, the cells were applied on dishes with 0.3% BME agar containing 10% FBS, 2 mM L-glutamine, and 25 µg/mL gentamicin. The cultures were maintained at 37°C in a 5% CO_2_ incubator for 14 days. The number and size of the colonies were estimated under a Motic microscope AE 20 and using the ImageJ software bundled with 64-bit Java 1.8.0_112 (NIH, Bethesda, Maryland, USA).

#### 3.7.7. Statistical Analysis

All assays were performed in triplicate. Results are presented as mean ± standard deviation (SD).

## 4. Conclusions

Three new ceramides (**1**–**3**) and three new cerebrosides (**4**, **8**, and **9**) along with three previously known cerebrosides—ophidiacerebrosides C (**5**), D (**6**), and CE-3-2 (**7**)—were isolated from a deep-sea starfish species, the orange cookie star *Ceramaster patagonicus.* Ceramides **1**–**3** contain *iso-*C_21_ or C_23_ Δ^9^-phytosphingosine as LCB and have C_16_ or C_17_ (2*R*)-2-hydroxy-fatty acids of the normal type. As far as we know, ceramides with the *iso-*type of LCB were isolated from this starfish for the first time. It is also worth noting that starfish-derived ceramides are the least studied class of sphingolipids. This may be due to the challenge of isolating certain components from complex mixtures of ceramides and other lipids. However, new data on the structures of starfish ceramides allow a better understanding of the biosynthetic features of these animals. Furthermore, these ceramides can also be used as chemotaxonomic markers.

Cerebroside **4** contains C_22_ Δ^9^-sphingosine of *anteiso-*type and (2*R*)-2-hydroxyheptadecanoic acid of the normal type. The C_22_ Δ^9^-sphingosine of *anteiso-*type was found in starfish cerebrosides for the first time. New compounds **8** and **9** contain saturated C-17 phytosphingosine of *anteiso-*type as LCB and differ from each other in the length of the polymethylene chain of (2*R*)-2-hydroxy-fatty acids: C_23_ at **8** and C_24_ at **9**. All the isolated cerebrosides have β-D-glucopyranose as a monosaccharide residue. It is also worth noting that all the isolated ceramides and cerebrosides have only (2*R*)-2-hydroxy-fatty acids of the normal type. As far as we know, the composition of neutral sphingolipids of the starfish *C. patagonicus* was described for the first time.

Compounds **1**–**3**, **5**–**7**, and **9** under study exhibit slight or moderate cytotoxic activity against HEK293, HT-29, SK-MEL-28, and MDA-MB-231 cells. On the other hand, compounds **1**, **3**, **7**, and **9** at a non-toxic concentration of 20 µM significantly decreased the number of colonies of MDA-MB-231 cells. The colony-inhibiting activity of compounds **2**, **5**, and **6** is comparable to the anticancer effect of doxorubicin. Ophidiacerebrosides C (**5**) and D (**6**) with (4*E*,8*E*,10*E*)-9-methylsphinga-4,8,10-trienine as LCB showed the highest cytotoxic and colony formation inhibitory effects among the cerebrosides analyzed. These data agree well with the results of published studies where these cerebrosides showed strong cytotoxic activity at a concentration of 2 μM against murine leukemic cells L1210 and moderate cytotoxic activity against a number of human cancer cell lines at concentrations of 15–34 μM [21,28]. The inhibition of cancer cell colony formation by starfish sphingolipids was shown for the first time.

## Figures and Tables

**Table 1:** ^1^H- (700.13 MHz) and ^13^C- (176.04 MHz) NMR chemical shifts of ceramides **1**−**3** in C_5_D_5_N, at 30 °C, δ in ppm, *J* values in Hz.

Position	*δ*_H_	*δ*_C_	Position	*δ*_H_	*δ*_C_
1a 1b	4.51 dt (10.9, 4.2) 4.43 m	61.8	11	2.10 q (6.7)	27.3
1-OH	6.60 m		19 or 21	1.51 m	27.9
2	5.10 sext (4.5)	52.8	20 or 22	0.88 d (6.5)	22.5
3	4.34 m	76.7	21 or 23	0.88 d (6.5)	22.5
3-OH	6.59 d (6.8)		NH	8.55 d (9.0)	
4	4.28 m	72.8	1′		175.0
4-OH	6.14 d (6.5)		2′	4.62 m	72.3
5a 5b	2.28 m 1.95 m	33.9	2′-OH	7.53 d (5.1)	
6a 6b	1.74 m 1.39 m	26.1	3′a 3′b	2.24 m 2.05 m	35.5
7a 7b	1.58 m 1.53 m	30.2	4′a 4′b	1.80 m 1.73 m	25.6
8	2.17 q (7.0)	27.5	14′ or 15′	1.25 m	31.9
9	5.50 m	130.1	15′ or 16′	1.27 m	22.7
10	5.48 m	129.9	16′ or 17′	0.89 t (7.2)	14.0

**Table 2:** ^1^H- (700.13 MHz) and ^13^C- (176.04 MHz) NMR chemical shifts of cerebrosides **4** and **8**, **9** in C_5_D_5_N, at 30 °C, δ in ppm, *J* values in Hz.

Position	4		8, 9	
	*δ*_H_	*δ*_C_	*δ*_H_	*δ*_C_
1a 1b	4.70 dd (10.6, 5.6) 4.26 dd (10.6, 4.1)	69.9	4.71 dd (10.6, 6.5) 4.53 dd (10.6, 4.4)	70.3
2	4.81 m	54.4	5.26 m	51.5
3	4.77 m	72.1	4.32 dd (12.3, 5.5)	75.7
3-OH	6.75 d (4.7)		6.69 d (6.0)	
4	5.99 dd (15.8, 6.2)	131.7	4.21 m	72.4
5a 5b	5.93 dt (15.8, 6.2)	132.2	2.24 m 1.91 m	33.9
6	2.12 m	32.1		
7	1.50 m	29.4		
8	2.11 m	27.2		
9	5.50 m	130.3		
10	5.49 m	129.6		
11	2.12 m	27.0		
19 or 14	1.30 m	34.4	1.30 m	34.5
20a or 15a 20b or 15b	1.30 m 1.12 m	36.7	1.30 m 1.10 m	36.8
21 or 16	0.87 t (7.3)	11.3	0.86 t (7.5)	11.3
22 or 17	0.87 d (6.2)	19.1	0.86 d (6.4)	19.2
NH	8.32 d (9.0)		8.54 d (9.2)	
1′		175.4		175.4
2′	4.58 m	72.3	4.58 m	72.3
2′-OH	7.53 d (5.0)		7.55 m	
3′a 3′b	2.21 m 2.02 m	35.4	2.20m 2.01 m	35.4
4′a 4′b	1.82 m 1.73 m	25.7	1.77 m 1.70 m	25.0
Terminal CH_3_	0.89 t (7.0)	14.0	0.88 t (7.0)	14.0
1″	4.92 d (7.8)	105.4	4.95 d (7.9)	105.5
2″	4.03 m	74.9	4.00 m	74.9
2″-OH	7.11 brs			
3″	4.20 m	78.2	4.17 m	78.2
4″	4.20 m	71.4	4.17 m	71.4
5″	3.91 m	78.3	3.89 m	78.3
6″a 6″b	4.51 brd (12.5) 4.35 m	62.5	4.48 brd (12.0) 4.33 m	62.5
6″-OH	6.26 brs		6.27 brs	

**Table 3:** Cytotoxic activities of compounds **1**–**3**, **5**–**7**, and **9** against normal and cancer cells.

Compounds	Half Maximal Inhibitory Concentration (IC_50_), µM			
	HEK293	HT-29	SK-MEL-28	MDA-MB-231
**Doxorubicin**	35.7 ± 1.2	21.8 ± 3.2	40.0 ± 5.0	22.3 ± 0.2
**1**	>50.0 >50.0 >50.0 >50.0 >50.0 >50.0 >50.0	>50.0 >50.0 >50.0 >50.0 >50.0 >50.0 >50.0	>50.0 >50.0 >50.0 >50.0 >50.0 >50.0 >50.0	>50.0
**2**				48.7 ± 2.4
**3**				49.4 ± 1.6
**5**				48.7 ± 1.8
**6**				40.5 ± 0.5
**7**				>50.0
**9**				>50.0

IC_50_ is the concentration of compounds that caused a 50% reduction in cell viability of tested normal and cancer cells. Values are mean ± standard deviation.

## Data Availability

The data presented in this study are available on request from the corresponding authors.

## Supplementary Materials

The following supporting information can be downloaded at: https://www.mdpi.com/article/10.3390/md20100641/s1, copies of the HRESIMS (Figures S1, S2, S9, S10, S16, S17, S23, S24, S30, S31, S37, and S38), 1H-NMR (Figures S3, S11, S18, S25, S32, and S39), 13C-NMR (Figures S4, S12, S19, S26, S33, and S40), 1H-1H-COSY (Figures S5, S13, S20, S27, S34, and S41), HSQC (Figures S6, S14, S21, S28, S35, and S42), and HMBC (Figures S7, S15, S22, S29, S36, and S43) spectra of compounds 1, 2, 3, 4, 8, and 9, respectively. This material is available free online.
