# Supplementary material for: New Ceramides and Cerebrosides from the Deep-Sea Far Eastern Starfish Ceramaster patagonicus
Source: Mar Drugs. 2022 Oct 14;20(10):641. doi: 10.3390/md20100641 (PMC9604662; doi:10.3390/md20100641)

Supplementary Materials

New Ceramides and Cerebrosides from the Deep-Sea Far Eastern Starfish *Ceramaster patagonicus*

Timofey V. Malyarenko [1,2,*], Viktor M. Zakharenko [1,2], Alla A. Kicha [1], Alexandra S. Kuzmich [1], Olesya S. Malyarenko [1], Anatoly I. Kalinovsky [1], Roman S. Popov [1], Vasily I. Svetashev [3] and Natalia V. Ivanchina [1,*]

[1] G.B. Elyakov Pacific Institute of Bioorganic Chemistry, Far Eastern Branch, Russian Academy of Sciences, Pr. 100-let Vladivostoku 159, 690022 Vladivostok, Russia

[2] Department of Bioorganic Chemistry and Biotechnology, School of Natural Sciences, Far Eastern Federal University, Russky Island, Ajax Bay 10, 690922 Vladivostok, Russia

[3] A.V. Zhirmunsky National Scientific Center of Marine Biology, Far Eastern Branch, Russian Academy of Sciences, ul. Palchevskogo 17, 690041 Vladivostok, Russia

* Correspondence: malyarenko-tv@mail.ru (T.V.M.); ivanchina@piboc.dvo.ru (N.V.I.); Tel.: +7-423-2312-360 (N.V.I.); Fax: +7-423-2314-050 (N.V.I.)

List

Figure S1. (–)-HRESIMS spectrum of ceramide **1**.

Figure S2. (+)-HRESIMS spectrum of ceramide **1**.

Figure S3. ^{1}H-NMR spectrum of ceramide **1** in C_5D_5N.

Figure S4. ^{13}C-NMR spectrum of ceramide **1** in C_5D_5N.

Figure S5. ^{1}H-^{1}H-COSY spectrum of ceramide **1** in C_5D_5N.

Figure S6. HSQC spectrum of ceramide **1** in C_5D_5N.

Figure S7. HMBC spectrum of ceramide **1** in C_5D_5N.

Figure S8. 2D TOCSY spectrum of ceramide **1** in C_5D_5N.

Figure S9. (–)-HRESIMS spectrum of ceramide **2**.

Figure S10. (+)-HRESIMS spectrum of ceramide **2.**

Figure S11. ^{1}H-NMR spectrum of ceramide **2** in C_5D_5N.

Figure S12. ^{13}C-NMR spectrum of ceramide **2** in C_5D_5N.

Figure S13. ^{1}H-^{1}H-COSY spectrum of ceramide **2** in C_5D_5N.

Figure S14. HSQC spectrum of ceramide **2** in C_5D_5N.

Figure S15. HMBC spectrum of ceramide **2** in C_5D_5N.

Figure S16. (–)-HRESIMS spectrum of ceramide **3**.

Figure S17. (+)-HRESIMS spectrum of ceramide **3**.

Figure S18. ^{1}H-NMR spectrum of ceramide **3** in C_5D_5N.

Figure S19. ^{13}C-NMR spectrum of ceramide **3** in C_5D_5N.

Figure S20. ^{1}H-^{1}H-COSY spectrum of ceramide **3** in C_5D_5N.

Figure S21. HSQC spectrum of ceramide **3** in C_5D_5N.

Figure S22. HMBC spectrum of ceramide **3** in C_5D_5N.

Figure S23. (–)-HRESIMS spectrum of cerebroside **4**.

Figure S24. (+)-HRESIMS spectrum of cerebroside **4**.

Figure S25. ^{1}H-NMR spectrum of cerebroside **4** in C_5D_5N.

Figure S26. ^{13}C-NMR spectrum of cerebroside **4** in C_5D_5N.

Figure S27. ^{1}H-^{1}H-COSY spectrum of cerebroside **4** in C_5D_5N.

Figure S28. HSQC spectrum of cerebroside **4** in C_5D_5N.

Figure S29. HMBC spectrum of cerebroside **4** in C_5D_5N.

Figure S30. (–)-HRESIMS spectrum of cerebroside **8**.

Figure S31. (+)-HRESIMS spectrum of cerebroside **8**.

Figure S32. ^{1}H-NMR spectrum of cerebroside **8** in C_5D_5N.

Figure S33. ^{13}C-NMR spectrum of cerebroside **8** in C_5D_5N.

Figure S34. ^{1}H-^{1}H-COSY spectrum of cerebroside **8** in C_5D_5N.

Figure S35. HSQC spectrum of cerebroside **8** in C_5D_5N.

Figure S36. HMBC spectrum of cerebroside **8** in C_5D_5N.

Figure S37. (–)-HRESIMS spectrum of cerebroside **9**.

Figure S38. (+)-HRESIMS spectrum of cerebroside **9**.

Figure S39. ^{1}H-NMR spectrum of cerebroside **9** in C_5D_5N.

Figure S40. ^{13}C-NMR spectrum of cerebroside **9** in C_5D_5N.

Figure S41. ^{1}H-^{1}H-COSY spectrum of cerebroside **9** in C_5D_5N.

Figure S42. HSQC spectrum of cerebroside **9** in C_5D_5N.

Figure S43. HMBC spectrum of cerebroside **9** in C_5D_5N.

Figure S1. (–)-HRESIMS spectrum of ceramide **1**.

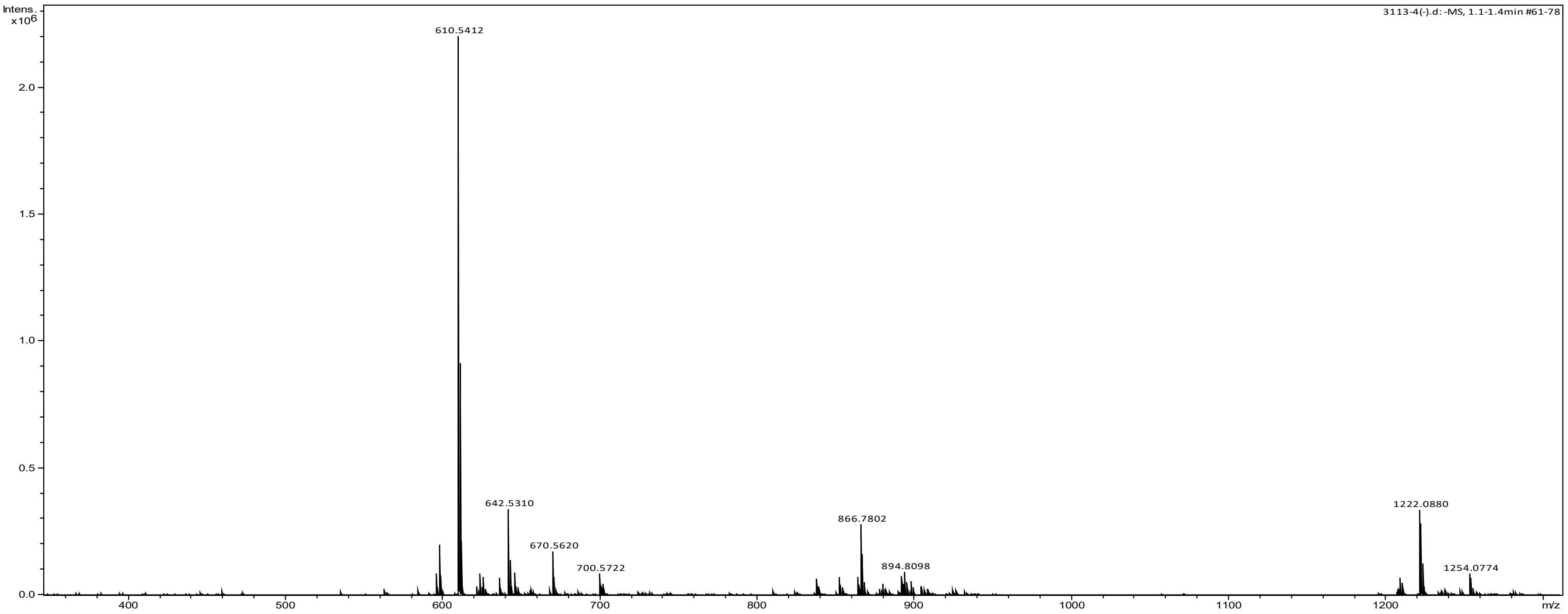

Figure S2. (+)-HRESIMS spectrum of ceramide **1**.

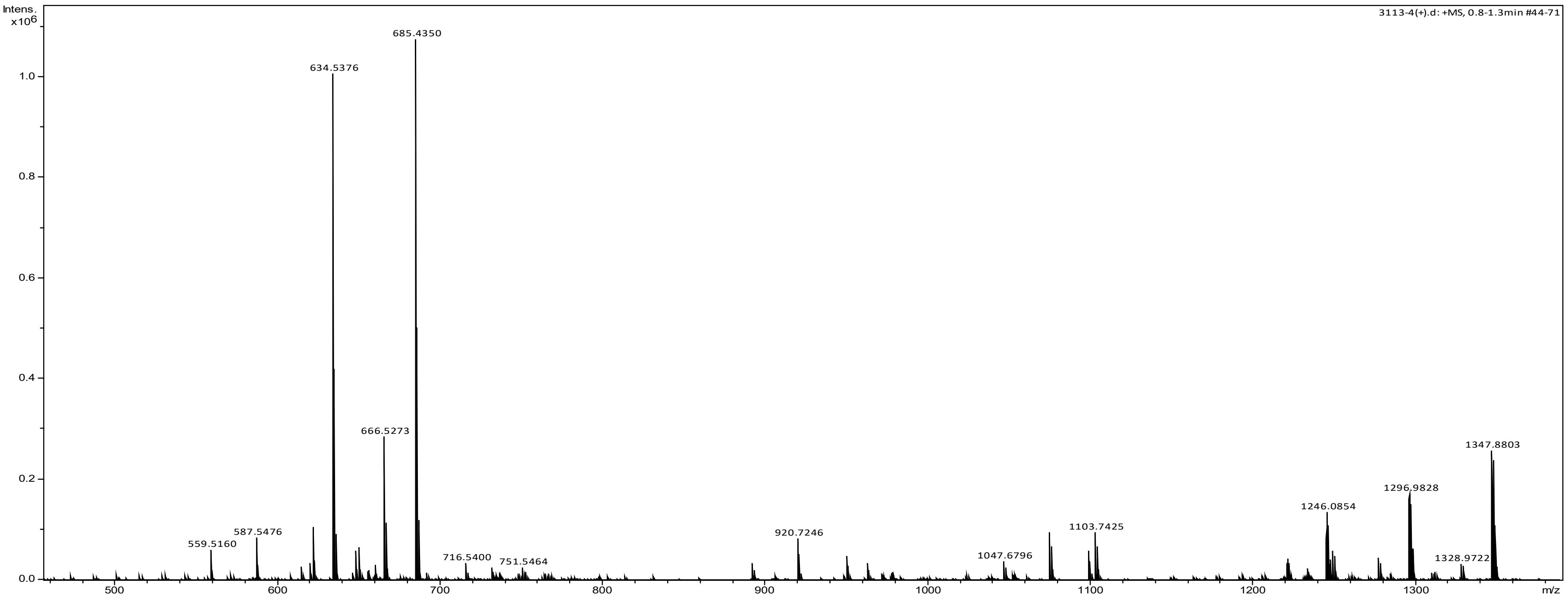

Figure S3. ^{1}H-NMR spectrum of ceramide **1** in C_5D_5N.

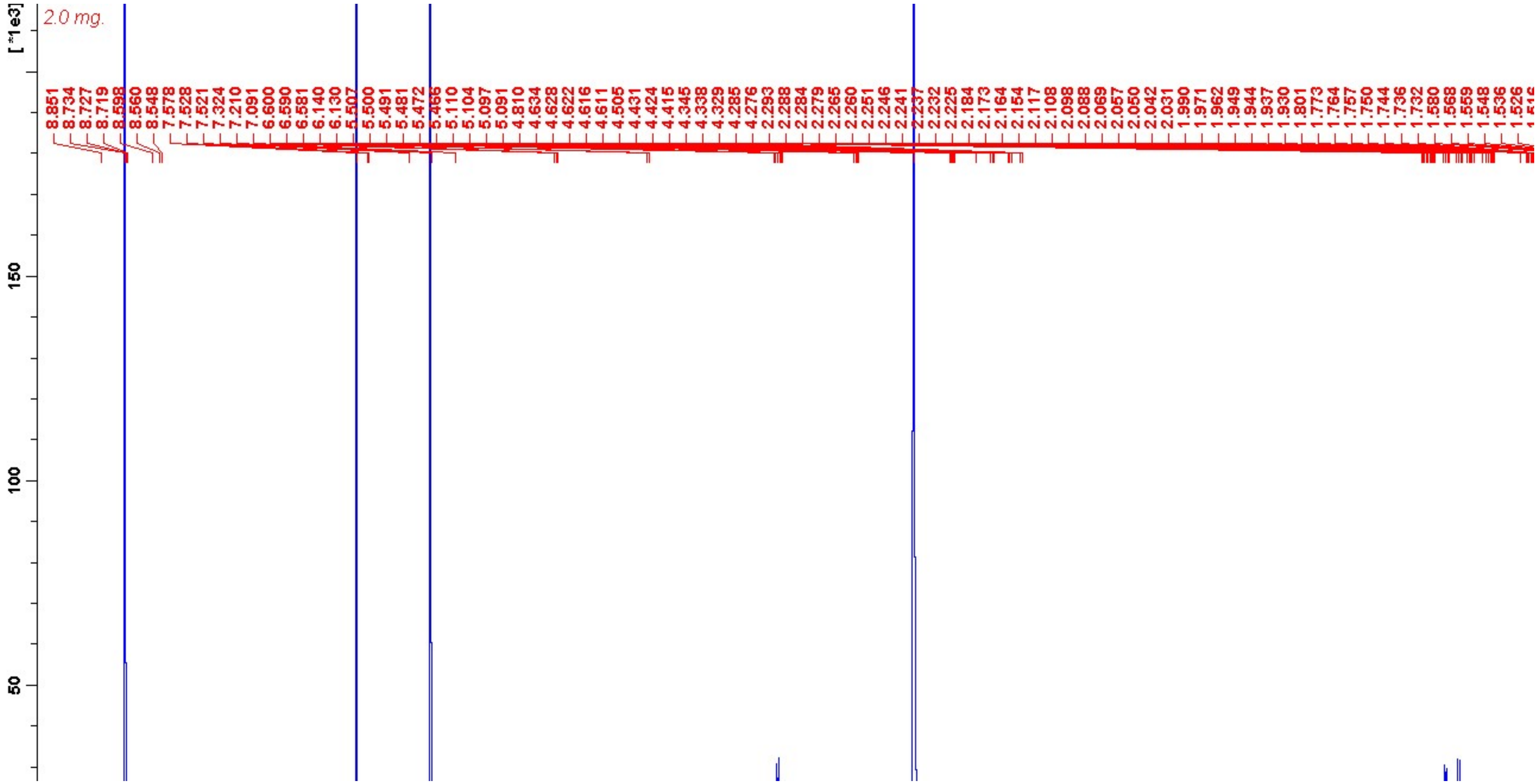

Figure S4. ^{13}C-NMR spectrum of ceramide **1** in C_5D_5N.

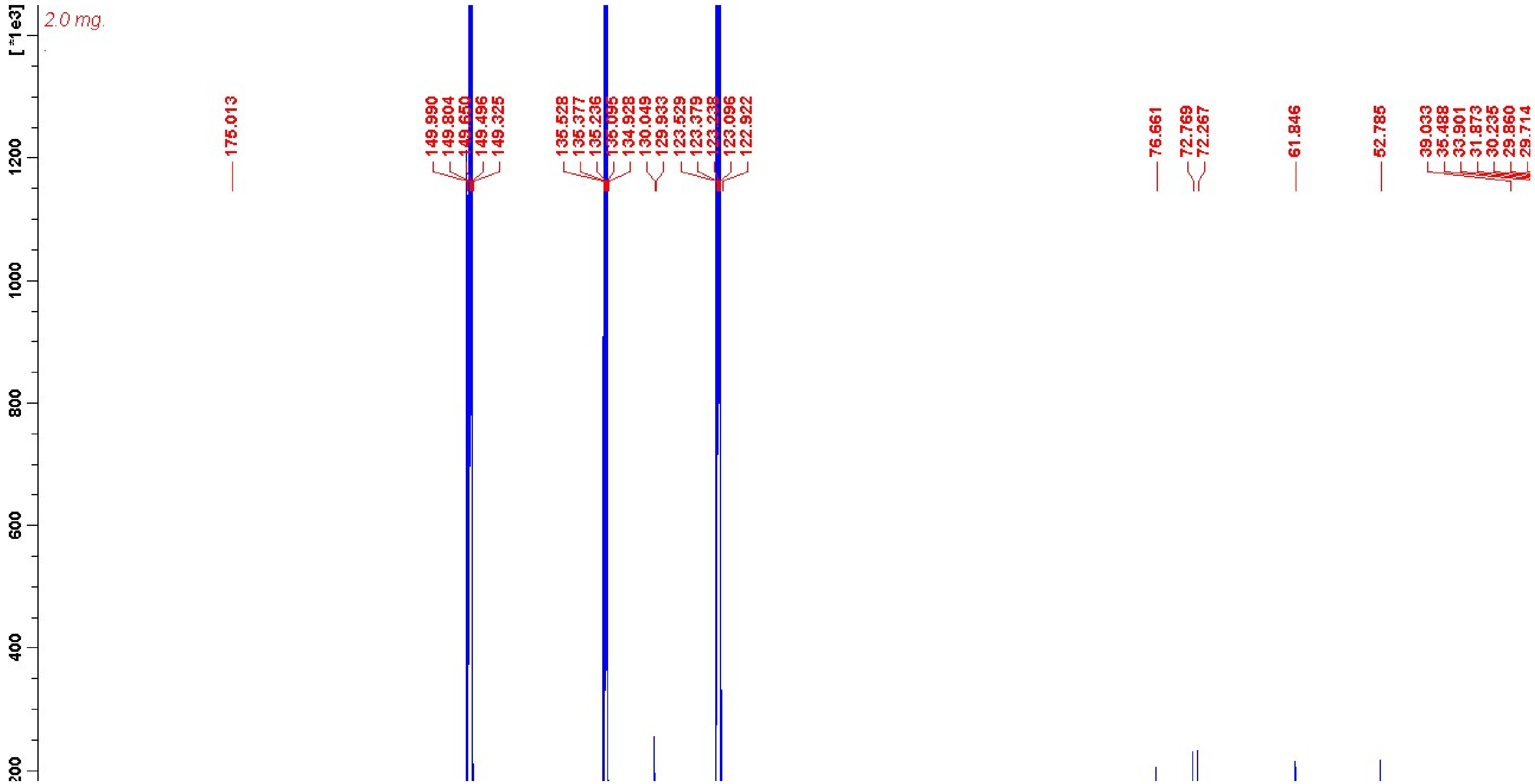

Figure S5. ^{1}H-^{1}H-COSY spectrum of ceramide **1** in C_5D_5N.

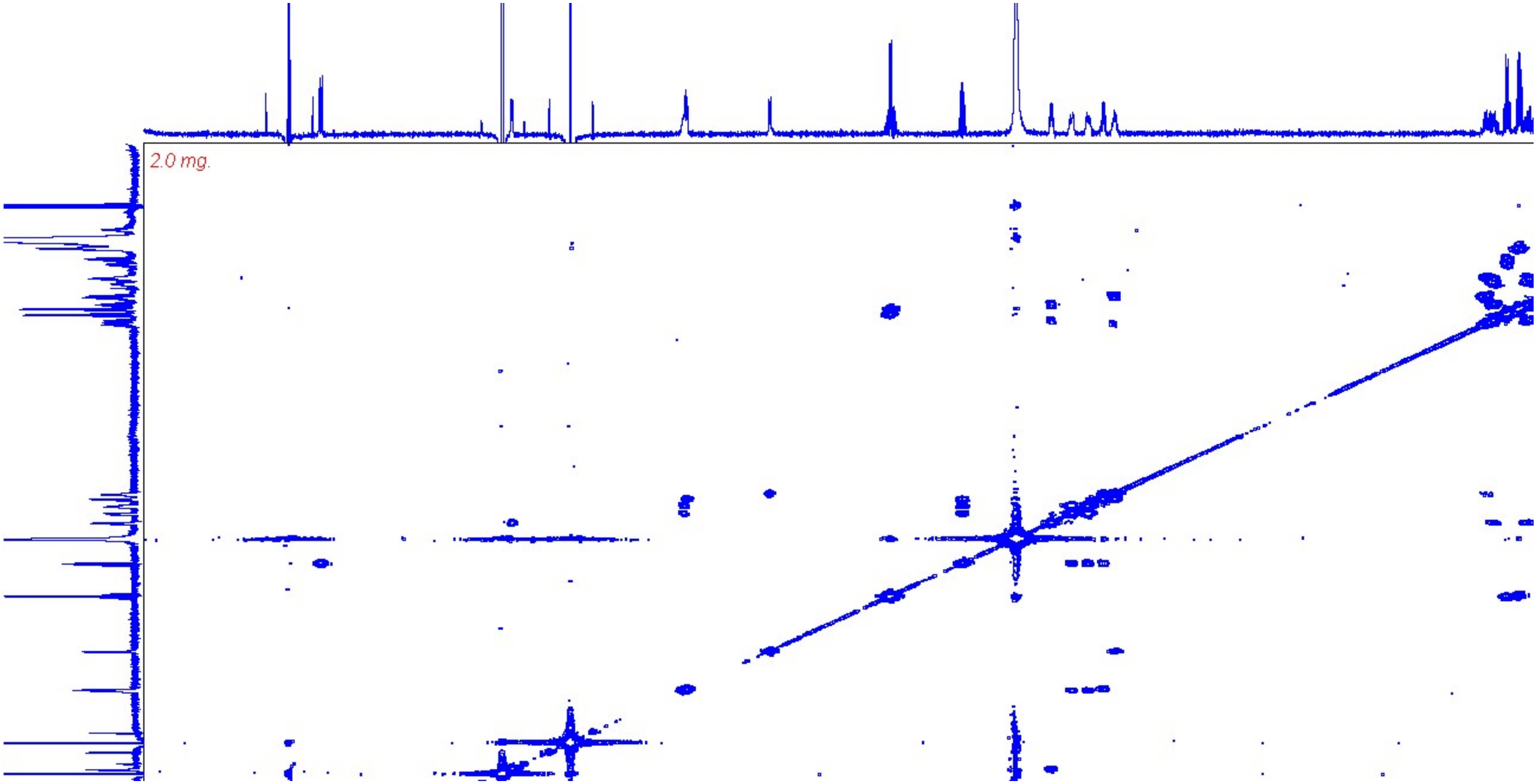

Figure S6. HSQC spectrum of ceramide **1** in C_5D_5N.

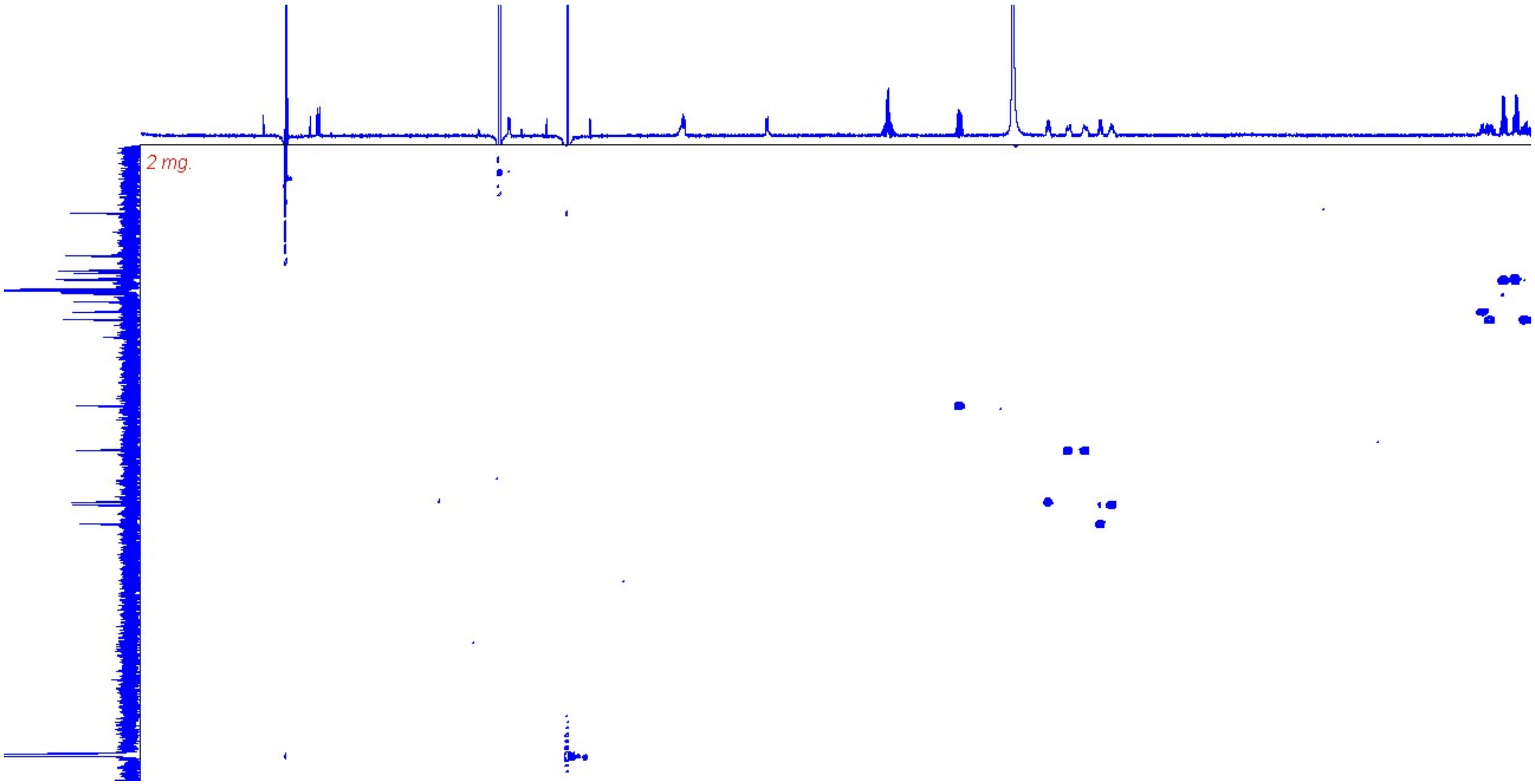

Figure S7. HMBC spectrum of ceramide **1** in C_5D_5N.

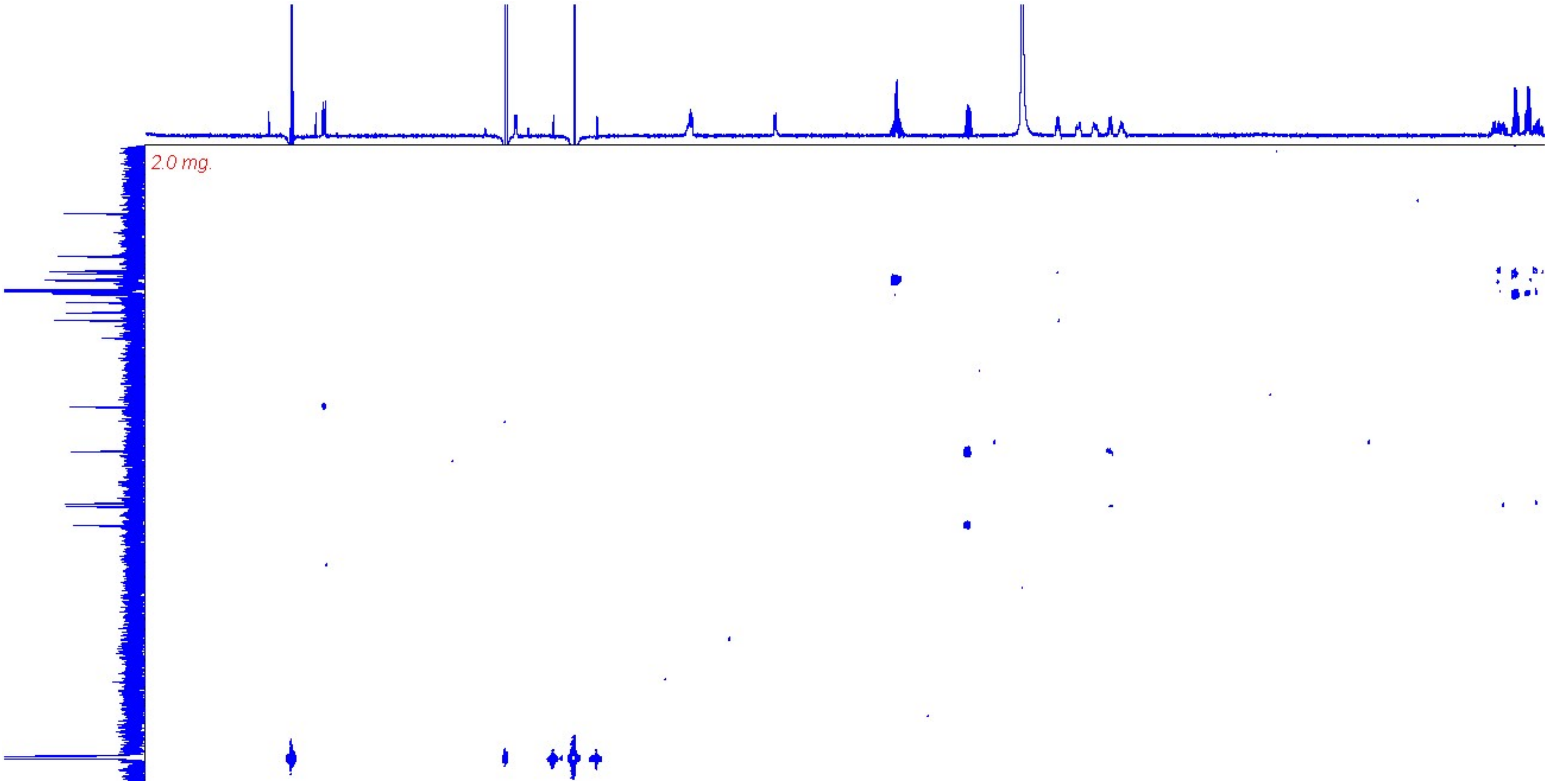

Figure S8. 2D TOCSY spectrum of ceramide **1** in C_5D_5N.

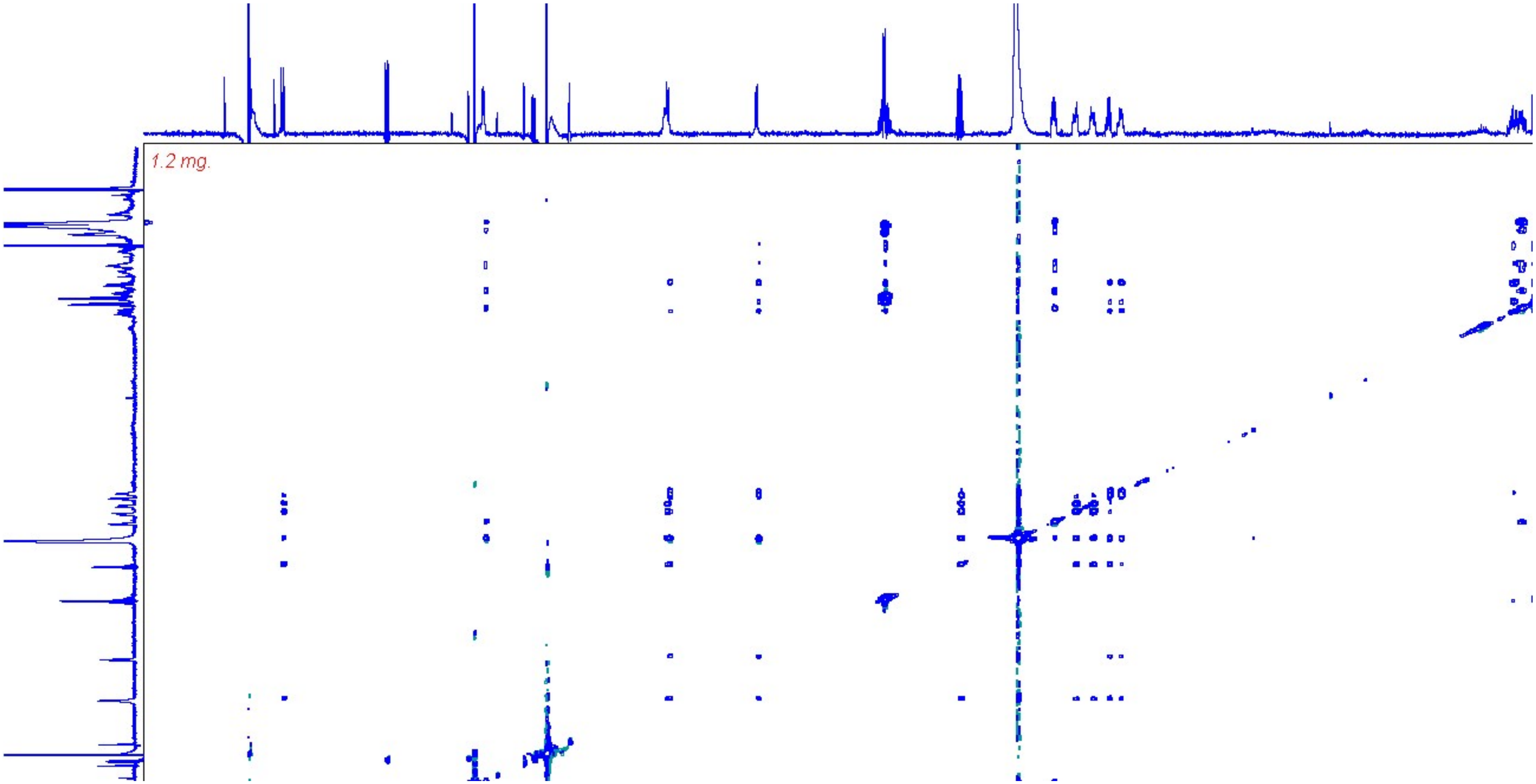

Figure S9. (–)-HRESIMS spectrum of ceramide **2**.

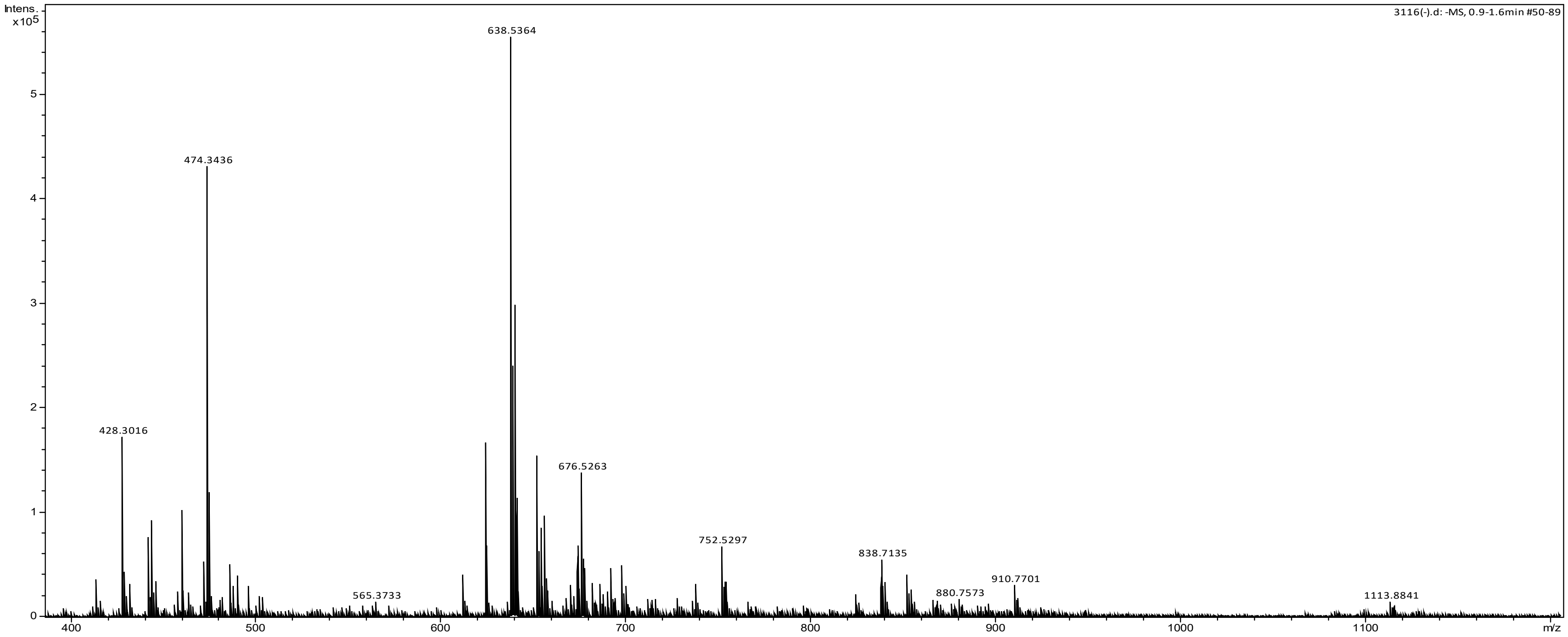

Figure S10. (+)-HRESIMS spectrum of ceramide **2.**

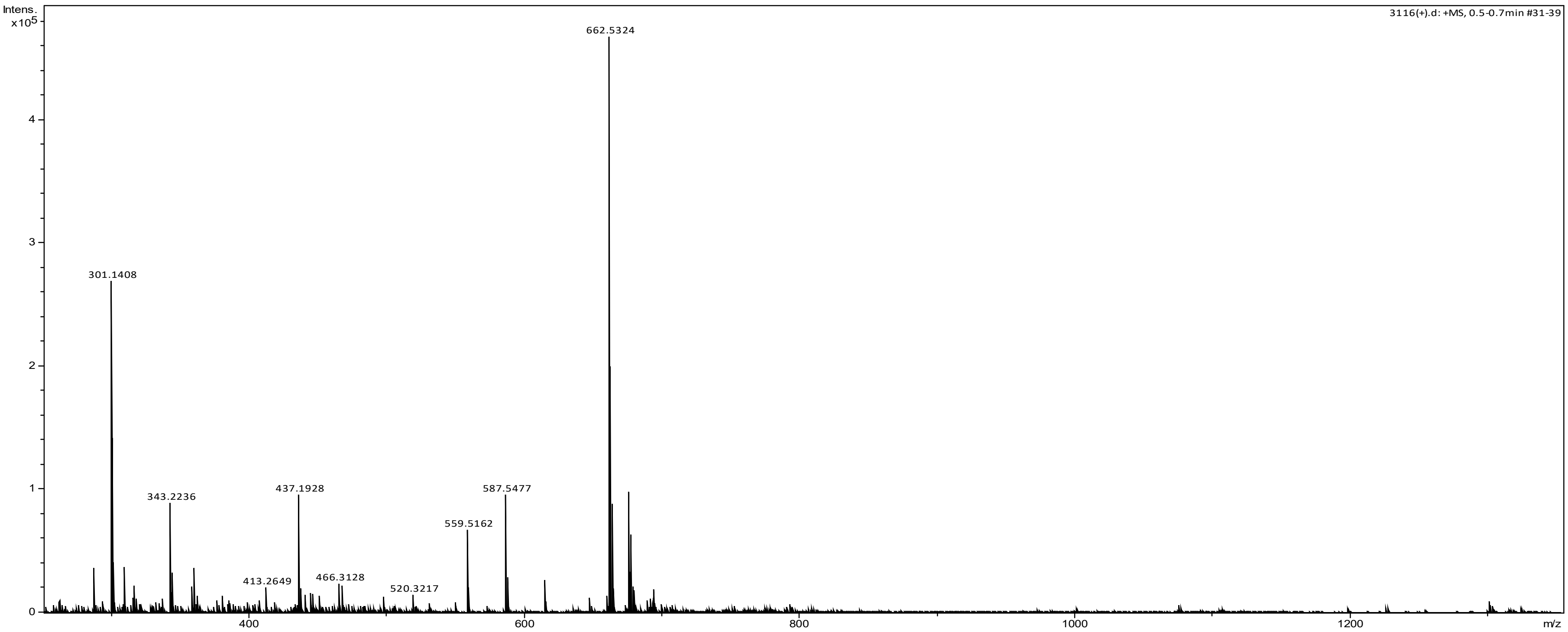

Figure S11. ^{1}H-NMR spectrum of ceramide **2** in C_5D_5N.

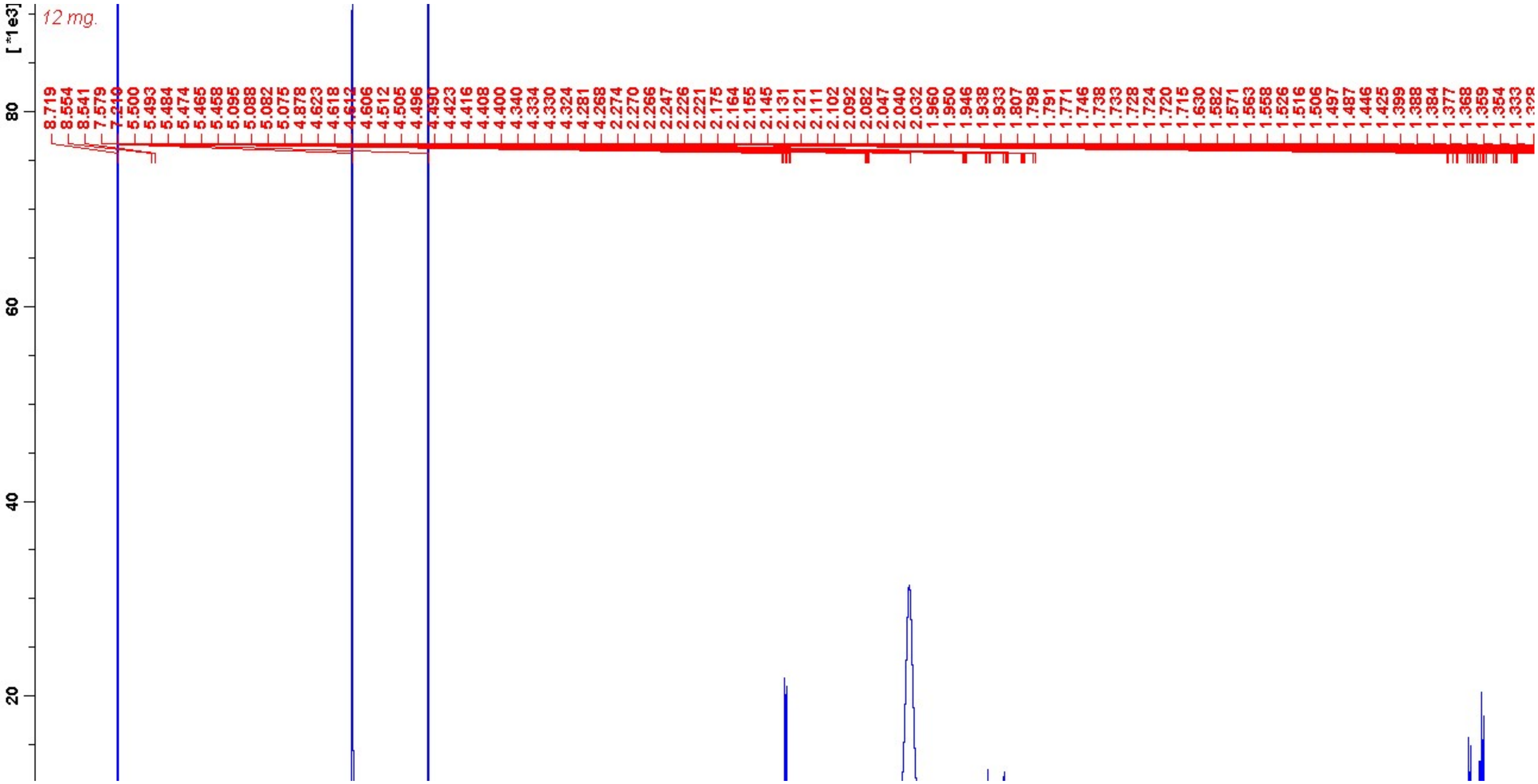

Figure S12. ^{13}C-NMR spectrum of ceramide **2** in C_5D_5N.

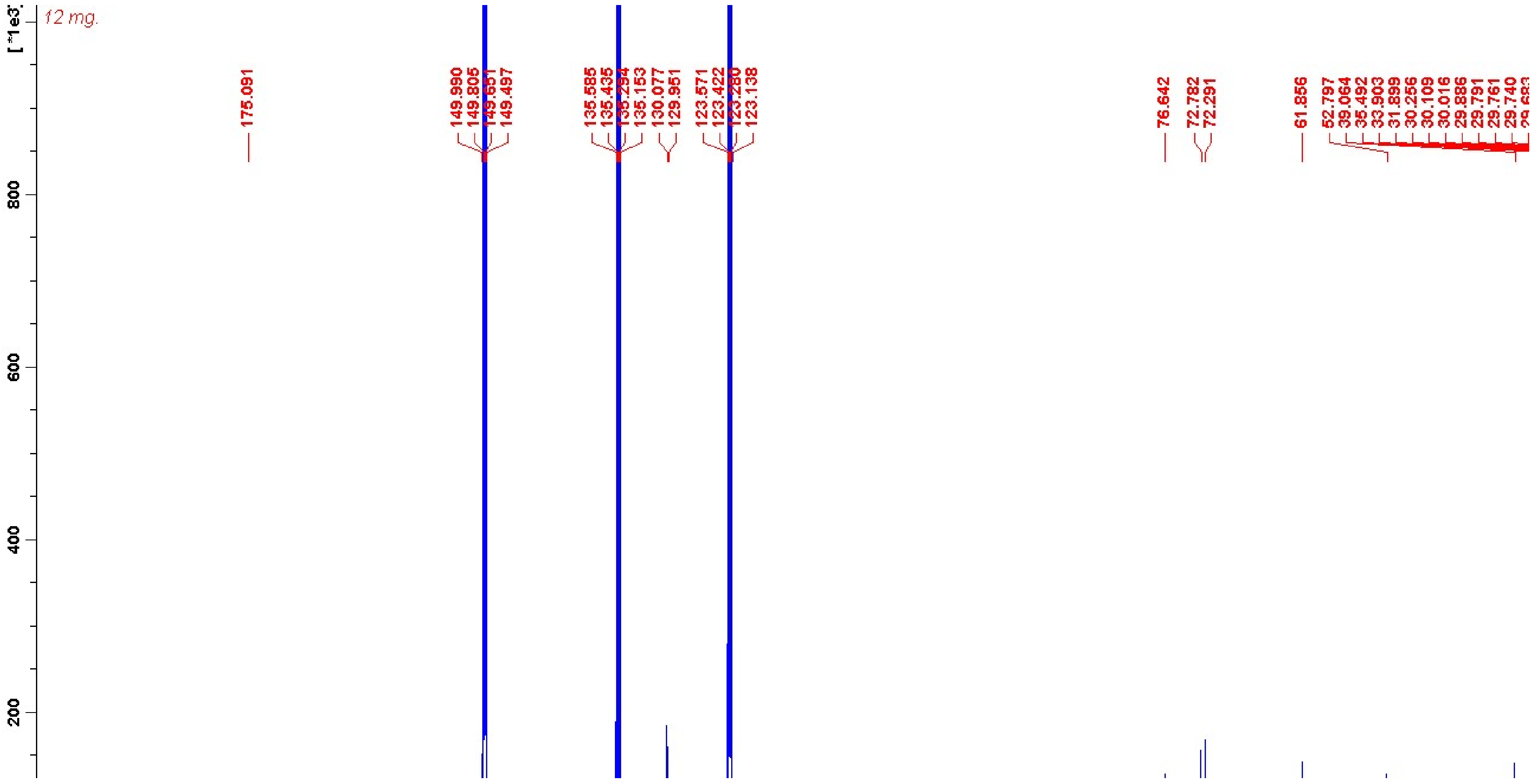

Figure S13. ^1H-^1H-COSY spectrum of ceramide **2** in C_5D_5N.

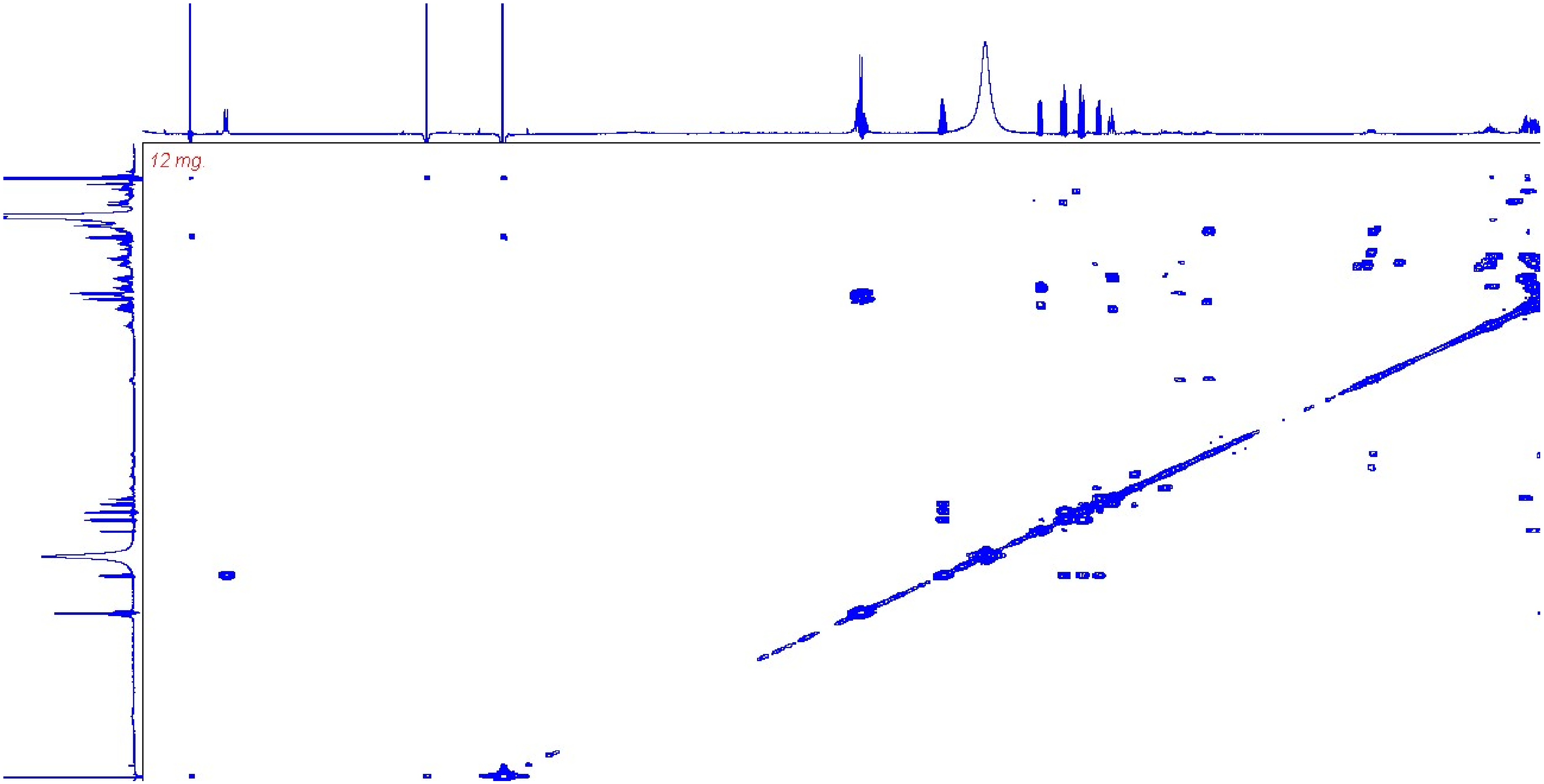

Figure S14. HSQC spectrum of ceramide **2** in C_5D_5N.

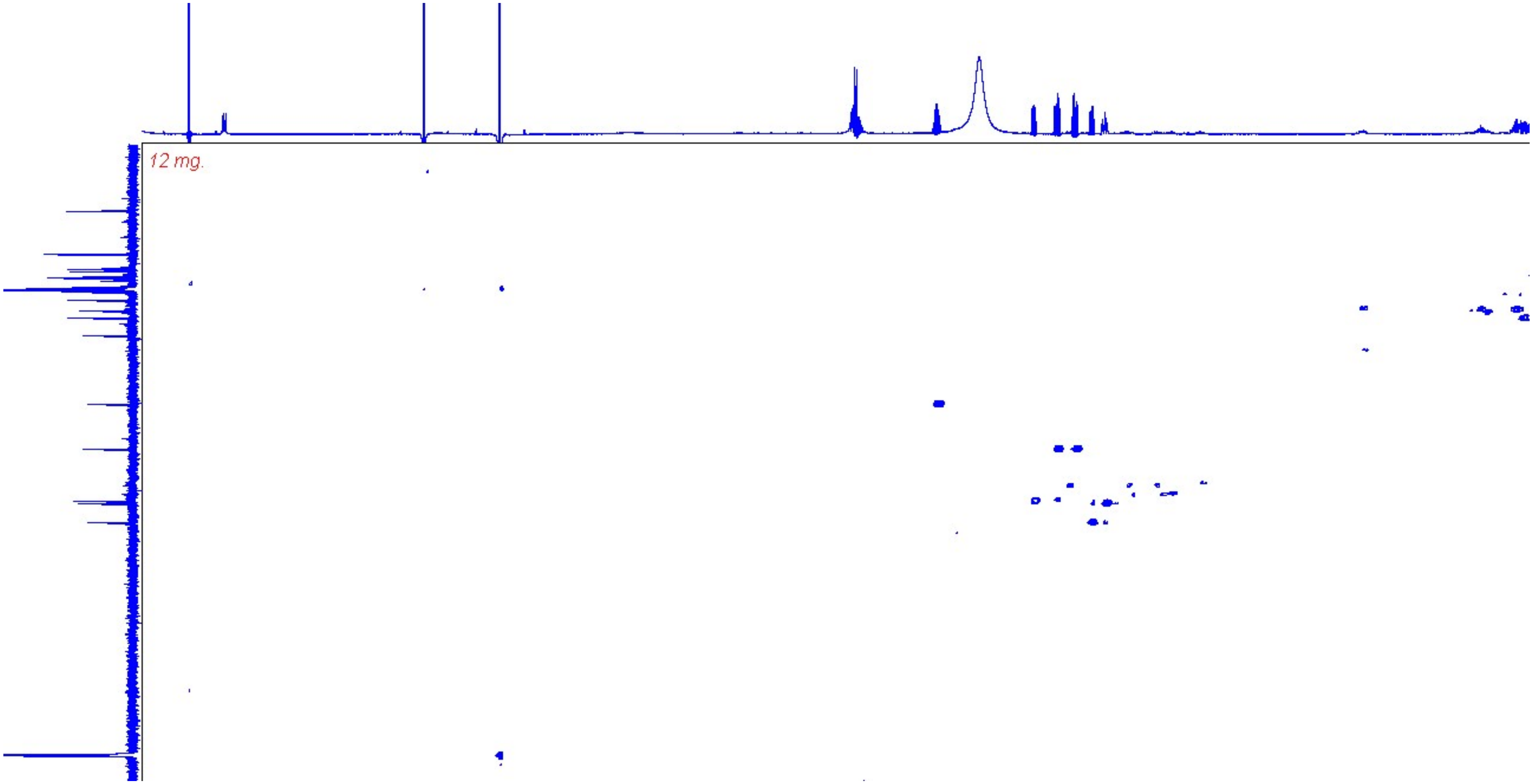

Figure S15. HMBC spectrum of ceramide **2** in C_5D_5N.

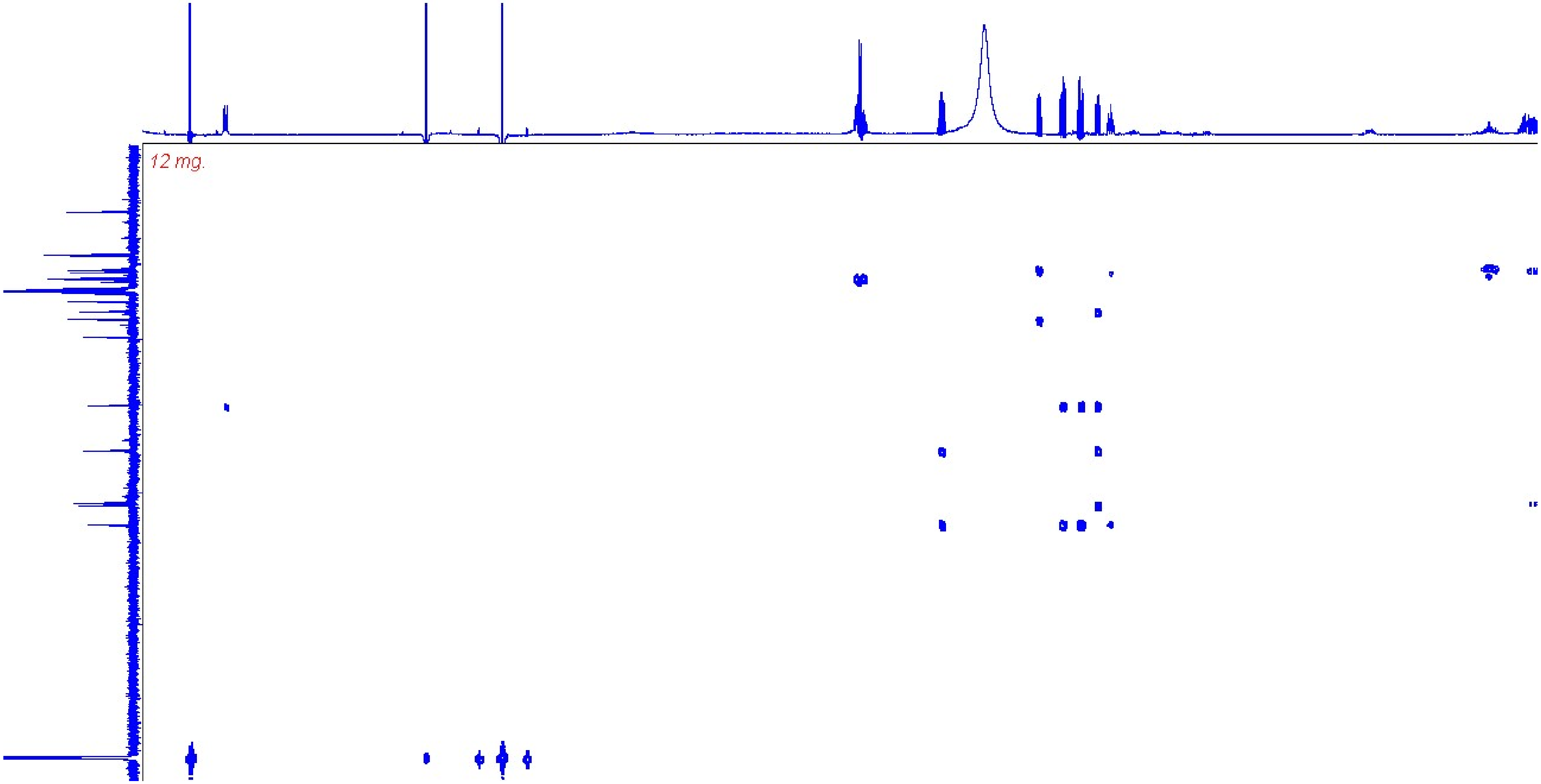

Figure S16. (–)-HRESIMS spectrum of ceramide **3**.

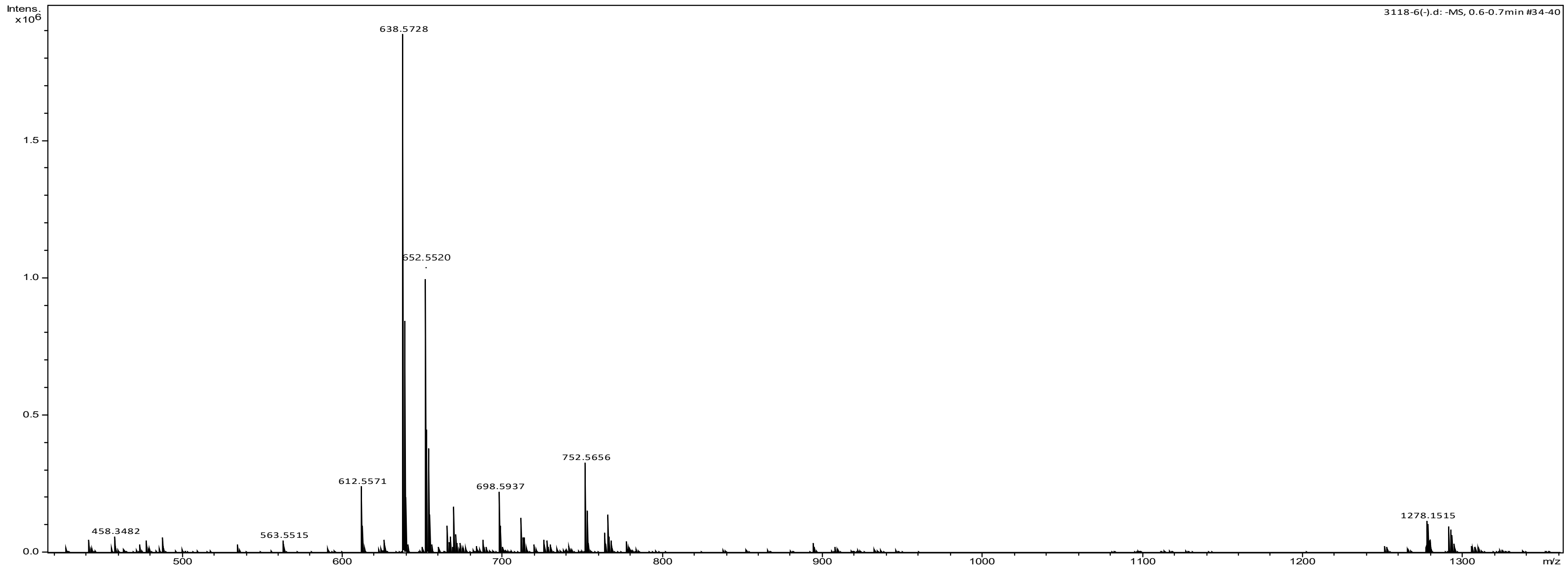

Figure S17. (+)-HRESIMS spectrum of ceramide **3**.

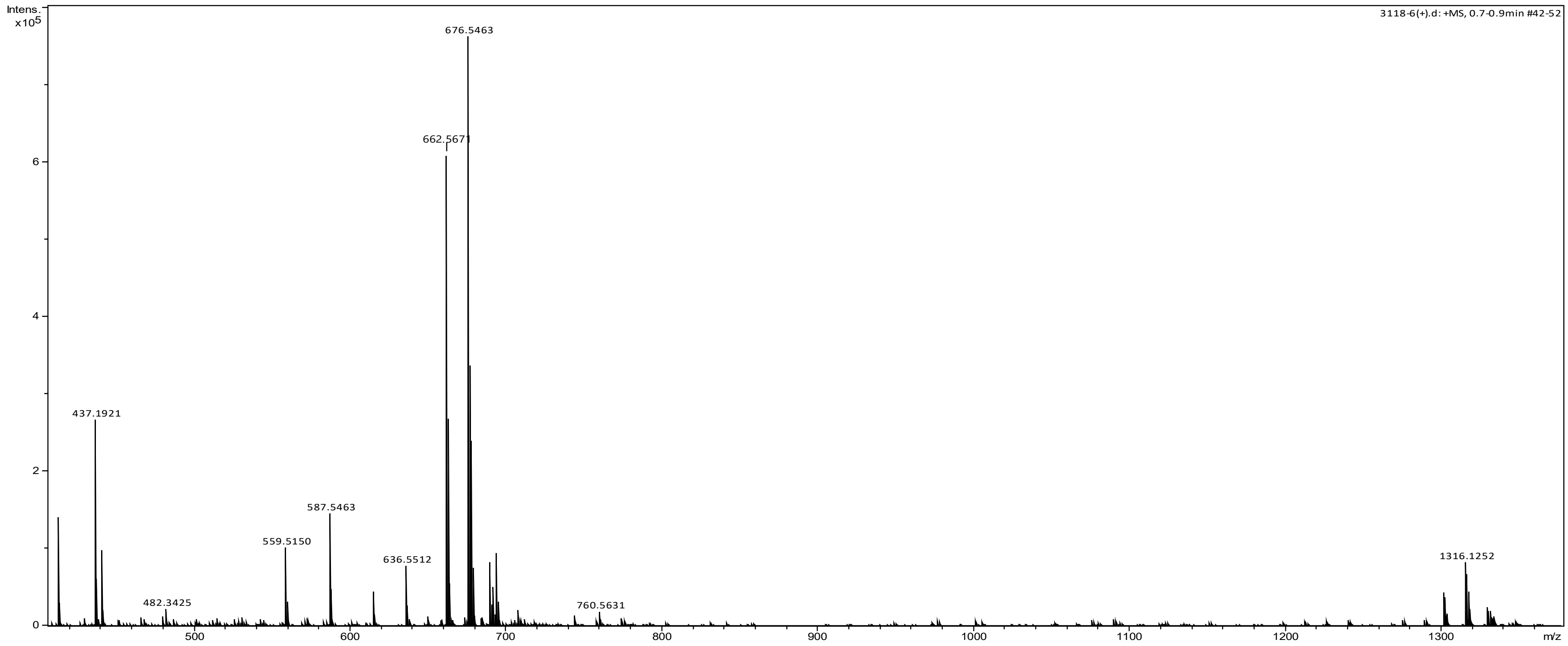

Figure S18. ^{1}H-NMR spectrum of ceramide **3** in C_5D_5N.

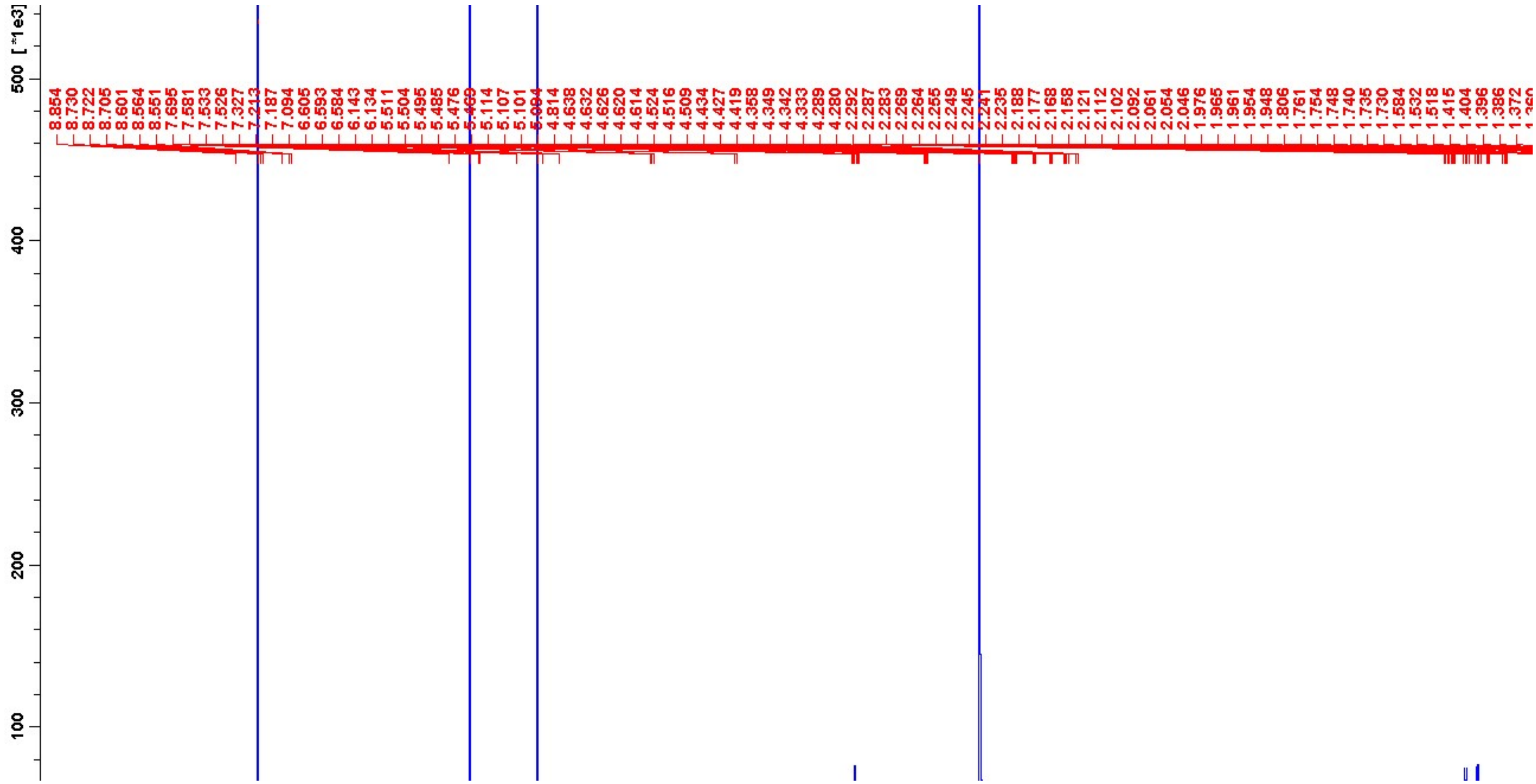

Figure S19. ^{13}C-NMR spectrum of ceramide **3** in C_5D_5N.

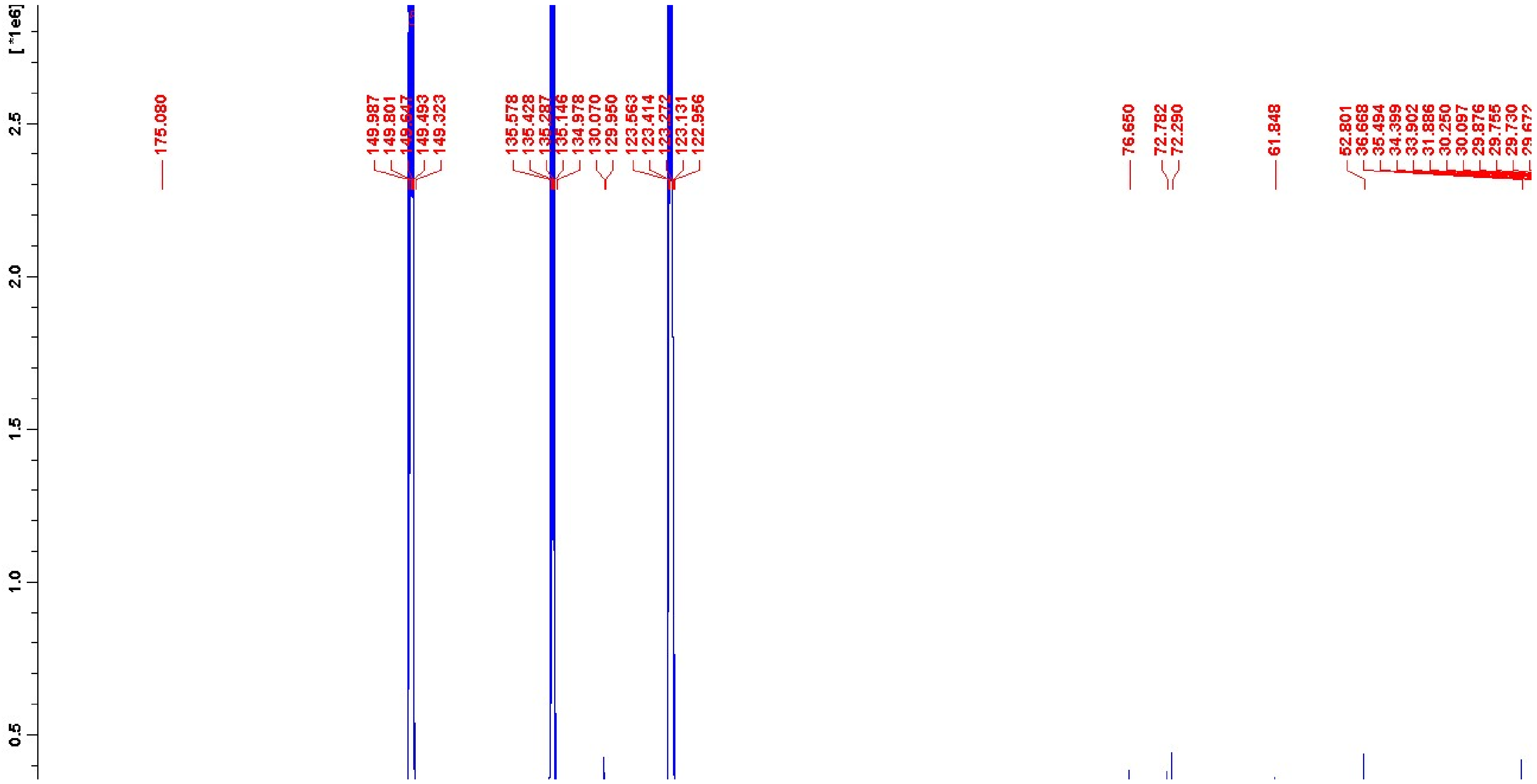

Figure S20. ^1H-^1H-COSY spectrum of ceramide **3** in C_5D_5N.

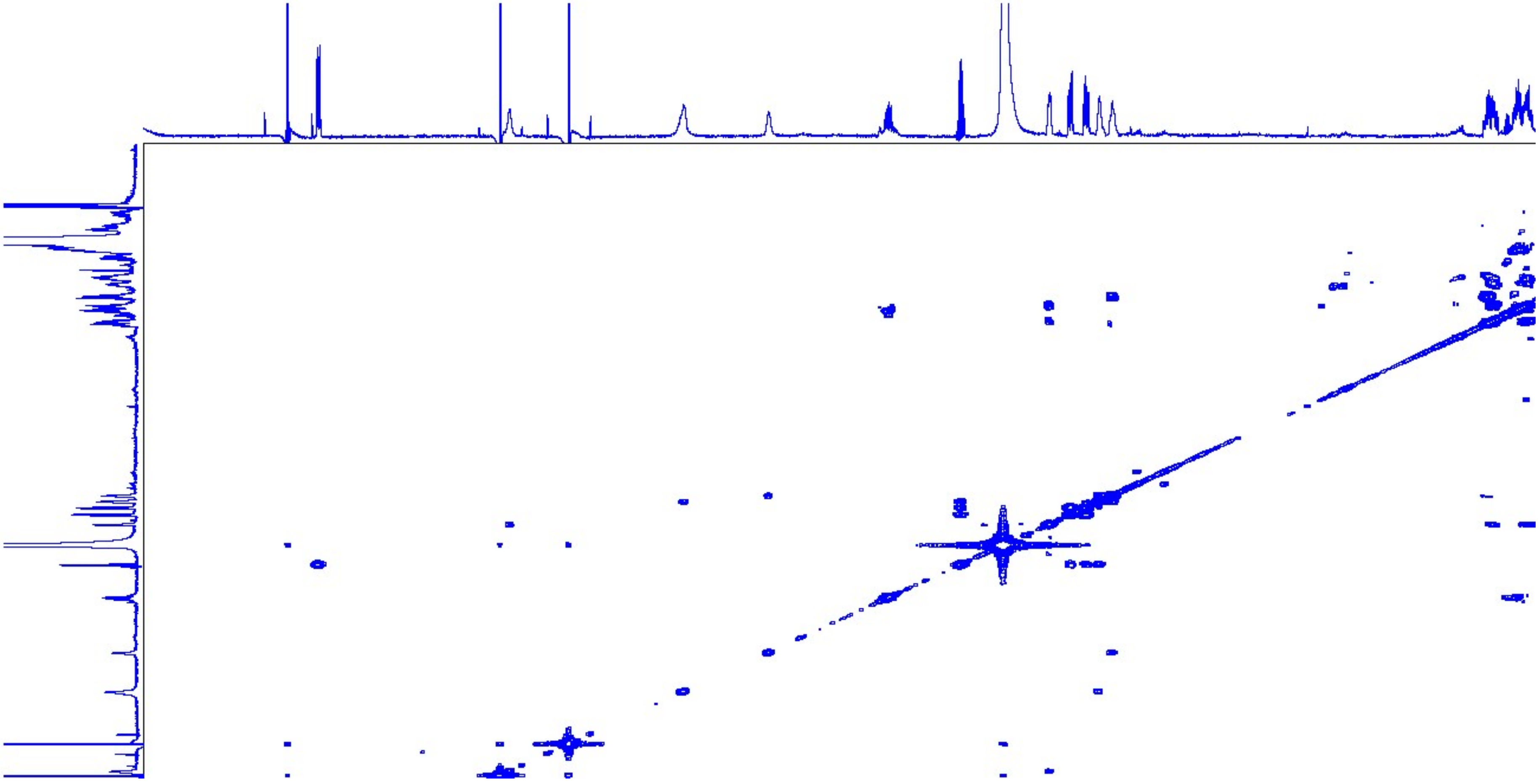

Figure S21. HSQC spectrum of ceramide **3** in C_5D_5N.

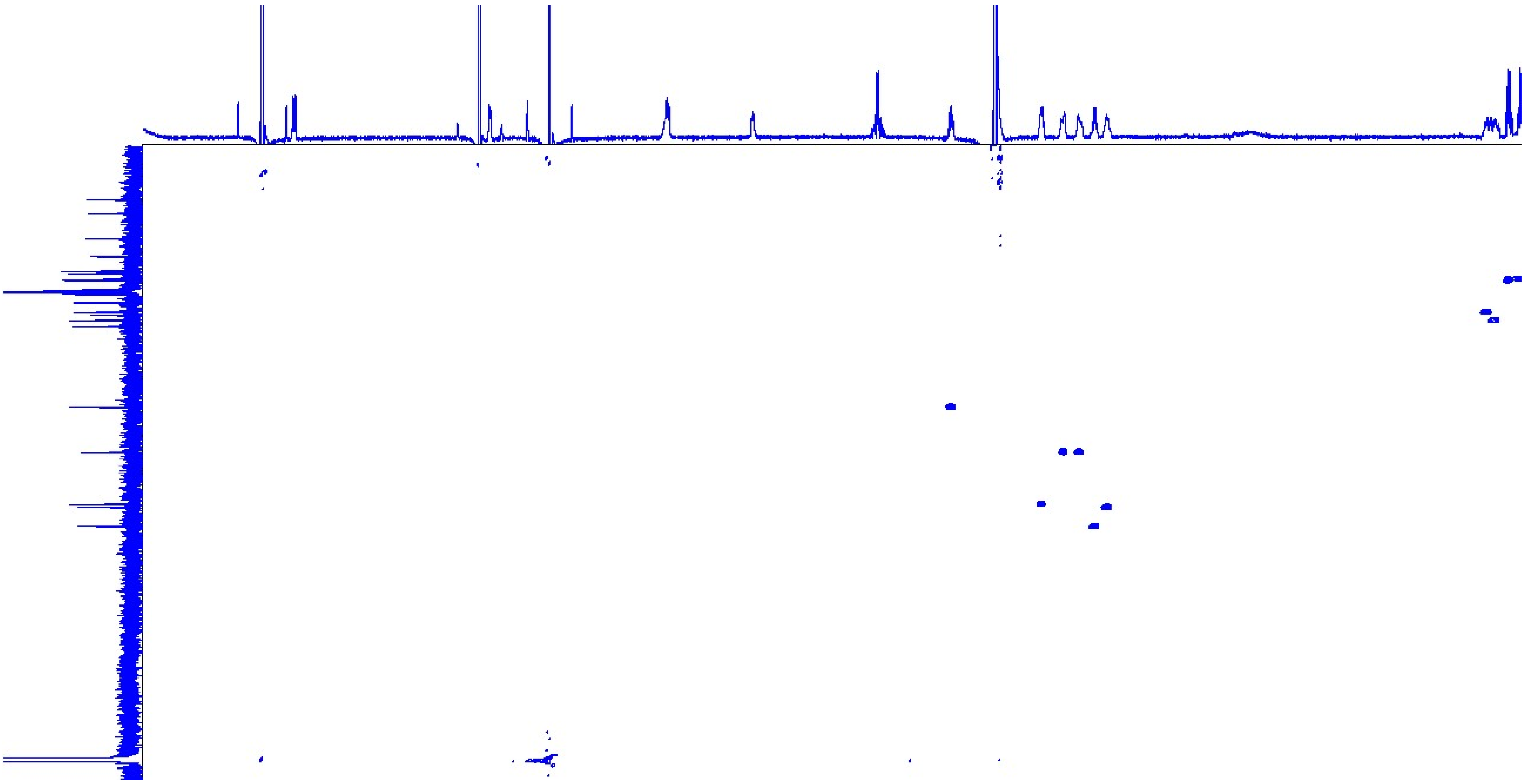

Figure S22. HMBC spectrum of ceramide **3** in C_5D_5N.

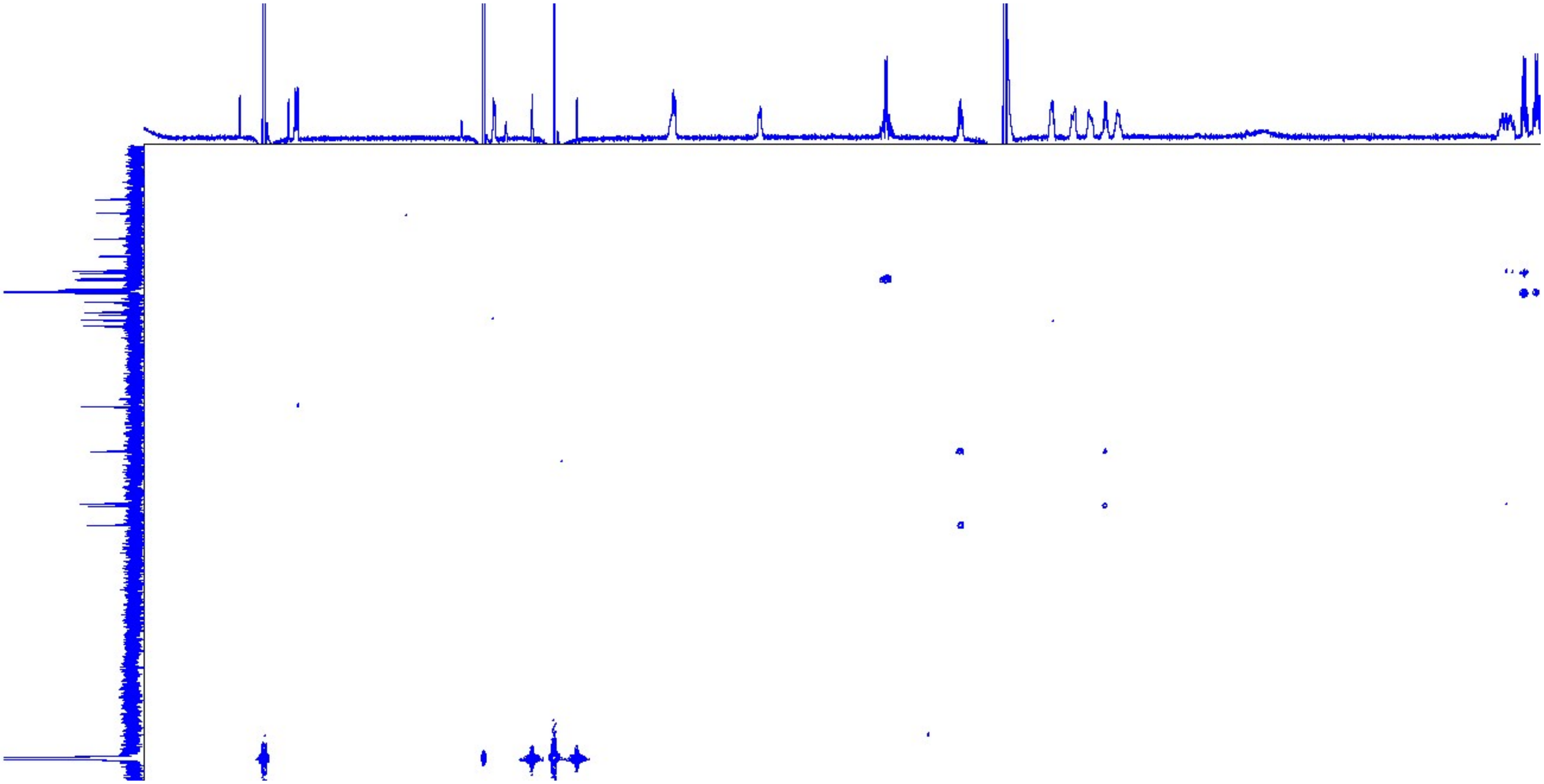

Figure S23. (–)-HRESIMS spectrum of cerebroside **4**.

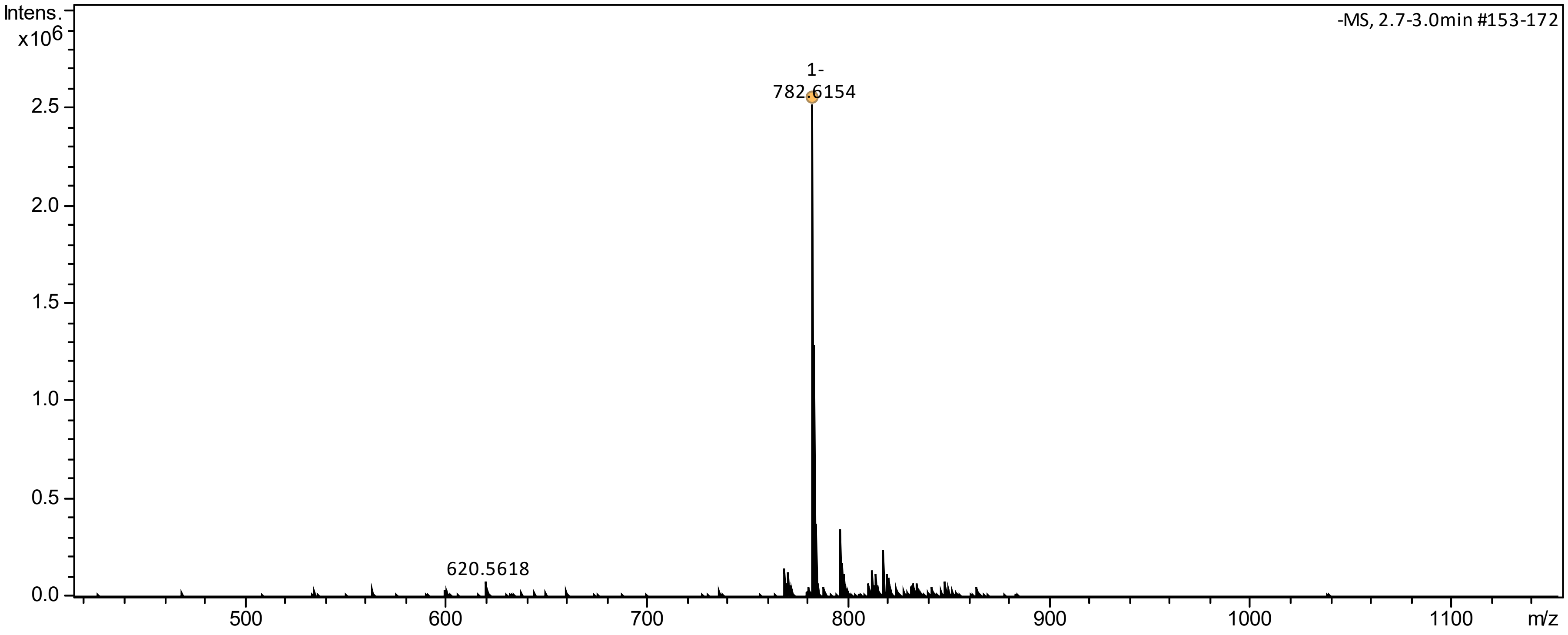

Figure S24. (+)-HRESIMS spectrum of cerebroside **4**.

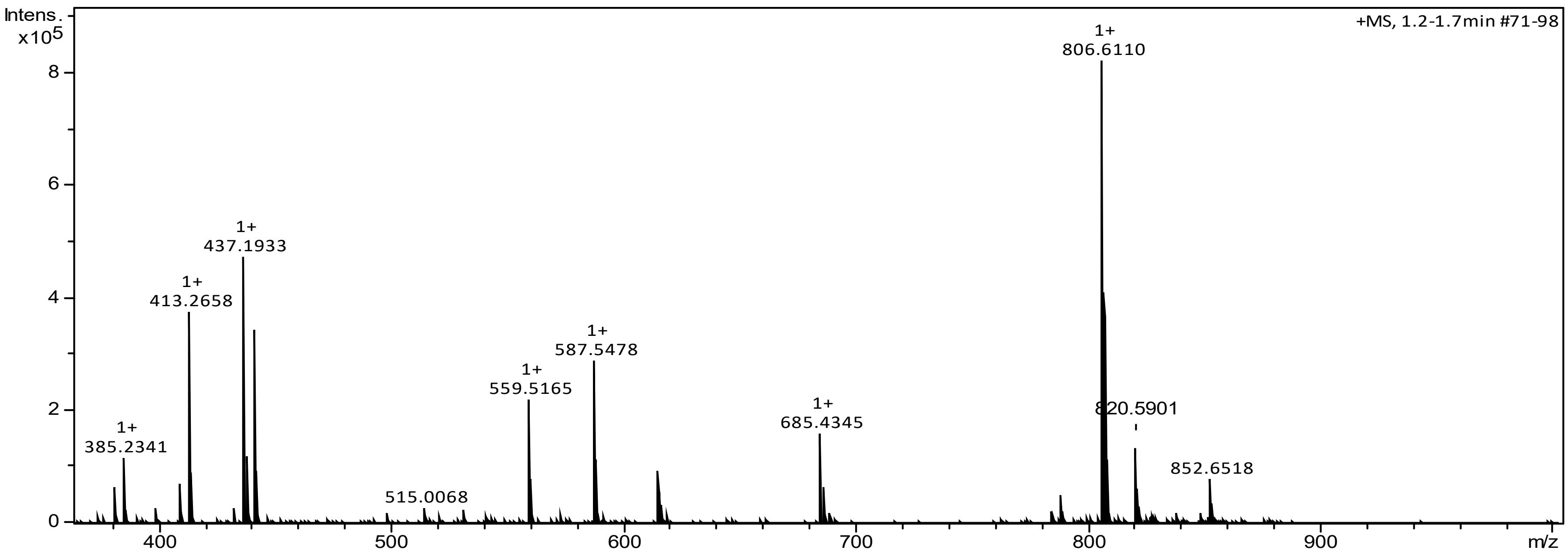

Figure S25. ^{1}H-NMR spectrum of cerebroside **4** in C_5D_5N.

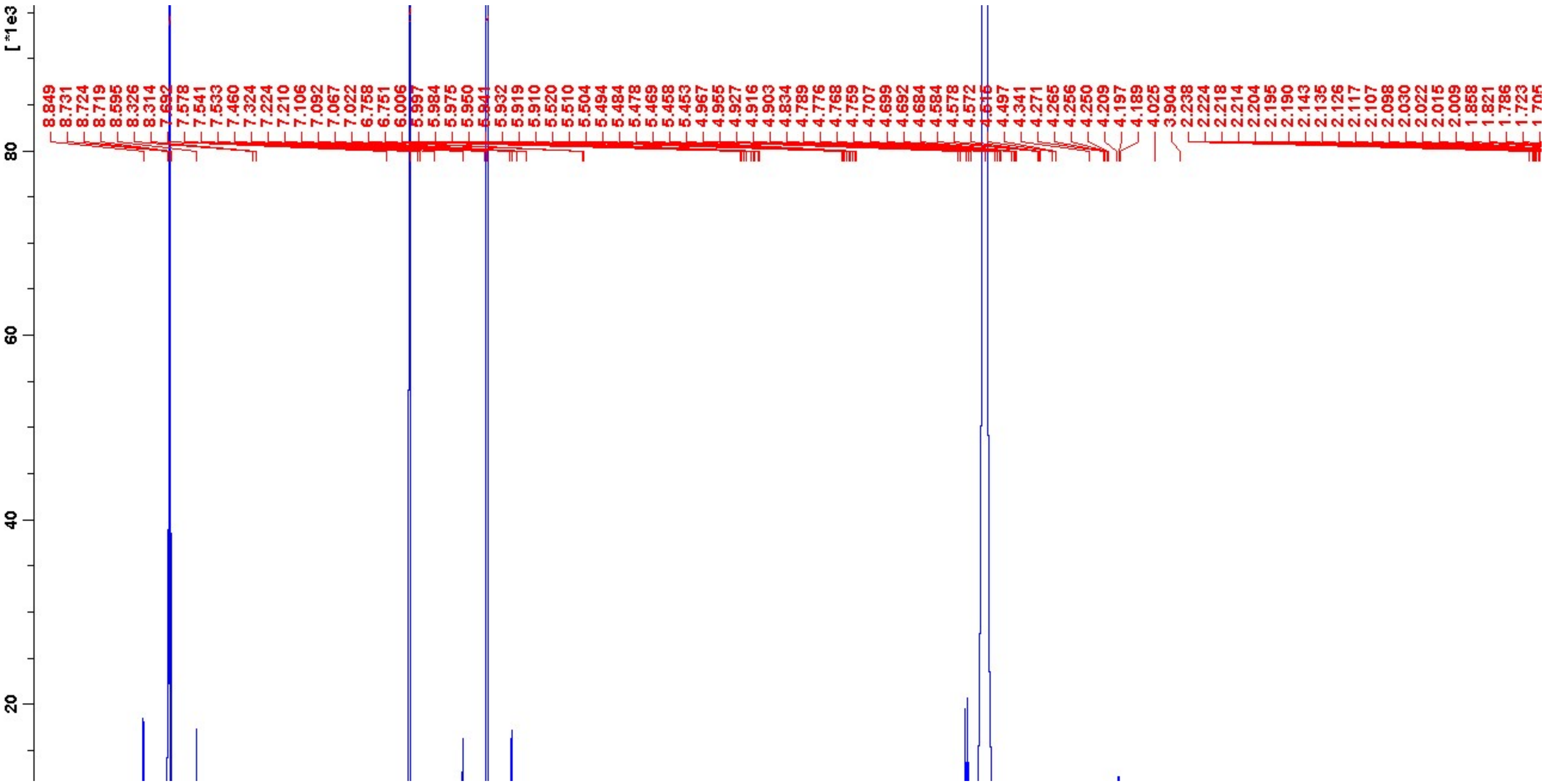

Figure S26. ^{13}C-NMR spectrum of cerebroside **4** in C_5D_5N.

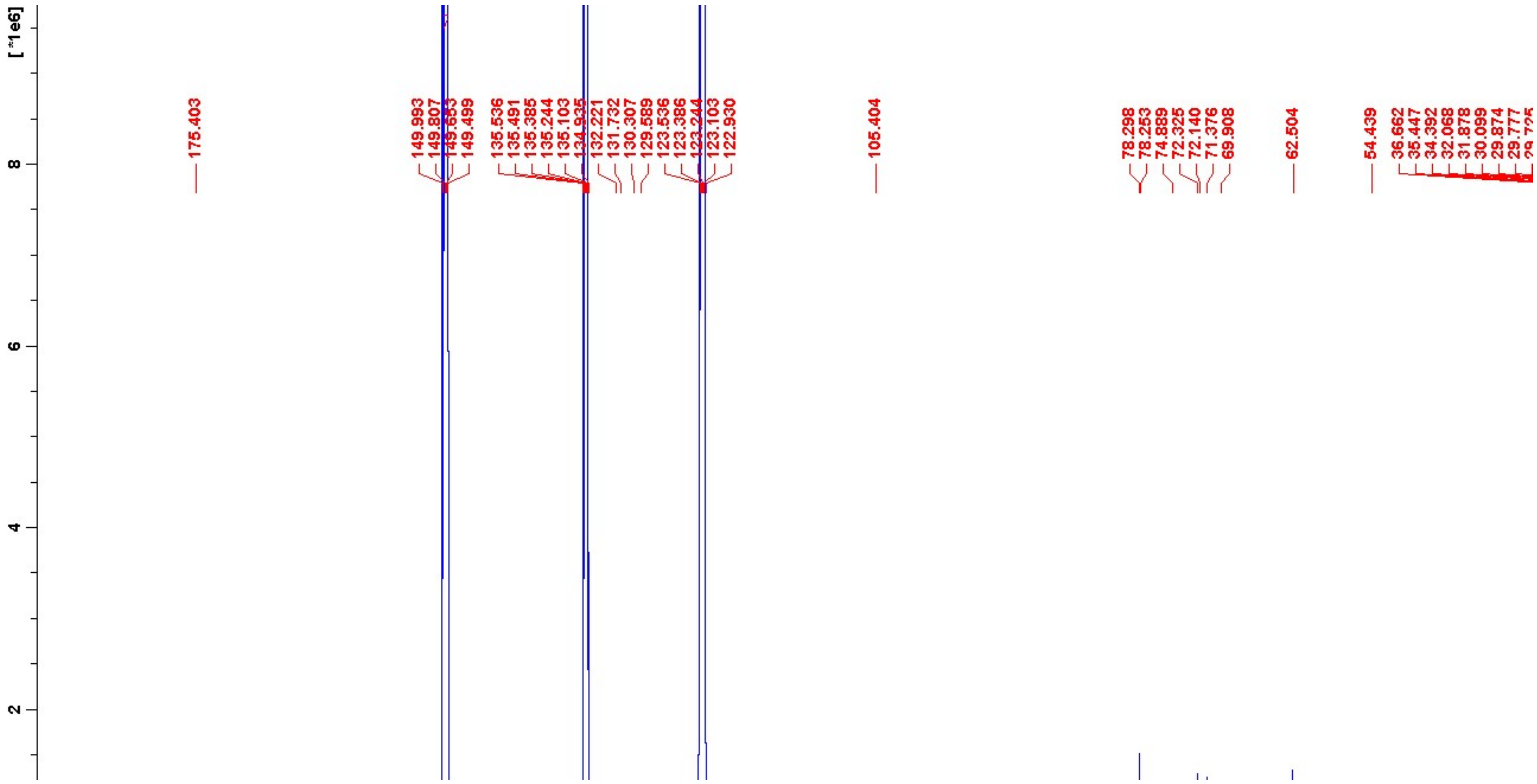

Figure S27. ^{1}H-^{1}H-COSY spectrum of cerebroside **4** in C_5D_5N.

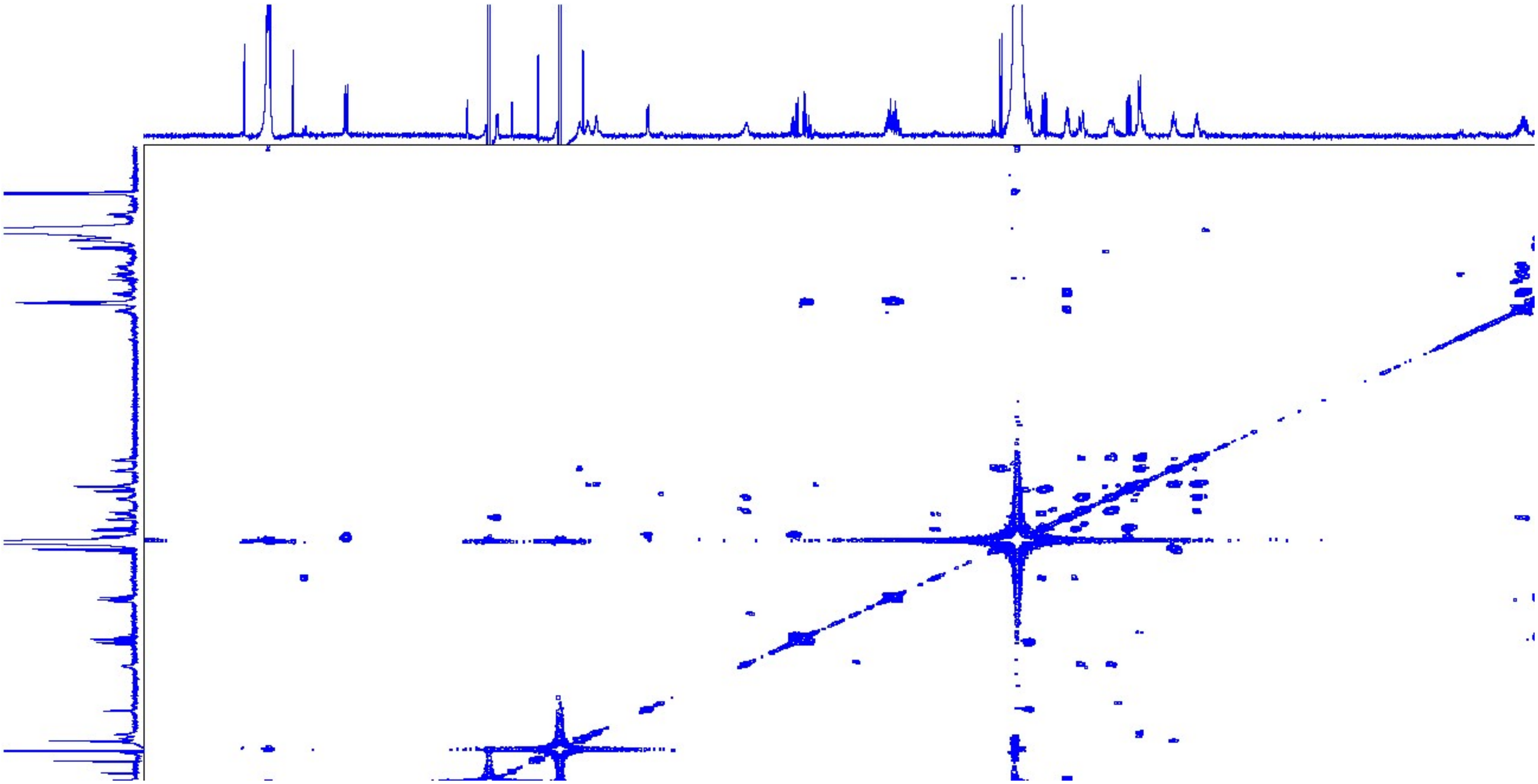

Figure S28. HSQC spectrum of cerebroside **4** in C_5D_5N.

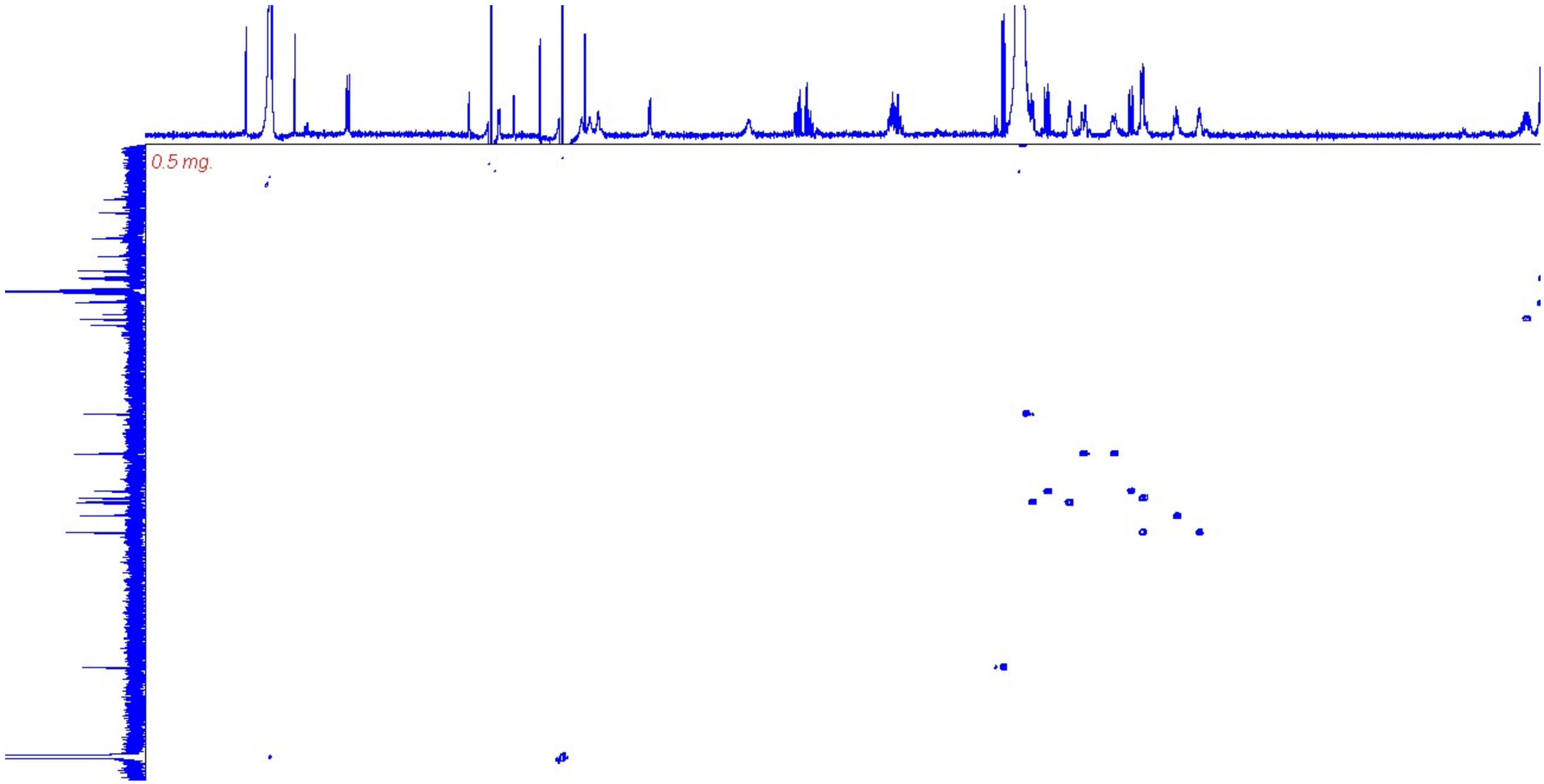

Figure S29. HMBC spectrum of cerebroside **4** in C_5D_5N.

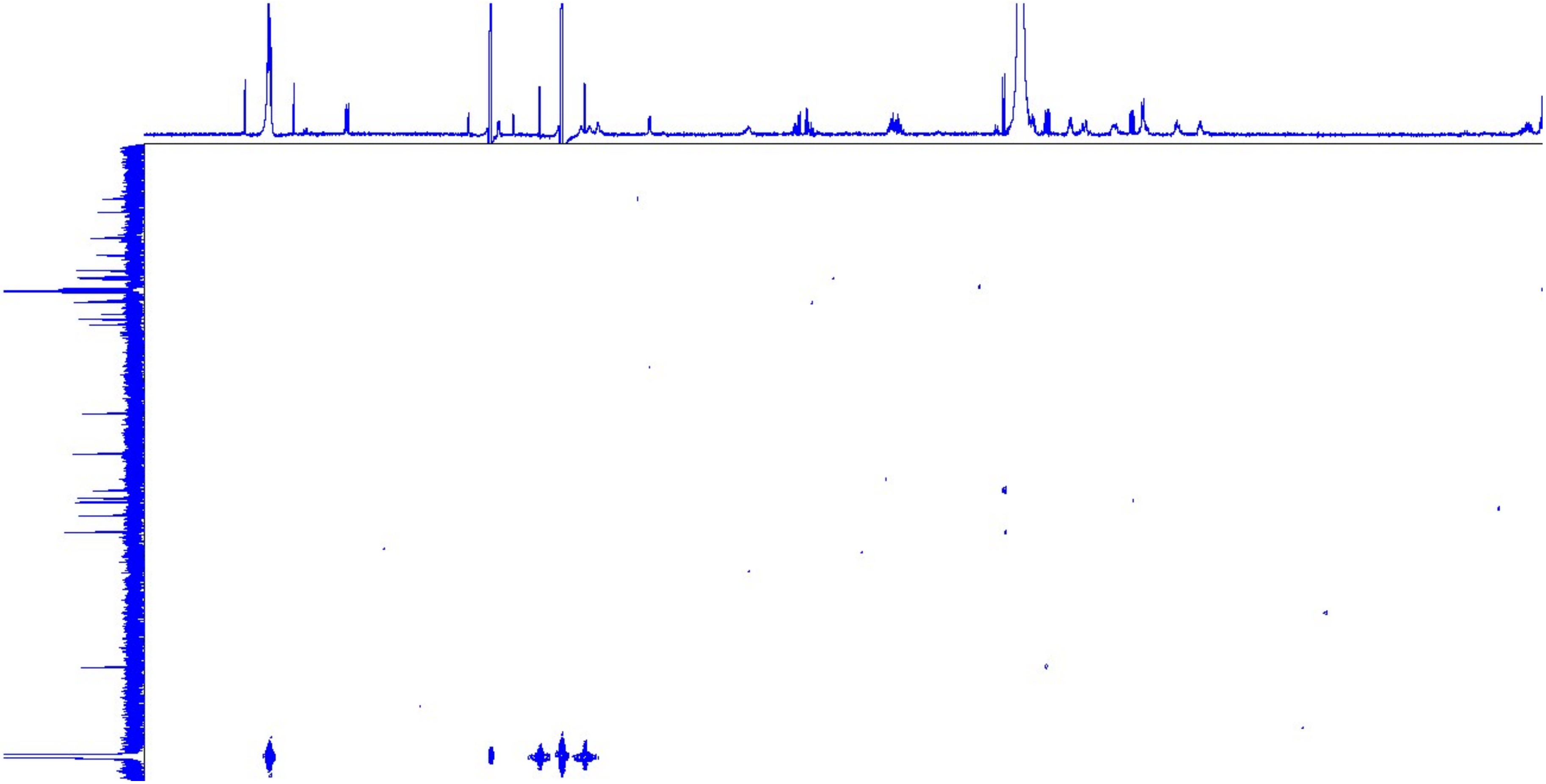

Figure S30. (–)-HRESIMS spectrum of cerebroside **8**.

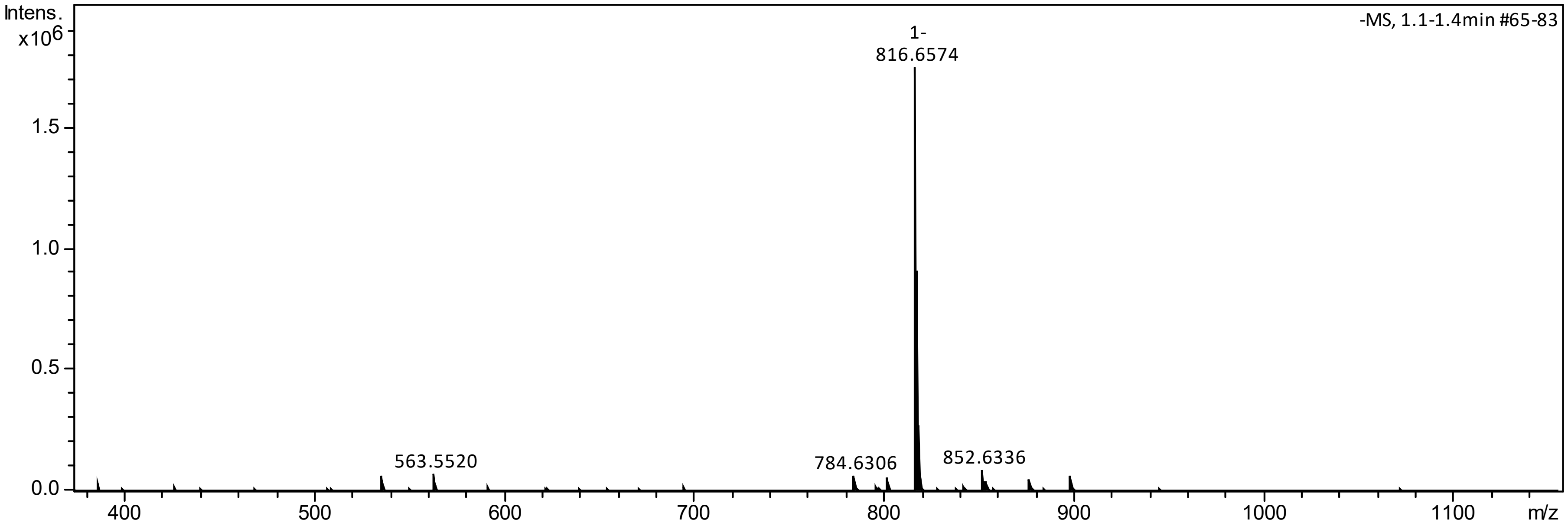

Figure S31. (+)-HRESIMS spectrum of cerebroside **8**.

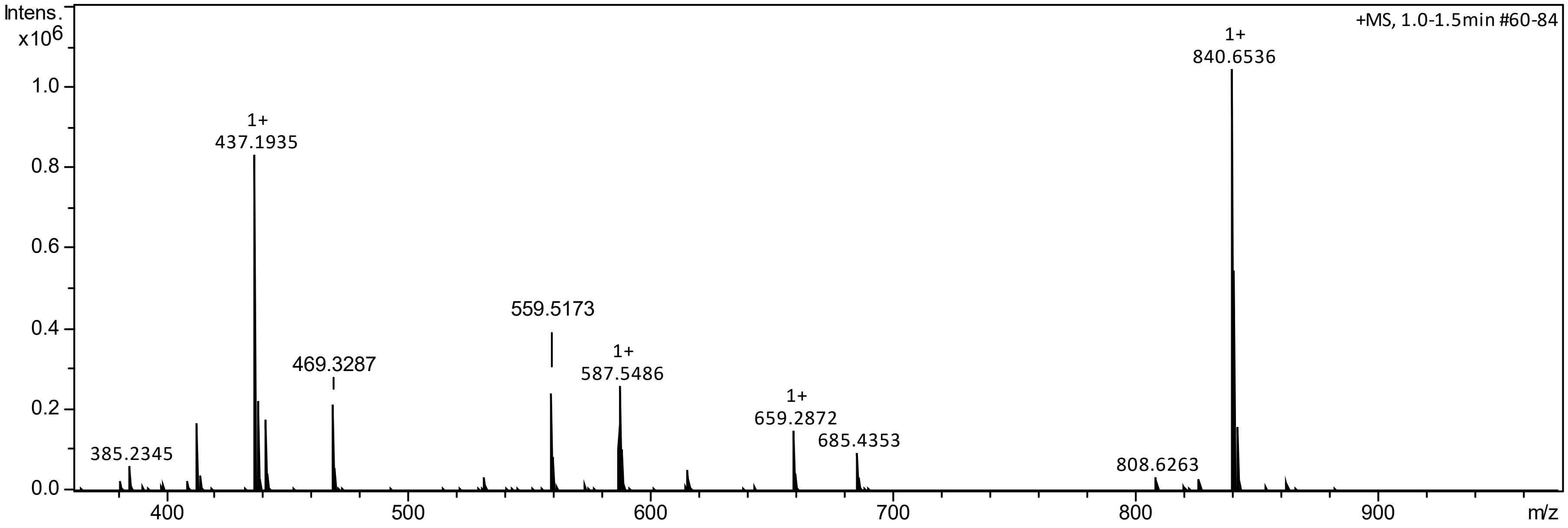

Figure S32. ^{1}H-NMR spectrum of cerebroside **8** in C_5D_5N.

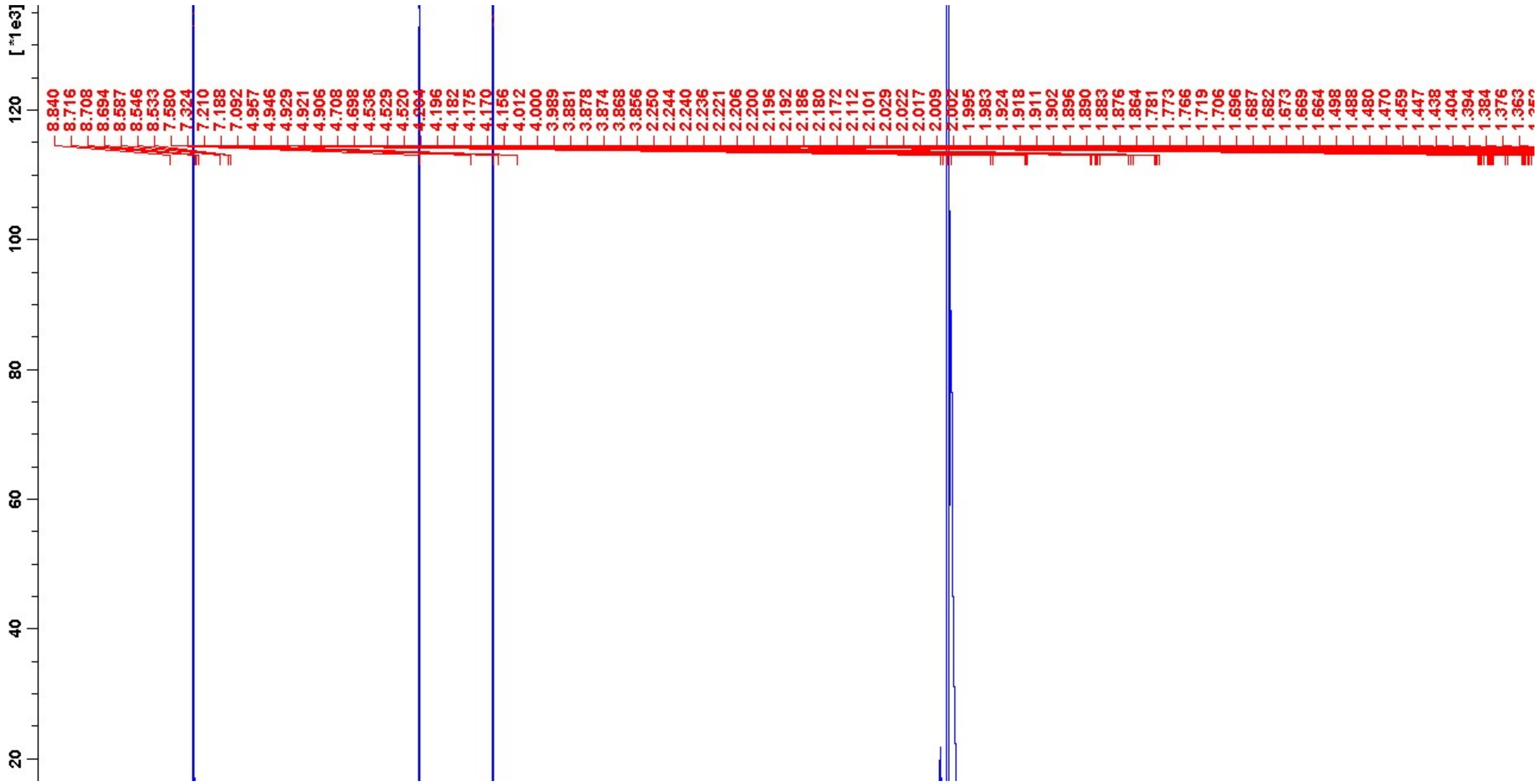

Figure S33. ^{13}C-NMR spectrum of cerebroside **8** in C_5D_5N.

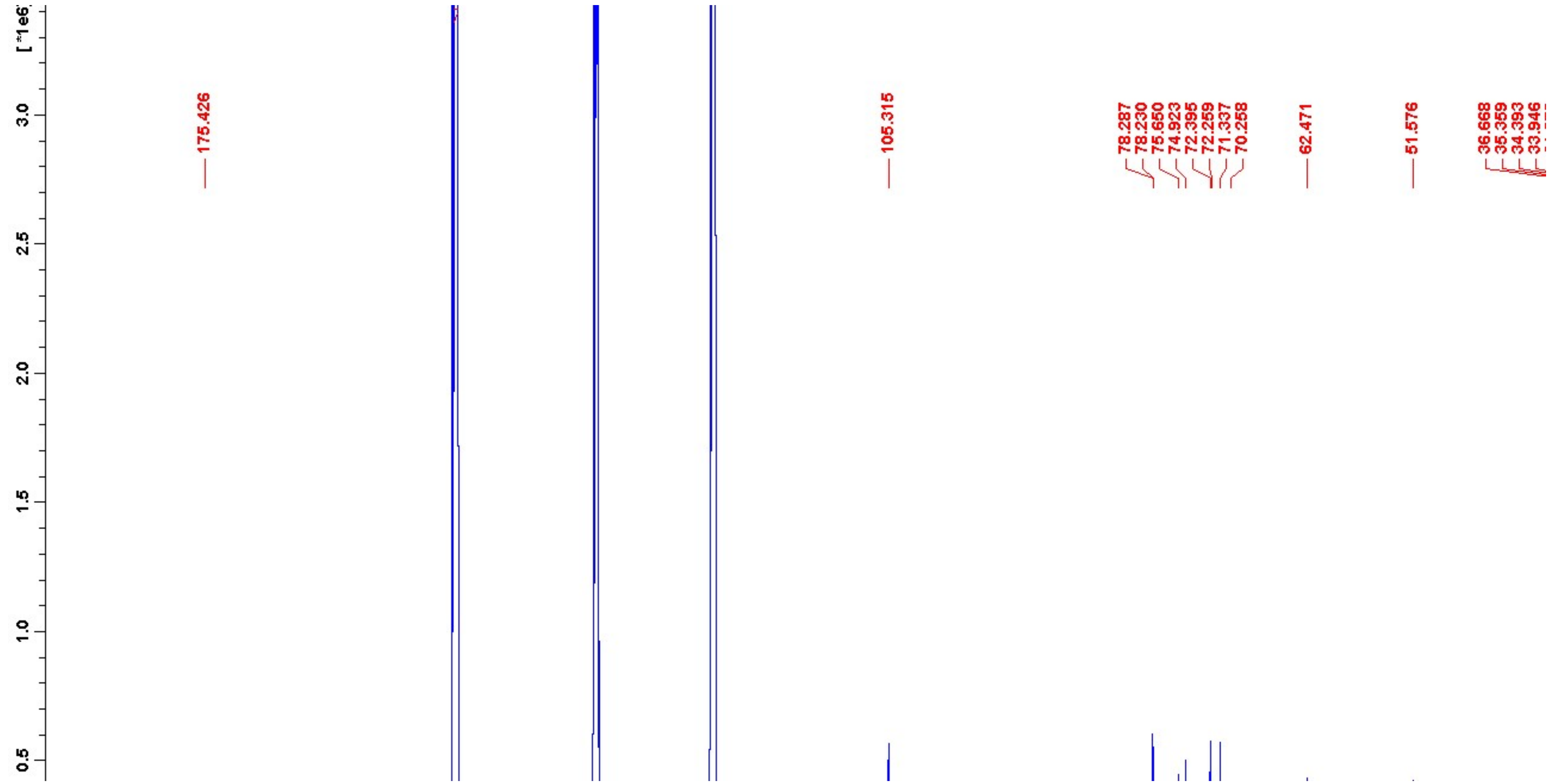

Figure S34. ^{1}H-^{1}H-COSY spectrum of cerebroside **8** in C_5D_5N.

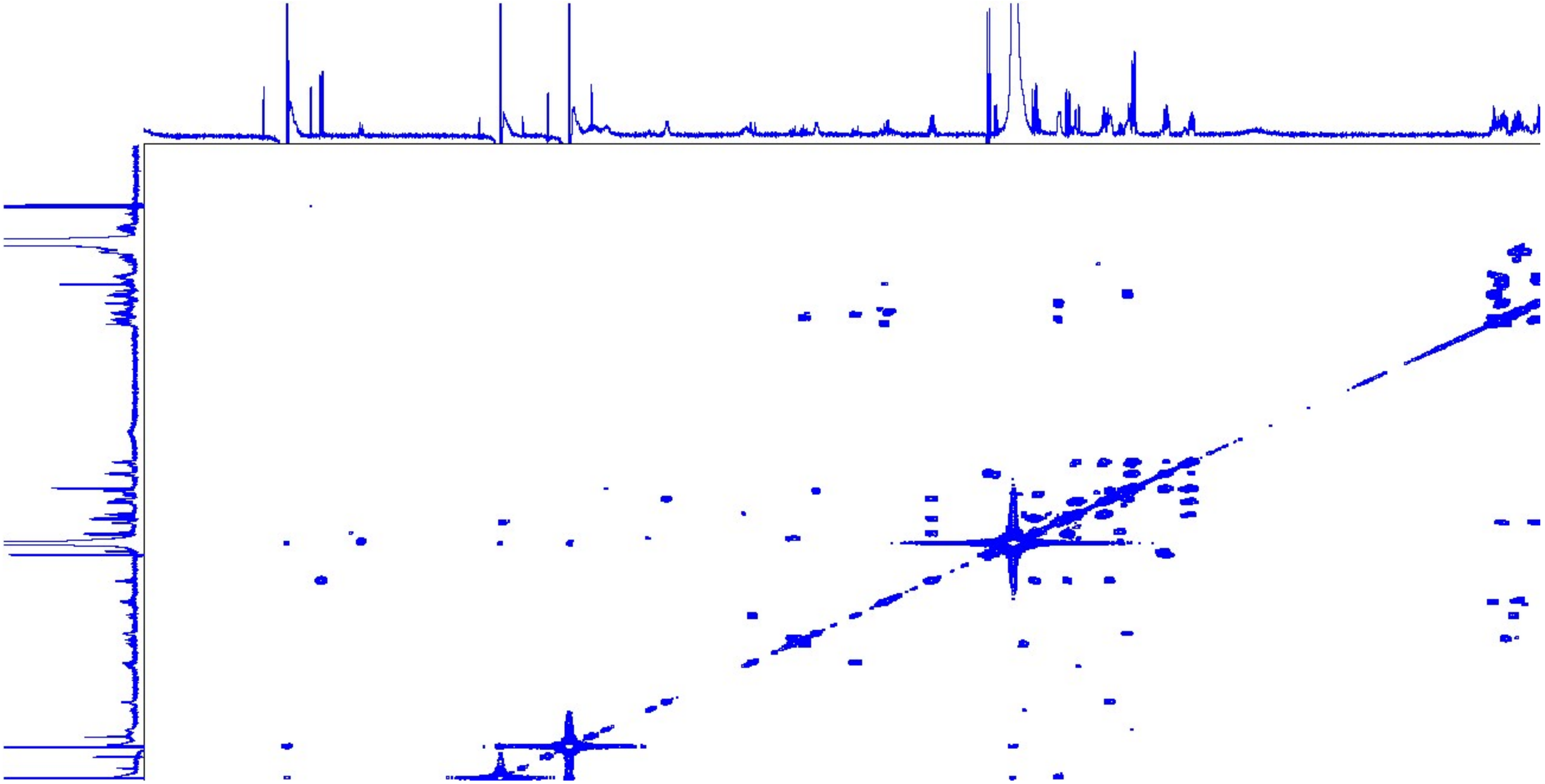

Figure S35. HSQC spectrum of cerebroside **8** in C_5D_5N.

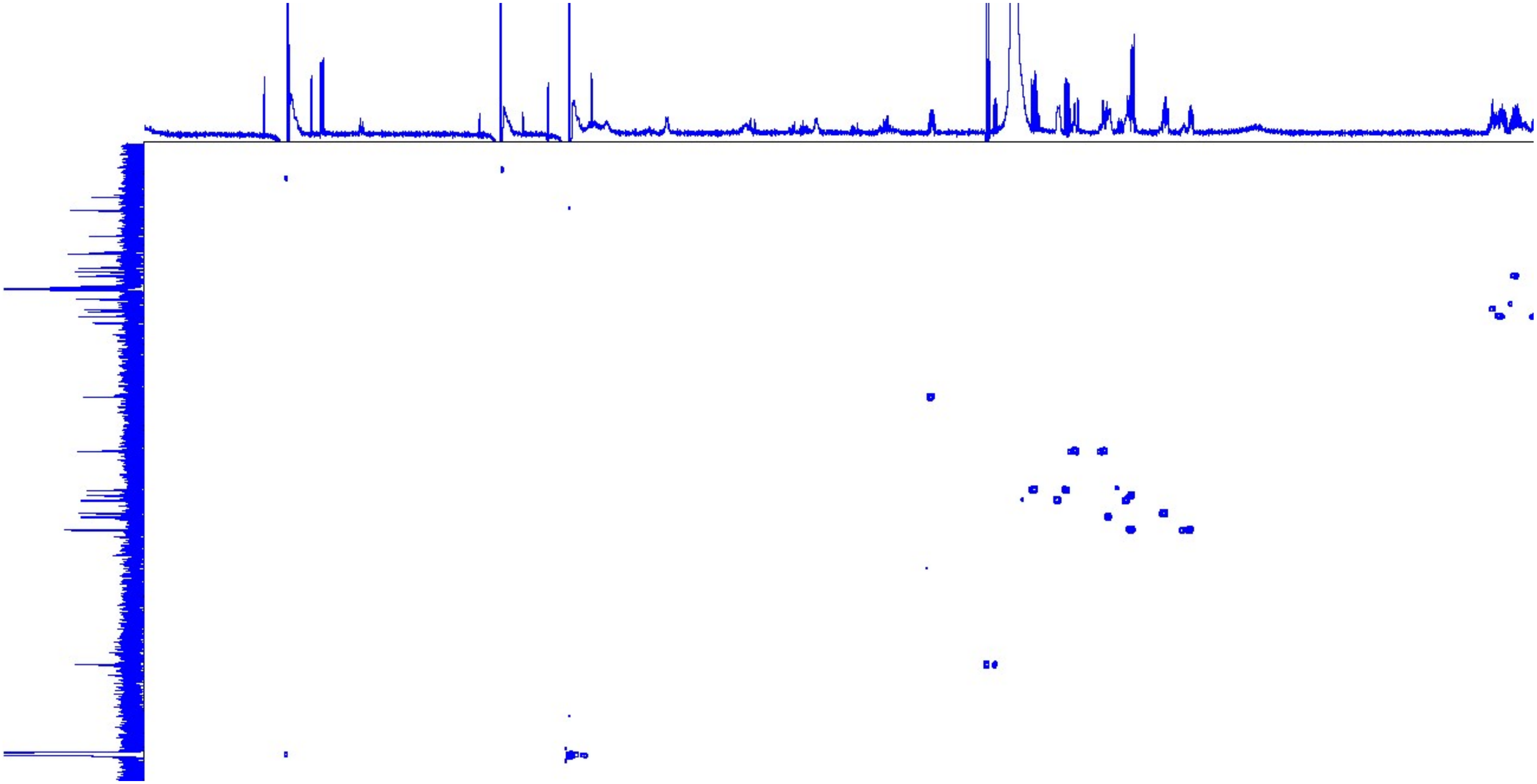

Figure S36. HMBC spectrum of cerebroside **8** in C_5D_5N.

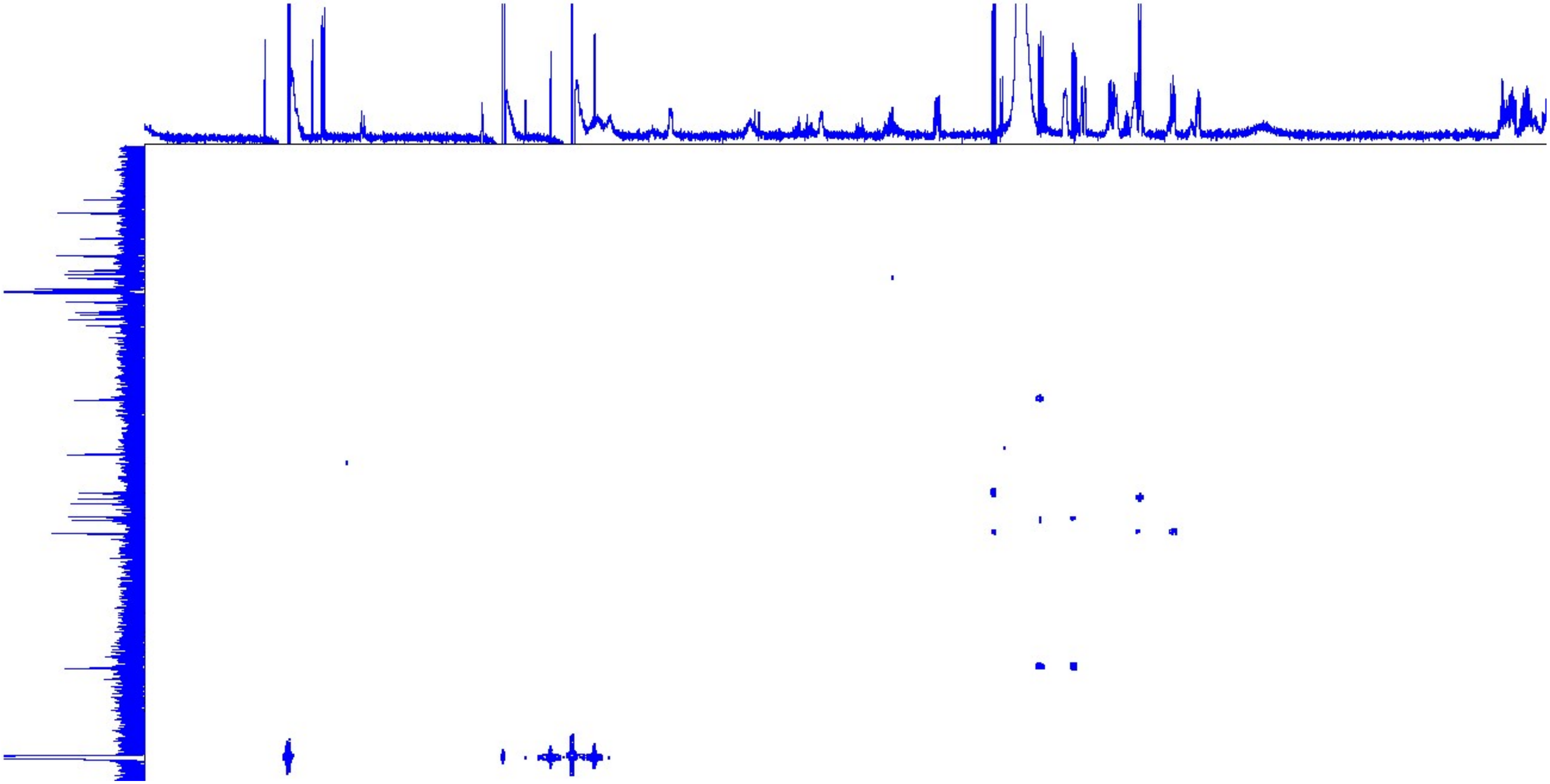

Figure S37. (–)-HRESIMS spectrum of cerebroside **9**.

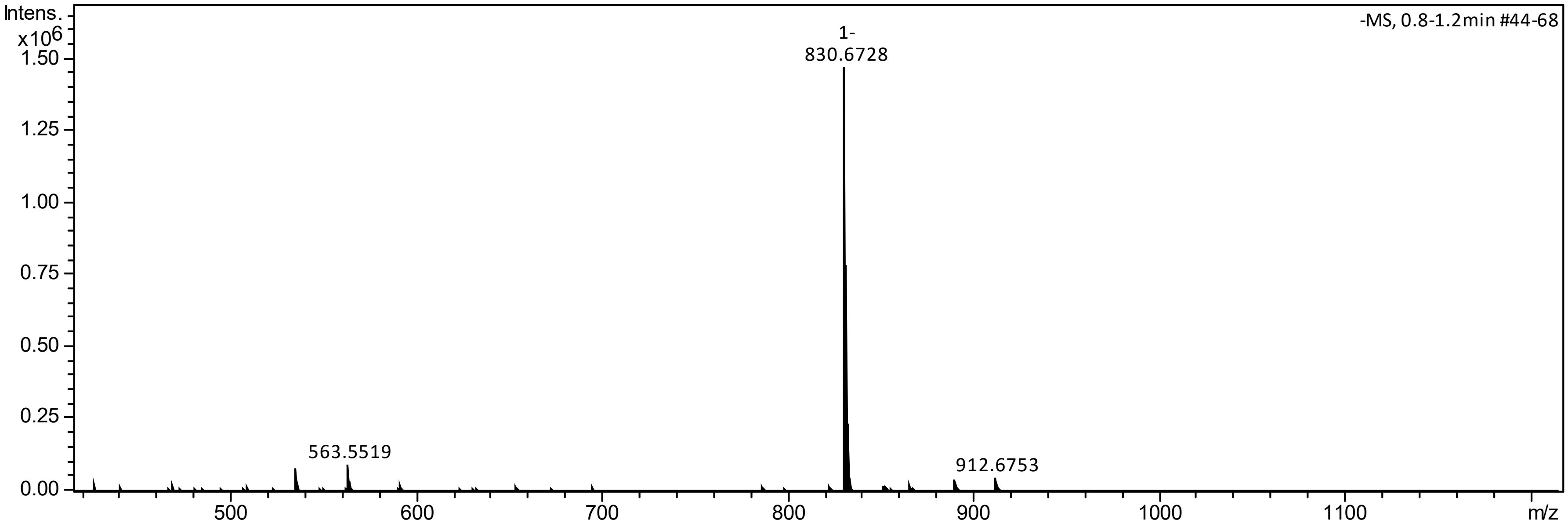

Figure S38. (+)-HRESIMS spectrum of cerebroside **9**.

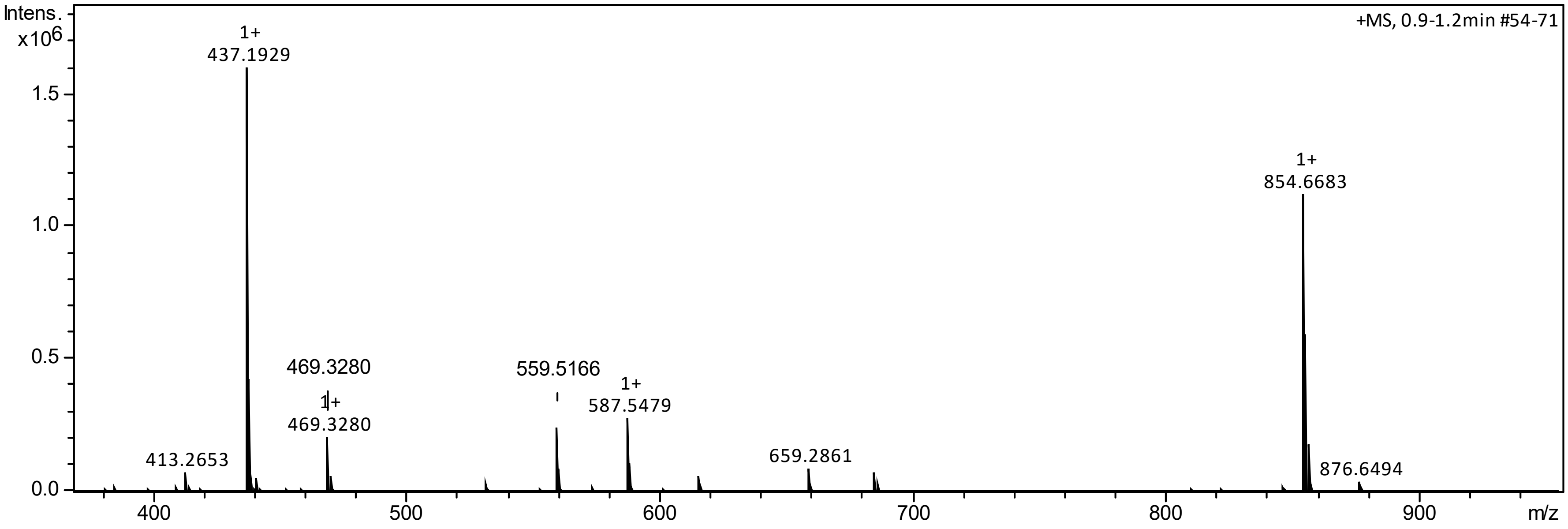

Figure S39. ^{1}H-NMR spectrum of cerebroside **9** in C_5D_5N.

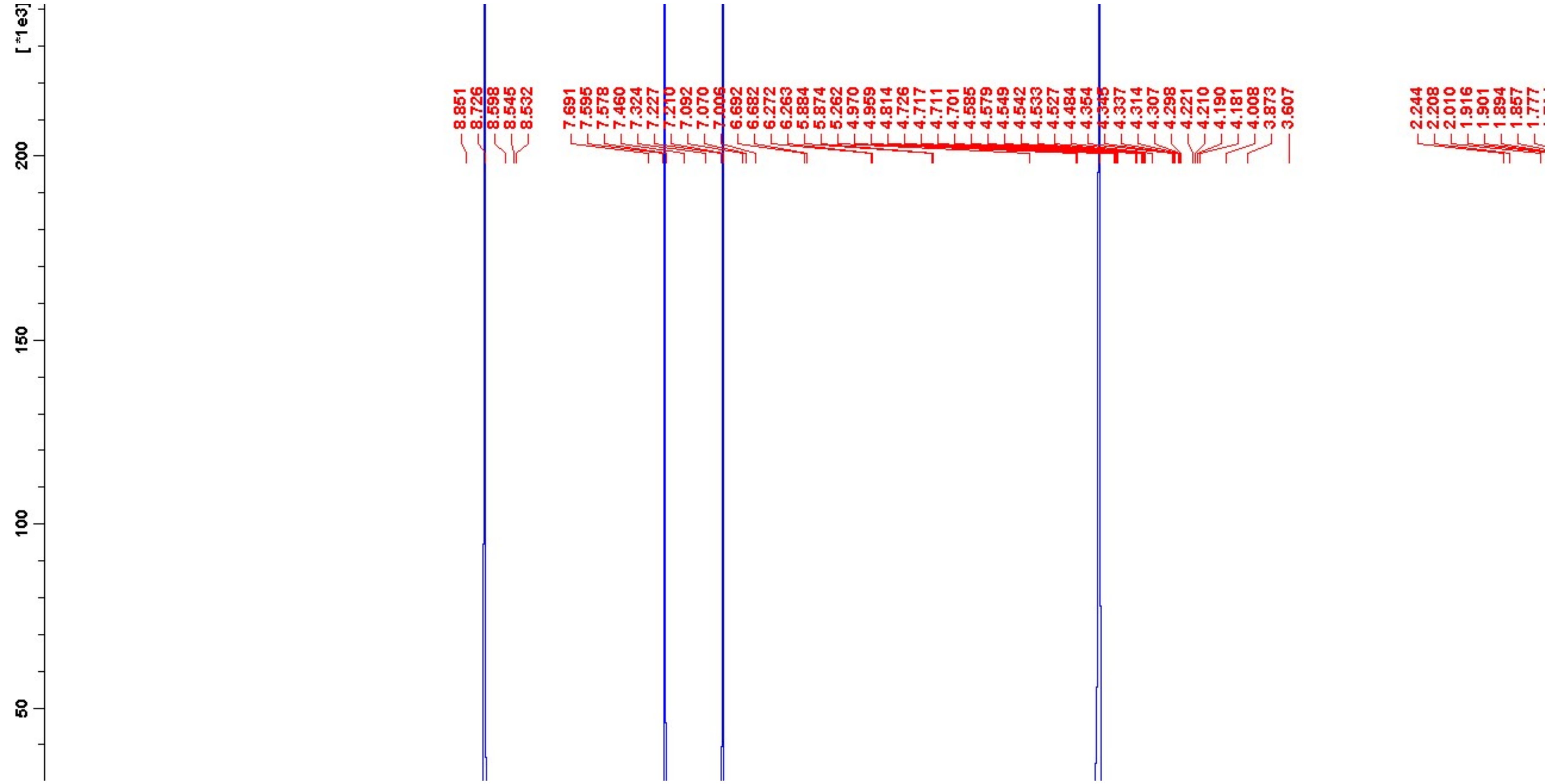

Figure S40. ^{13}C-NMR spectrum of cerebroside **9** in C_5D_5N.

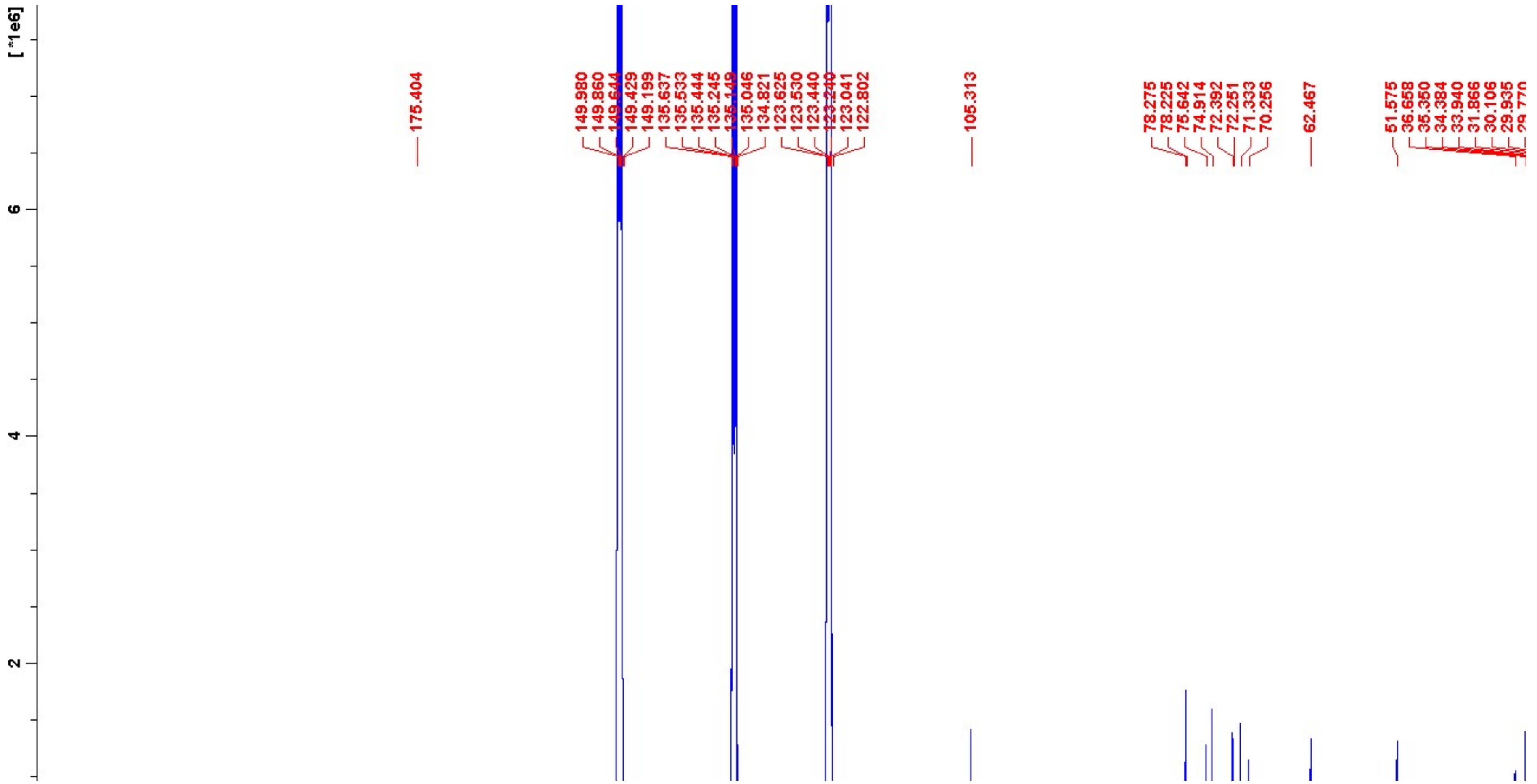

Figure S41. ^{1}H-^{1}H-COSY spectrum of cerebroside **9** in C_5D_5N.

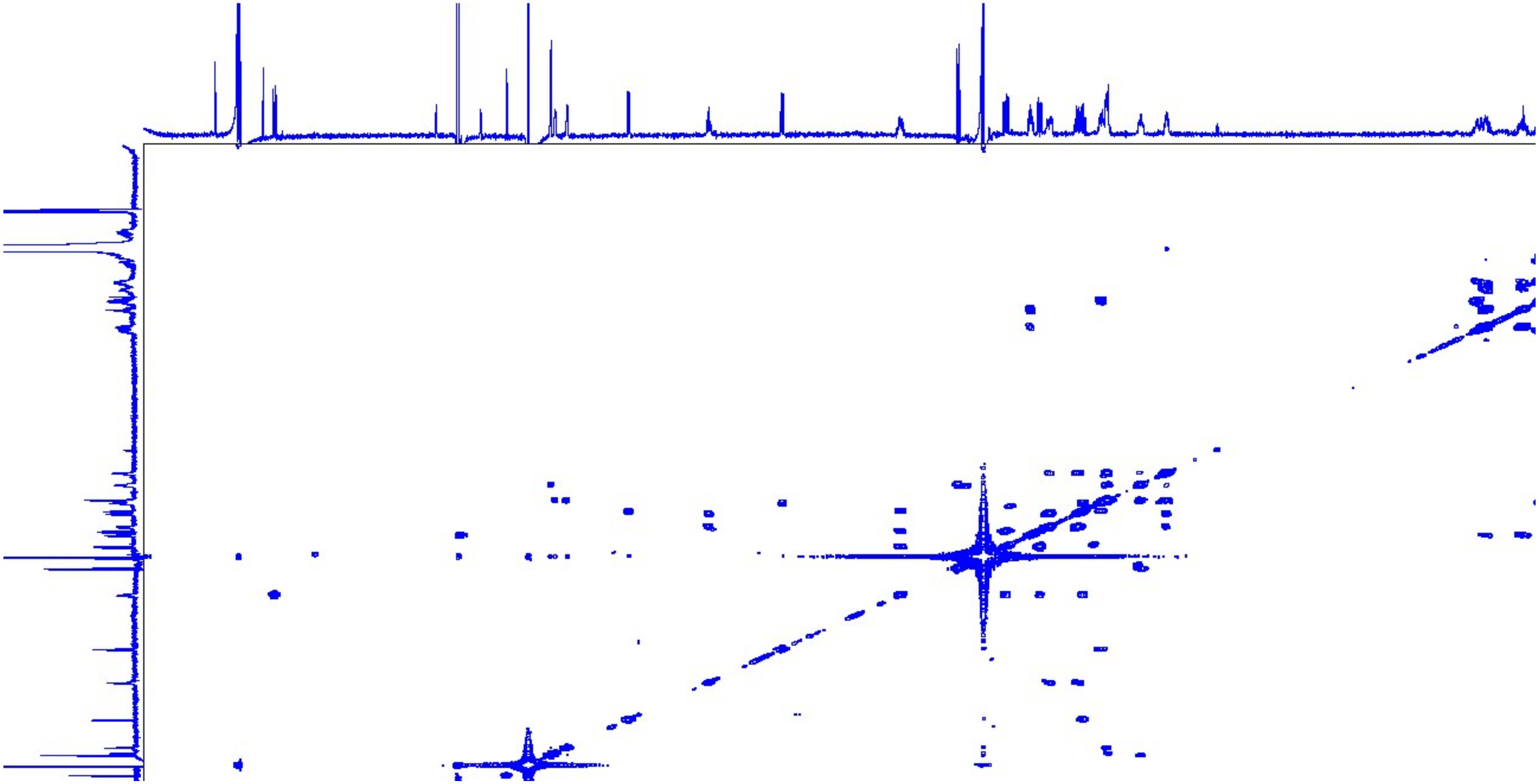

Figure S42. HSQC spectrum of cerebroside **9** in C_5D_5N.

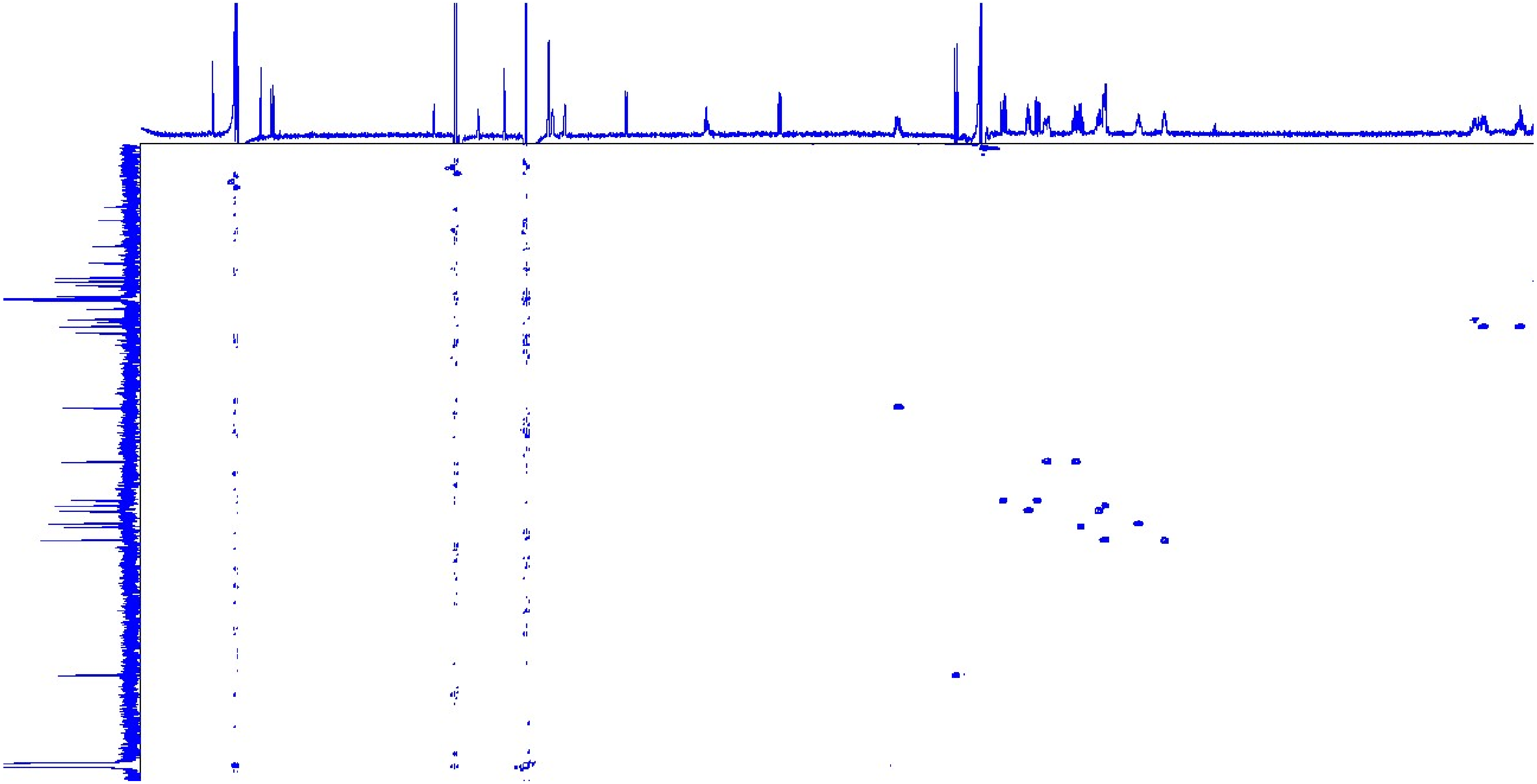

Figure S43. HMBC spectrum of cerebroside **9** in C_5D_5N.

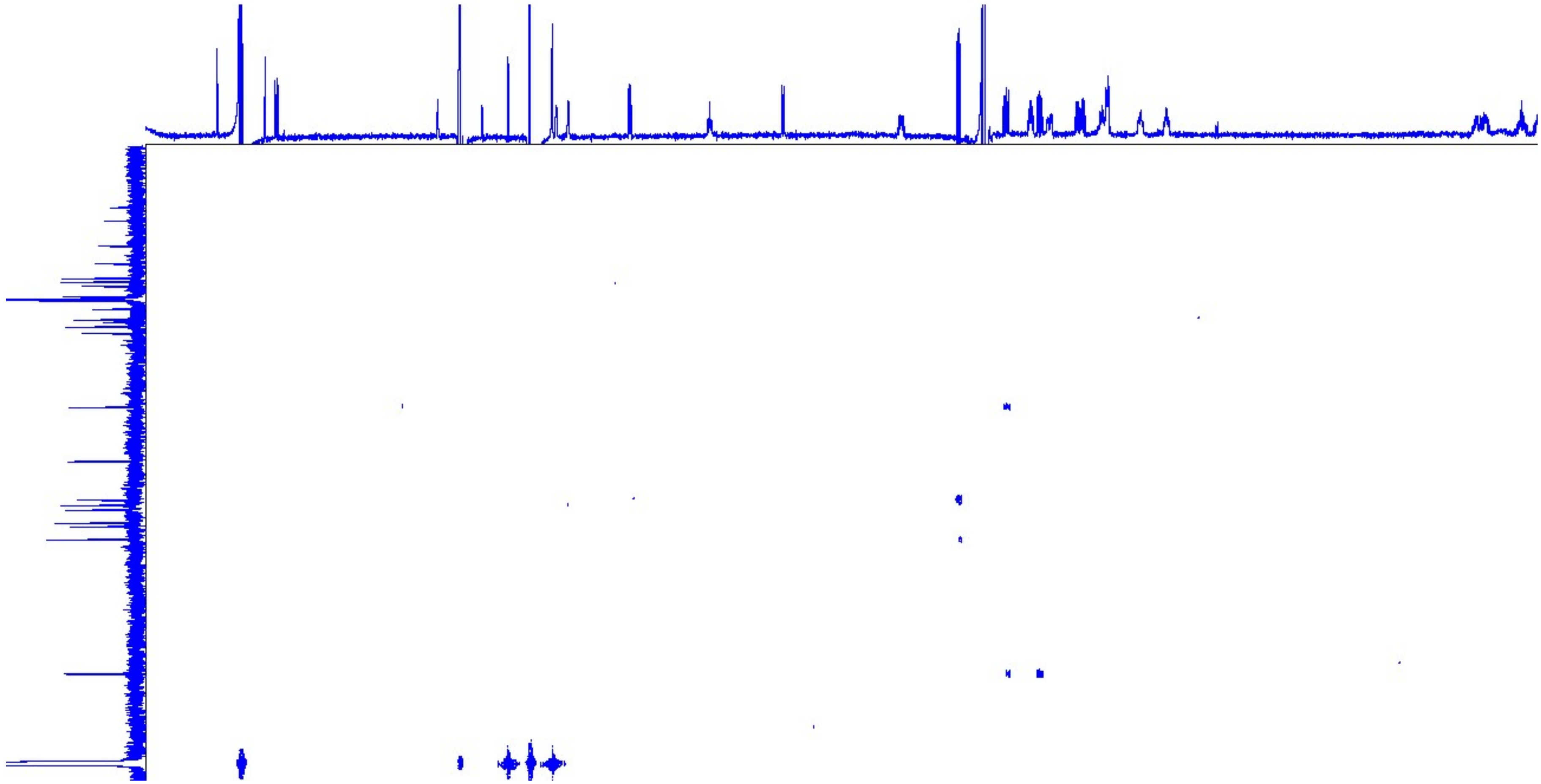

Supplement: Supplementary file 1 [file marinedrugs-20-00641-s001.zip › marinedrugs-1934363-SI.pdf]
